# Maternal hemoglobin concentrations across pregnancy and maternal and child health: a systematic review and meta‐analysis

**DOI:** 10.1111/nyas.14093

**Published:** 2019-04-17

**Authors:** Melissa F. Young, Brietta M. Oaks, Sonia Tandon, Reynaldo Martorell, Kathryn G. Dewey, Amanda S. Wendt

**Affiliations:** ^1^ Hubert Department of Global Health Emory University Atlanta Georgia; ^2^ Department of Nutrition and Food Sciences University of Rhode Island Kingston Rhode Island; ^3^ Department of Nutrition University of California, Davis Davis California; ^4^ Heidelberg Institute of Global Health University of Heidelberg Heidelberg Germany

**Keywords:** hemoglobin, anemia, pregnancy, review, birth outcomes

## Abstract

Maternal anemia is a well‐recognized global health problem; however, there remain questions on specific hemoglobin (Hb) thresholds that predict health risk or protection for mother and child. We conducted a systematic review and meta‐analysis to examine the associations of maternal Hb concentrations with a range of maternal and infant health outcomes, accounting for the timing of measurement (preconception, and first, second, and third trimesters), etiology of anemia, and cutoff category. The systematic review included 272 studies and the meta‐analysis included 95 studies. Low maternal Hb (<110 g/L) was associated with poor birth outcomes (low birth weight, preterm birth, small‐for‐gestational‐age (SGA), stillbirth, and perinatal and neonatal mortality) and adverse maternal outcomes (postpartum hemorrhage, preeclampsia, and blood transfusion). High maternal Hb (>130 g/L) was associated with increased odds of SGA, stillbirth, preeclampsia, and gestational diabetes. Relationships varied by the timing of measurement and cutoff category (stronger associations with lower cutoffs); limited data were available on anemia etiology. There were insufficient data for other maternal outcomes and long‐term child health outcomes. Current data are insufficient for determining if revisions to current Hb cutoffs are required. Pooled high‐quality individual‐level data analyses, as well as prospective cohort studies, would be valuable to inform the reevaluation of Hb cutoffs.

## Introduction

Anemia is an important global health problem affecting nearly 529 million women of reproductive age, including 38% of all pregnant women.^1^ Anemia during pregnancy is a key contributor to maternal mortality and poor birth outcomes in both low‐ and high‐income countries.^2^, ^3^ Reducing maternal anemia is widely recognized as central to the health of women and children. Current WHO global targets call for a 50% reduction of anemia in women of reproductive age by 2025.^4^ However, there remain key gaps in our understanding of the association of maternal hemoglobin (Hb) concentration on maternal and child health.

Several prior systematic reviews have examined associations between maternal anemia and birth outcomes.^5^, ^6^, ^7^, ^8^, ^9^, ^10^, ^11^, ^12^ Across the studies reviewed, there is high heterogeneity in the definition of anemia, the timing of exposure, etiology of anemia, and in the definitions of normal or healthy reference ranges for Hb for comparison. Defining anemia during pregnancy is complicated by changes in Hb concentrations in response to increases in blood volume and iron needs of the fetus.^13^ Hb concentrations reach a low point in the second trimester of pregnancy due to an increase in plasma volume that is not equaled by a proportionate increase in red cell volume expansion. Before 2016, the World Health Organization (WHO) defined anemia during pregnancy as a Hb concentration below 110 g/L at any time point in the pregnancy.^14^ However, 2016 WHO recommendations on antenatal care and the U.S. Centers for Disease Control and Prevention guidelines recommend trimester‐specific cutoffs for anemia (first trimester: <110 g/L; second trimester: <105 g/L; and third trimester: <110 g/L).^15^, ^16^ There is a wide range of Hb cutoffs used across studies, which may affect the likelihood of detecting relationships with birth outcomes.^5^ Some studies performed analyses using multiple cutoffs and reported that only more extreme cutoffs were significantly associated with adverse birth outcomes.^17^, ^18^, ^19^, ^20^ This is supported by a 2012 meta‐analysis of 12 studies which indicated that moderate to severe, but not mild maternal anemia was associated with an increased risk of small‐for‐gestational‐age (SGA).^6^ These issues are further complicated by lack of a clear consensus on normal or healthy reference ranges for Hb.

The timing of the measurement of maternal anemia is also variable across studies. This is problematic for meta‐analyses, as existing literature suggests a differential impact of maternal Hb concentration depending on the timing during pregnancy.^5^ For low Hb concentration, the link with adverse birth outcomes is more evident when Hb concentration is measured in early pregnancy.^19^, ^20^, ^21^ As summarized in a recent review, several studies have observed a U‐shaped curve for the risk of adverse birth outcomes with maternal Hb, that is, there is a higher risk of adverse outcomes among women with both low and high Hb concentrations.^5^ However, the consistency of this relationship varies based on the trimester of Hb assessment. High Hb concentration could be a marker of inadequate plasma volume expansion, which is likewise associated with adverse birth outcomes.^5^ Further research is needed to better understand these relationships; however, there is also a lack of consensus on the cutoff to define high Hb concentration, making it difficult to study how high Hb concentration relates to maternal and child health outcomes.

In addition to the important role of the timing during pregnancy, there is a growing emphasis on the importance of maternal nutritional status and anemia before pregnancy.^22^, ^23^, ^24^, ^25^ Compared with pregnancy, much less is known about the influence of maternal preconception Hb concentration on health outcomes.

Finally, the etiology of anemia is diverse, and it remains unclear how the relationship between anemia and maternal and child outcomes varies by cause of anemia. Scholl *et al*. report greater odds of both low birth weight and preterm birth associated with iron deficiency anemia (IDA) compared with non‐IDA.^26^ It is estimated that approximately 50% of anemia among nonpregnant and pregnant women is responsive to iron supplementation;^27^ however, at the national and subnational level, the percentage of anemia that is IDA has been shown to be extremely variable, ranging from <1% to 75%.^28^ Whether the etiology of anemia affects relationships between Hb concentrations and health outcomes merits closer examination.

Given the importance of maternal anemia across the globe, it is critical to come to a consensus on the definition of maternal anemia and whether it needs to account for the timing during pregnancy and etiology.

Our objective was to conduct a systematic review and meta‐analysis on the associations of maternal Hb concentrations with a range of maternal and infant health outcomes, accounting for the timing of measurement (preconception, and first, second, and third trimesters), etiology of anemia, and cutoff category to inform WHO guidelines on Hb concentration cutoffs.

## Methods

### Search strategy

We searched for studies that reported associations between maternal Hb concentrations or anemia, measured during preconception or pregnancy, and a range of maternal and child outcomes. Maternal outcomes included postpartum hemorrhage, preeclampsia, blood transfusion, prenatal and postpartum depression, and maternal mortality. Child health outcomes included both birth and long‐term outcomes, such as low birth weight (LBW; birth weight <2500 g), SGA (birth weight below the 10th percentile for gestational age or as defined by study authors), preterm birth (PTB; <37 weeks of gestation), stillbirth (as defined by study authors, e.g., fetal death, fetal death at or after 28 weeks gestation, birth of fetus at 22 gestational weeks, or later with no signs of life), perinatal mortality (defined as the sum of fetal deaths (28 weeks or more gestation) and infant deaths occurring less than 7 days after birth), neonatal mortality (death within first 28 days of life), infant mortality (death within the first year of life), birth weight and length, head circumference, child development, diabetes, hypertension, blood pressure, and cardiovascular disease. With the help of the Emory University Library staff, we searched PubMed and Cochrane Review in June 2017 and updated the search in October 2018 with no restrictions for language, date, or population (Table S1, online only). Reference lists from previous systematic reviews were also screened to identify any studies not initially included in our search strategy. Studies were eligible for inclusion in the systematic review and meta‐analysis if they reported an association between maternal Hb concentration or anemia, during preconception or pregnancy, and one or more of the above key outcomes. We limited the meta‐analyses to peer‐reviewed studies that adjusted for one or more confounders.

### Data extraction and quality assessment

Screening and article selection was conducted using Covidence systematic review software (Veritas Health Innovation, Melbourne, Victoria, Australia) to organize search results from PubMed and Cochrane Review. The abstract screening was independently completed in duplicate by two authors and conflicts were reviewed and resolved by a third member. A full‐text review was likewise completed in duplicate by two team members and conflicts were resolved by a committee of five team members. Data extracted included country, year of study, study design, study participant characteristics, the timing of exposure, Hb measurement, outcomes, iron status/deficiency, infection status, Hb laboratory method, confounders, and the measure of association. A back check on 10% of all data extractions was conducted by a second team member. We attempted to contact corresponding authors for any missing data not presented in the publication (e.g., definition of anemia, the timing of measurement, included covariates, or the measure of association).

### Data management and analyses

Selected characteristics and results of included studies were abstracted including country, year of study, study design, the timing of measurement, outcome measure, covariates, and an adjusted and unadjusted measure of associations with 95% confidence intervals. For the meta‐analysis, data were stratified by the timing of measurement: preconception, first trimester (≤13 weeks), second trimester (14−26 weeks), and third trimester (≥27 weeks). Studies in which the timing of Hb concentration was unclear (e.g., the lowest Hb concentration during pregnancy and Hb concentration at enrollment without specifying gestational age at enrollment) or unrestricted were only considered in the overall pregnancy estimate. Maternal Hb concentration was examined both as a continuous and a categorical variable (as defined in the publications). Because evidence suggests that the association between maternal Hb concentrations and outcomes is not linear, meta‐analyses using continuous Hb measures are difficult to summarize and interpret; thus, data from these studies are presented in a table format only. Summary estimates by the timing were constructed using all low Hb cutoffs (<110 g/L) and all high Hb cutoffs (>130 g/L). Definitions of anemia and elevated Hb varied across studies, thus separate analyses were conducted by cutoff used (≤70, ≤80, ≤90, ≤100, ≤110, ≥120, ≥130, and ≥140 g/L) to create summary estimates. Categories were cumulative, for example, the ≤100 g/L category included studies using the ≤100g/L cutoff or any subset where all Hb values were ≤100 g/L. Definitions of study exposure for all studies included in the meta‐analysis are summarized in Tables S2−S5 (online only). Reference groups varied and are specified for all studies included in the meta‐analysis in Figures S1–S10 (online only). It is important to note that the reference groups varied widely across studies, with some providing a range (e.g., 110−119 g/L) and others a cut off (≥110 g/L) with little consensus on what represents a “healthy” reference value. Where data were available, stratified analyses were conducted specifically for iron deficiency (ID) and by the level of reported baseline maternal ID. Meta‐analyses were performed if there were at least three studies with the same exposure, outcome, study design, and timing. All analyses were done using STATA version 14.2 (Stata Corp. 2015, College Station, TX) using the “admetan” command. Heterogeneity was assessed by *Q*‐ and *I* ^2^‐statistics. *I* ^2^ values over 50% and a Q‐statistic *P* value of >0.10 indicated substantial heterogeneity among studies. As most analyses showed significant heterogeneity, all meta‐analyses were conducted using random effects models and the inverse‐variance method for weighting. Results were considered statistically significant if *P* < 0.05.

## Results

**Figure 1:**
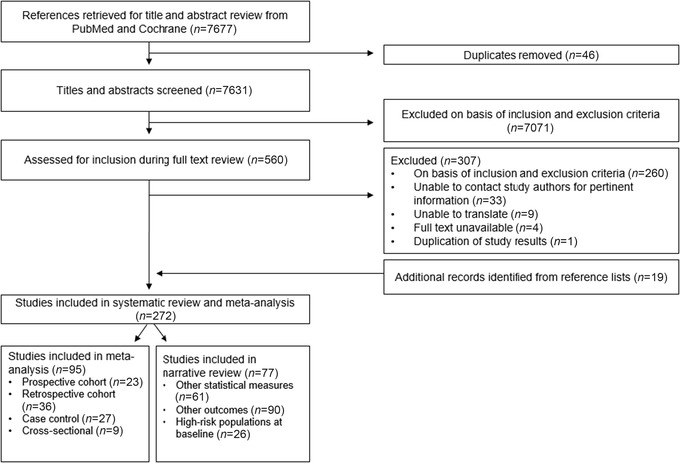
Flow of study selection.

Our search strategy identified 7677 studies in October 2018, of which 46 duplicates were removed (Fig. 1). Abstracts of 7631 studies were screened for eligibility based on inclusion and exclusion criteria. We completed a full text review on 560 studies; and 307 studies were excluded on the basis of inclusion and exclusion criteria (*n* = 293), inability to translate (*n* = 9), duplication of study results (*n* = 1), or unavailable full text (*n* = 4). We included an additional 19 studies missed by our search strategy by reviewing the reference lists of prior systematic reviews. In total, 272 studies were included in our review and 95 studies met the criteria for inclusion in the meta‐analyses (Fig. 1),^17^, ^21^, ^26^, ^29^, ^30^, ^31^, ^32^, ^33^, ^34^, ^35^, ^36^, ^37^, ^38^, ^39^, ^40^, ^41^, ^42^, ^43^, ^44^, ^45^, ^46^, ^47^, ^48^, ^49^, ^50^, ^51^, ^52^, ^53^, ^54^, ^55^, ^56^, ^57^, ^58^, ^59^, ^60^, ^61^, ^62^, ^63^, ^64^, ^65^, ^66^, ^67^, ^68^, ^69^, ^70^, ^71^, ^72^, ^73^, ^74^, ^75^, ^76^, ^77^, ^78^, ^79^, ^80^, ^81^, ^82^, ^83^, ^84^, ^85^, ^86^, ^87^, ^88^, ^89^, ^90^, ^91^, ^92^, ^93^, ^94^, ^95^, ^96^, ^97^, ^98^, ^99^, ^100^, ^101^, ^102^, ^103^, ^104^, ^105^, ^106^, ^107^, ^108^, ^109^, ^110^, ^111^, ^112^, ^113^, ^114^, ^115^, ^116^, ^117^, ^118^, ^119^, ^120^, ^121^ while the remaining 177 studies are summarized in table format only. A summary of all studies included in the meta‐analyses is provided in Table S2 (online only). Additional child health outcome data are summarized in Table S3 (online only) for outcomes with too few studies to conduct a meta‐analysis and in Table S6 (online only) for outcomes that met inclusion criteria but used statistical measures incompatible with pooling in meta‐analyses. Likewise, additional maternal health outcome data are presented in Table S4 (online only) for maternal outcomes with an inadequate number of studies to conduct a meta‐analysis and in Table S7 (online only) for studies using statistical measures incompatible with pooling. Studies conducted in high‐risk populations are presented in Table S5 (online only). Based on available data presented for certain outcomes of interest in studies, 24 studies were included in both the meta‐analyses and narrative review.

### Study characteristics

Of the 95 studies included in the meta‐analyses, there were 23 prospective cohorts, 36 retrospective cohorts, 27 case–control, and 9 cross‐sectional studies. Studies were published from 1984 to 2018 and took place in 37 countries. Included studies come from all six WHO geographical regions: Southeast Asia (*n* = 16), Europe (*n* = 21), Western Pacific (*n* = 17), the Americas (*n* = 13), Africa (*n* = 14), and Eastern Mediterranean (*n* = 10). Four large multicenter studies were conducted in multiple WHO regions; one study was conducted in four high‐income countries across the European and Western Pacific regions,^69^ one was conducted in 24 countries across the regions of Africa, Southeast Asia, and the Americas,^37^ one was conducted in 16 countries across the regions of the Eastern Mediterranean, Africa, Southeast Asia, and the Americas,^118^ and another was conducted in 29 countries across the African, Southeast Asian, Eastern Mediterranean, and Western Pacific regions.^76^ Study sample sizes ranged from 102 to 2,722,274, and maternal age, education level, body mass index, and parity were commonly adjusted for in statistical analyses. Presented results are limited to childbirth and maternal health outcomes as there were insufficient data to assess long‐term outcomes, such as a chronic disease in the meta‐analyses. Only four studies measured preconception Hb concentrations: Ronnenberg *et al*., Zhang *et al*., and Yi *et al*. measured Hb concentrations a median of 5 weeks prior to conception, 1.5 months prior to conception, and on or before the first day of the last menstrual cycle, respectively, while the preconception timing for Maghsoudlou *et al*. is unclear (1 year before pregnancy).^17^, ^82^, ^98^, ^111^

## Child health outcomes

Data were available to conduct meta‐analyses for the following birth outcomes: (1) low birth weight (*n* = 41); (2) preterm birth (*n* = 40); (3) small for gestational age (*n* = 25); (4) stillbirth (*n* = 19); (5) perinatal mortality (*n* = 11); and (6) neonatal mortality (*n* = 5). Results for these outcomes are presented by (1) time period: preconception, and first, second, and third trimesters; (2) cutoff: Hb ≤70, ≤80, ≤90, ≤100, ≥120, ≥130, and ≥140 g/L; and (3) overall estimate throughout pregnancy analysis (when data were available for each comparison). Table 1 summarizes associations with low maternal Hb and child outcomes and Table 2 summarizes associations with high maternal Hb. Several studies included in our review used statistical methods incompatible with the inclusion in the overall meta‐analyses; these studies are summarized in Table S6 (online only). Data were stratified by Hb cutoff and timing. There were insufficient data to further examine Hb cutoff within each of the trimesters, so these two factors were examined separately. In the tables, blank cells indicate that no studies explored the association and estimates with an asterisk were based on only 1−2 studies. The number of individual studies assessing maternal Hb concentration and birth outcomes in the meta‐analyses for each Hb concentration is summarized in Table S8 (online only).

**Table 1:** Meta‐analysis of the association between low maternal Hb and child outcomes

	LBW OR (95% CI)	PTB OR (95% CI)	SGA OR (95% CI)	Stillbirth OR (95% CI)	Perinatal mortality OR (95% CI)	Neonatal mortality OR (95% CI)
**Timing: low maternal Hb (<110 g/L) by timing**						
Preconception	**1.72 (1.31−2.26)** *	1.04 (0.97−1.12)	**1.79 (1.39**−**2.31)** *	1.23 (0.39−3.86)*		
First trimester	**1.23 (1.07−1.41)**	**1.28 (1.17**−**1.40)**	1.08 (0.94−1.25)	1.42 (0.39−5.25)*	1.61 (0.80−3.24)	**3.13 (1.76**−**5.57)** *
Second trimester	1.14 (0.78−1.68)	**1.37 (1.15**−**1.63)**	1.14 (0.96−1.37)	**2.22 (1.36**−**3.65)**	1.38 (0.88−2.15)*	
Third trimester	**1.65 (1.39**−**1.96)**	**1.45 (1.23**−**1.71)**	0.90 (0.73−1.10)	1.82 (0.97−3.42)	1.27 (0.97−1.67)	
**Cutoff: low maternal Hb at any time during pregnancy by cutoff**						
≤70 g/L	**2.97 (1.85**−**4.75)**	**3.72 (1.53**−**9.03)** *	**1.29 (1.15**−**1.43)** *	**3.87 (1.88**−**8.06)**	**4.41 (2.21**−**8.81)** *	
≤80 g/L	**2.77 (1.91**−**4.02)**	**2.89 (1.71**−**4.89)**	**1.39 (1.22**−**1.60)**	**2.84 (1.27**−**6.37)**	**3.82 (2.34**−**6.24)**	1.10 (0.29−4.21)*
≤90 g/L	**2.48 (1.90**−**3.23)**	**1.73 (1.38**−**2.16)**	**1.32 (1.20**−**1.44)**	**2.47 (1.52**−**4.01)**	**2.66 (1.28**−**5.55)**	1.10 (0.29−4.21)*
≤100 g/L	**1.49 (1.33**−**1.67)**	**1.47 (1.32**−**1.65)**	**1.13 (1.01**−**1.27)**	**1.88 (1.27**−**2.78)**	**1.94 (1.38**−**2.47)**	**1.57 (1.19**−**2.08)**
≤110 g/L	**1.42 (1.31**−**1.55)**	**1.36 (1.26**−**1.46)**	**1.08 (1.00**−**1.18)**	**1.49 (1.15**−**1.92)**	**1.73 (1.32**−**2.26)**	**1.49 (1.19**−**1.87)**
Overall estimate** <110 g/L	**1.42 (1.31**−**1.55)**	**1.36 (1.26**−**1.46)**	**1.08 (1.00**−**1.18)**	**1.49 (1.15**−**1.92)**	**1.73 (1.32**−**2.26)**	**1.49 (1.19**−**1.87)**

notes: The sample size for individual studies is provided in Table S2 (online only), and the total number of studies included in each Hb cutoff effect estimate and timing are specified in Table S8 (online only). A number of studies included for an overall estimate (<110 g/L): LBW (36), PTB (33), SGA (21), stillbirth (21), perinatal mortality (11), and neonatal mortality (5). Reference groups for individual studies included in the meta‐analysis were highly variable, with some providing a range (e.g., 110−119 g/L) and others a cut off (≥110 g/L), with little consensus on what represents a “healthy” reference value. Reference groups for all studies included in meta‐analyses can be found in Figures S1–S6 (online only). Categories are cumulative; for example, the ≤100 g/L category includes studies using the ≤100 g/L cutoff or any subset where all Hb values were ≤100 g/L. Definitions: LBW, low birth weight (<2500 g); PTB, preterm birth (birth at <37 weeks gestation); SGA, small for gestational age (birth weight below the 10th percentile for gestational age or as defined by study authors); stillbirth (as defined by study authors); perinatal mortality (defined as the sum of fetal deaths (28 weeks or more gestation), and infant deaths occurring less than 7 days after birth); neonatal mortality (death within 28 days of life); first trimester: ≤13 weeks; second trimester: 14–26 weeks; third trimester: ≥27 weeks. Bold values are statically significant with *P* < 0.05.

^*^Summary estimates from meta‐analyses of <3 studies.

^**^Overall estimates using Hb concentrations measured at any point during pregnancy.

**Table 2:** Meta‐analysis of association between high maternal Hb and child outcomes

	LBW OR (95% CI)	PTB OR (95% CI)	SGA OR (95% CI)	Stillbirth OR (95% CI)
**Timing: high maternal Hb (>130 g/L) by timing**				
Preconception	1.04 (0.93−1.16)*	1.06 (1.00−1.13)*	1.03 (0.97−1.10)*	0.83 (0.52−1.33)*
First trimester	1.34 (0.52−3.46)*	0.93 (0.84−1.03)	1.12 (0.99−1.27)	1.28 (0.96−1.70)*
Second trimester	**1.40 (1.02**−**1.93)** *	1.07 (0.83−1.39)	**1.54 (1.31**−**1.81)** *	
Third trimester	**0.58 (0.45**−**0.74)** *	0.92 (0.55−1.56)*	1.08 (0.92−1.27)*	**2.31 (1.30**−**4.10)** *
**Cutoff: high maternal Hb at any time during pregnancy by cutoff**				
≥120 g/L**	1.41 (0.94−2.13)	1.16 (0.97−1.38)	**1.19 (1.08**−**1.31)**	**1.38 (1.11**−**1.73)**
≥130 g/L	1.61 (0.82−3.16)	1.14 (0.95−1.37)	**1.22 (1.10**−**1.35)**	**1.59 (1.14**−**2.22)**
≥140 g/L	1.37 (0.44−4.23)	1.10 (0.84−1.46)	1.18 (0.97−1.42)	**2.30 (1.38**−**3.85)**
≥150 g/L	0.78 (0.19−3.18)*	1.01 (0.55−1.85)	0.92 (0.55−1.56)*	
≥160 g/L	0.78 (0.19−3.18)*	0.42 (0.14−1.29)*	0.61 (0.22−1.56)*	
Overall estimate*** >130 g/L	1.80 (0.86−3.77)	1.17 (0.94−1.45)	**1.22 (1.08**−**1.37)**	**1.88 (1.21**−**2.91)**

notes: The sample size for individual studies is provided in Table S2 (online only), and the total number of studies included in each Hb cut‐off effect estimate and timing are specified in Table S8 (online only). A number of studies included for an overall estimate (>130): LBW (5), PTB (10), SGA (4), and stillbirth (4). Reference groups for individual studies included in the meta‐analysis were highly variable, with some providing a range (e.g., 110−119 g/L) and others a cut off (≥110), with little consensus on what represents a “healthy” reference value. Reference groups for all studies included in meta‐analyses can be found in Figures S1−S4 (online only). Categories are cumulative; for example, the ≥140 g/L category includes studies using the ≥140 g/L cutoff or any subset where all Hb values were ≥140 g/L. Definitions: LBW, low birth weight (<2500 g); PTB, preterm birth (birth at <37 weeks gestation); SGA, small‐for‐gestational‐age (birth weight below the 10th percentile for gestational age or as defined by study authors); stillbirth (as defined by study authors); perinatal mortality (defined as the sum of fetal deaths (28 weeks or more gestation) and infant deaths occurring less than 7 days after birth); first trimester: ≤13 weeks; second trimester: 1–26 weeks; third trimester: ≥27 weeks. Bold values are statically significant with *P* < 0.05.

^*^Summary estimates from meta‐analyses of <3 studies.

^**^Summary estimates for ≥120 g/L presented, but high Hb defined as ≥130 g/L for inclusion in meta‐analysis summary estimates.

^***^Overall estimates using Hb concentrations measured at any point during pregnancy.

### Low birth weight

#### Hb cutoff

**Figure 2:**
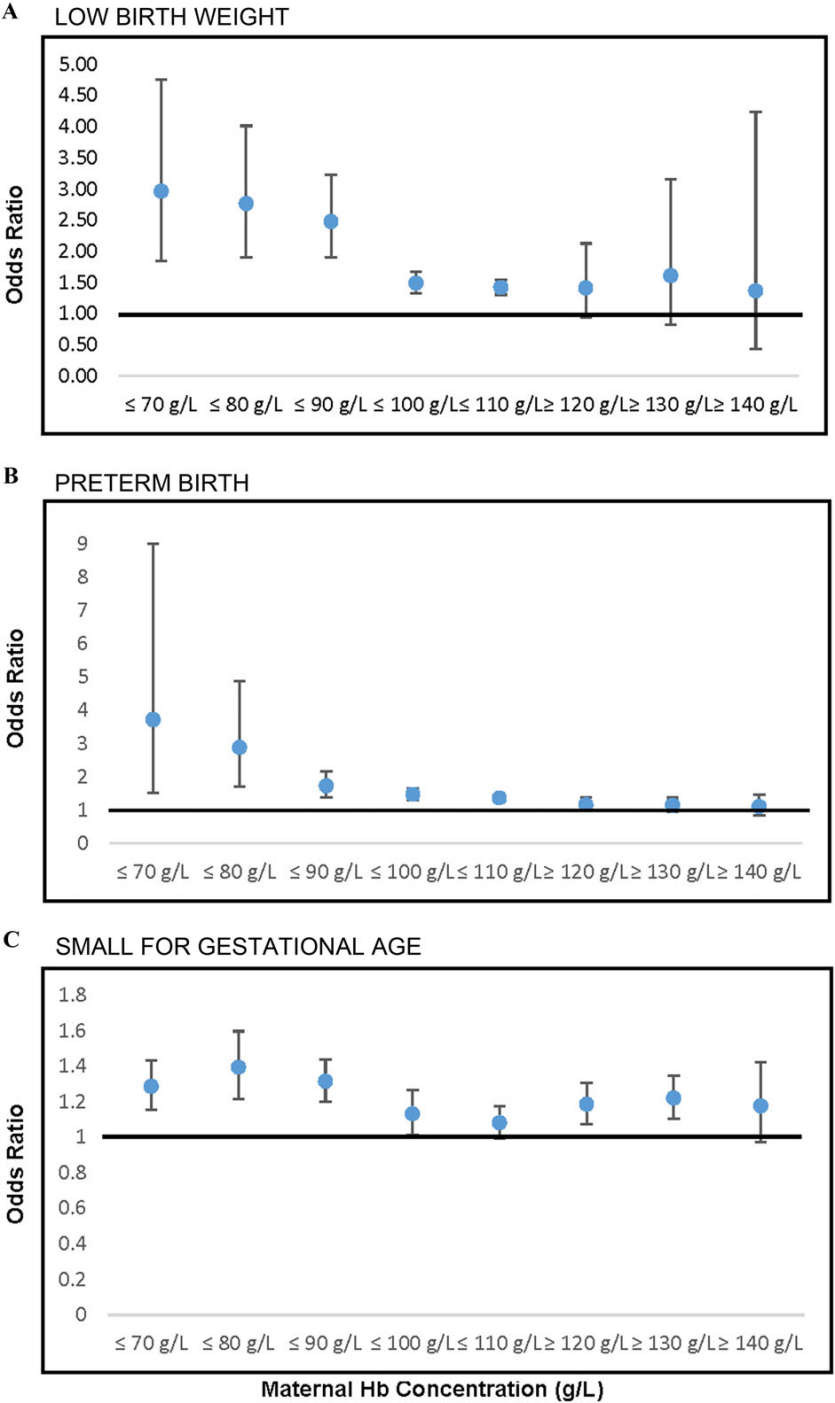
Meta‐analysis summary estimates of association of maternal hemoglobin concentration (g/L) measured at any point during pregnancy and LBW, PTB, and SGA by hemoglobin concentration cutoffs.

Summary estimates were constructed by Hb concentration cutoff: ≤70, ≤80, ≤90, ≤100, ≤110, ≥120, ≥130, and ≥140 g/L (Fig. 2A). There was a dose−response relationship between low maternal Hb concentration and the odds of delivering an LBW infant (≤70 g/L: OR (95% CI); 2.97 (1.85−4.75); ≤80 g/L: 2.77 (1.91−4.02); ≤90 g/L: 2.48 (1.90−3.23); ≤100 g/L: 1.49 (1.33−1.67); ≤110 g/L: 1.42 (1.31−1.55)) (Table 1). Point estimates for the odds of LBW associated with elevated Hb concentrations were not statistically significant (≥120 g/L: OR (95% CI); 1.41 (0.94−2.13); ≥130 g/L: 1.61 (0.82−3.16); ≥140 g/L: 1.37 (0.44−4.23); ≥150 g/L: 0.78 (0.19−3.18); ≥160 g/L: 0.78 (0.19−3.18)) (Table 2).

#### Timing

Estimates from studies examining low Hb concentration (any definition: ≤110 g/L or lower) were divided based on the timing of measurement (preconception, first, second, and third trimesters, and overall (during pregnancy)) (Table 1 and Fig. S11, online only). Overall, low Hb concentration measured at any time point was associated with an increased risk of LBW (preconception: OR (95% CI); 1.72 (1.31−2.26); first trimester: OR (95% CI); 1.23 (1.07−1.41); second trimester: OR (95% CI); 1.14 (0.78−1.68); third trimester: OR (95% CI); 1.65 (1.39−1.96)). Only in the second trimester was this relationship not statistically significant. Low Hb concentration during the preconception period had the strongest association with increased odds of LBW, but this estimate was based on limited data (Table 1).

Elevated Hb concentration was not significantly associated with LBW when measured preconception or in the first trimester (Table 2 and Fig. S12, online only). In the second trimester, there were increased odds of LBW associated with high maternal Hb and in the third trimester decreased odds of LBW; however, these results are based on limited data (<3 studies) and should be interpreted with caution.

#### Overall estimate

In a meta‐analysis of all studies during pregnancy (*n* = 36), low maternal Hb was associated with increased odds of LBW (OR (95% CI); 1.42 (1.31−1.55)) (Table 1). High maternal Hb (*n* = 5) was not significantly associated with LBW, though there was a similar trend (OR (95% CI); 1.80 (0.86−3.77)) (Table 2).

### Preterm birth

#### Hemoglobin cutoff

Overall, low Hb concentration was significantly associated with higher odds of PTB, with larger odds ratios seen with lower cutoffs (≤70 g/L: OR (95% CI); 3.72 (1.53−9.03); ≤80 g/L: 2.89 (1.71−4.89); ≤90 g/L: 1.73 (1.38−2.16); ≤100 g/L: 1.47 (1.32−1.65); ≤110 g/L: 1.36 (1.26−1.46)) (Fig. 2B and Table 1). None of the odds ratios with regard to high Hb concentration were statistically significant (≥120 g/L: OR (95% CI); 1.16 (0.97−1.38); ≥130 g/L: 1.14 (0.95−1.37); ≥140 g/L: 1.10 (0.84−1.46); ≥150 g/L: 1.01 (0.55−1.85); ≥160 g/L: 0.42 (0.14−1.29)) (Fig. 2B and Table 2).

#### Timing

Summary estimates were constructed according to the timing of Hb measurement (Table 1). Results for preconception Hb and odds of PTB were nonsignificant; however, they were in a similar direction as observed during pregnancy, although based on limited data. When studies were divided by trimester of measurement, low Hb concentration in each trimester was associated with increased odds of PTB (first trimester: OR (95% CI); 1.28 (1.17−1.40); second trimester: OR (95% CI); 1.37(1.15−1.63); third trimester: OR (95% CI); 1.45 (1.23−1.71)) (Table 1 and Fig. S13, online only). High Hb concentrations were not significantly associated with PTB, regardless of the timing of measurement (Table 2 and Fig. S14, online only).

#### Overall estimate

When studies including all time points and cutoffs were pooled across pregnancy, low Hb concentration (*n* = 33) was significantly associated with increased odds of PTB (OR (95% CI); 1.36 (1.26−1.46)) but high Hb concentration (*n* = 10) was not (OR (95% CI); 1.17 (0.94−1.45)) (Tables 1 and 2).

### Small for gestational age

#### Hemoglobin cutoff

Summary estimates for the associations with SGA were constructed according to Hb concentration cutoffs (Fig. 2C, and Tables 1 and 2). The strongest relationships were evident at the lowest Hb concentration cutoffs of ≤80 g/L (OR (95% CI); 1.39 (1.22−1.60)) and ≤90 g/L (1.32 (1.20−1.44)). More modest associations were observed with cutoffs of ≤100 g/L (1.13 (1.01−1.27)) and ≤110 g/L (1.08 (1.00−1.18)). Data were limited for higher cutoffs of Hb concentration and are presented in Table 2 **;** in brief, high Hb concentrations were also associated with increased odds of SGA (OR (95% CI): ≥130 g/L: 1.22 (1.10−1.35)). Results for higher cutoffs (≥140−≥160 g/L) were nonsignificant.

#### Timing

Tables 1 and 2 summarize the results regarding the timing of measurement of maternal Hb concentration, with respect to SGA. Overall, preconception low Hb concentration was associated with increased odds of SGA (OR (95% CI); 1.79 (1.39−2.31)) and high Hb concentration was not (1.03 (0.97−1.10)); however, data were very limited. When divided by the trimester of pregnancy, low maternal Hb concentration was not significantly associated with SGA in any trimester (see Fig. S15, online only). High maternal Hb concentration was not associated with increased odds of SGA in the first or third trimester, but limited data from two studies suggest that high maternal Hb concentration in the second trimester was associated with increased odds of SGA (OR (95% CI); 1.54 (1.31−1.81)) (see Fig. S16, online only).

#### Overall estimate

Overall, both low (*n* = 21) and high (*n* = 4) maternal Hb concentrations were associated with increased odds of SGA (OR (95% CI); 1.08 (1.00−1.18) and 1.22 (1.08−1.37), respectively).

### Stillbirth

#### Hemoglobin cutoff

**Figure 3:**
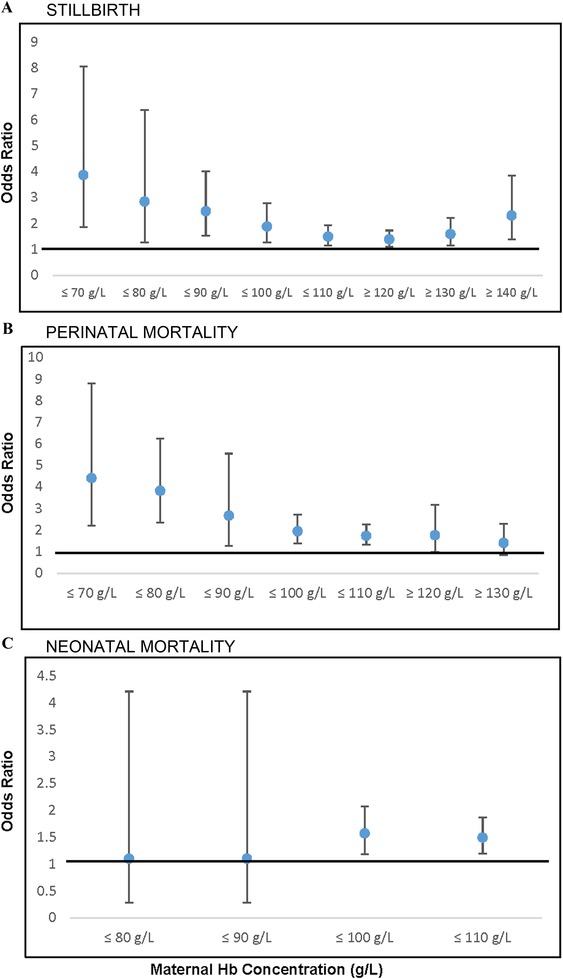
Meta‐analysis summary estimates of association of maternal hemoglobin concentration (g/L) measured at any point during pregnancy and stillbirth, perinatal mortality, and neonatal mortality by hemoglobin concentration cutoffs.

The relationship between maternal Hb cutoff and stillbirth is summarized in Figure 3A, and Tables 1 and 2. A dose response was evident, with lower cutoffs being associated with greater odds of stillbirth (≤70 g/L: OR (95% CI); 3.87 (1.88−8.06); ≤80 g/L: 2.84 (1.27−6.37); ≤90 g/L: 2.47 (1.52−4.01); ≤100 g/L: 1.88 (1.27−2.78); ≤110 g/L: 1.49 (1.15−1.92)). High Hb concentrations were also related to elevated odds of stillbirth (≥120 g/L: 1.38 (1.11−1.73); ≥130 g/L: 1.59 (1.14−2.22); ≥140 g/L: 2.30 (1.38−3.85)).

#### Timing

Only one study (Maghsoudlou *et al*.) examined the relationship of preconception Hb to stillbirth, with results indicating no significant associations with low (OR (95% CI); 1.23 (0.39−3.86) or high (0.83, (0.52−1.33)) Hb concentrations.^17^ During pregnancy, low maternal Hb in the second trimester was associated with increased odds of stillbirth (OR (95% CI); 2.22 (1.36−3.65)). Although associations were nonsignificant for the first and third trimesters, there was a similar trend of elevated odds of stillbirth. The study by Maghsoudlou *et al*. examined the association between high maternal Hb measured during the third trimester and stillbirth; results indicated increased odds of stillbirth with high Hb (OR (95% CI); 2.31 (1.30−4.10)).^17^

#### Overall estimate

The relationship between low Hb concentration (*n* = 21) and stillbirth, with all Hb cutoffs and times during pregnancy combined, was significant (OR (95% CI); 1.49 (1.15−1.92)), and the same was true for high Hb (*n* = 4) (OR (95% CI); 1.88 (1.21−2.91)) (Tables 1 and 2).

### Perinatal mortality

#### Hemoglobin cutoff

Relationships between low maternal Hb concentrations and perinatal mortality are summarized in Table 1 and Figure 3B. There were elevated odds of perinatal mortality associated with Hb ≤70 g/L (OR (95% CI); 4.41 (2.21−8.81)), ≤80 g/L (3.82 (2.34−6.24)), ≤90 g/L (2.66 (1.28−5.55)), ≤100 g/L (1.94 (1.38−2.47)), and ≤110 g/L (1.73 (1.32−2.26)). There were insufficient data available to examine associations between high Hb concentrations and perinatal mortality.

#### Timing

There were limited data on the timing of Hb assessment. While trimester‐specific results were nonsignificant, there was a consistent trend for increased perinatal mortality associated with low maternal Hb across pregnancy (Table 1). Insufficient data were available on the relationship between high maternal Hb and perinatal mortality.

#### Overall estimate

When data across time points (including studies in which the timing was not defined) and cutoffs were combined, low maternal Hb (*n* = 11) was associated with increased odds of perinatal mortality (OR (95% CI); 1.73 (1.32−2.26)) (Table 1).

### Neonatal mortality

#### Hemoglobin cutoff

Summary estimates for odds of neonatal mortality by Hb cutoff were constructed (Fig. 3C and Table 1). A Hb ≤100 g/L was associated with increased odds of neonatal mortality (OR (95% CI); 1.57 (1.19−2.08)) as was a Hb ≤110 g/L (1.49 (1.19−1.87)). There were limited data for lower Hb cutoffs or high Hb concentrations.

#### Timing

Only one study that reported neonatal mortality also reported the time of Hb measurement: Thakur *et al*. measured Hb in the first trimester and found a significant association between maternal Hb <100 g/L and neonatal mortality (OR (95% CI); 3.13 (1.76−5.57)) (Table 1).^93^

#### Overall estimate

When all time points and cutoff values were combined, low maternal Hb (*n* = 5) was associated with increased odds of neonatal mortality (OR (95% CI); 1.49 (1.19−1.87)). There were insufficient data to examine the relationship between high maternal Hb and neonatal mortality.

### Anemia etiology

Overall, very few studies distinguished between IDA and non‐IDA in relation to maternal and child functional outcomes. Data were only available for birth outcomes including LBW, preterm birth, and SGA; summary estimates are provided in Table 3. Summary estimates for the association of LBW with IDA and non‐IDA were both positive but not statistically significant.

**Table 3:** Meta‐analysis summary estimates of association of IDA and non‐IDA with birth outcomes

Outcome	IDA OR (95% CI)	Non‐IDA OR (95% CI)
LBW	1.17 (0.95−1.43)	1.43 (0.82−2.50)
SGA	0.77 (0.68−0.87)	1.20 (0.85−1.70)
PTB		1.07 (0.68−1.70)

LBW: IDA: *n* = 2; non‐IDA: *n* = 3; SGA: IDA: *n* = 2; non‐IDA: *n* = 4; PTB: non‐IDA: *n* = 4.

There was an insufficient number of studies that examined IDA in relation to PTB birth to merit a meta‐analysis. Four studies examined anemia and PTB in non‐IDA populations or in populations with a low prevalence of IDA. The pooled estimate showed a nonsignificant relationship between anemia and PTB (OR (95% CI); 1.07 (0.68−1.70)).

Based on two studies examining the association between IDA and SGA,^26^, ^46^ there were decreased odds of SGA with IDA (OR (95% CI); 0.77 (0.68−0.87)). Four studies examined the association between SGA and non‐IDA or anemia in a population with a low prevalence of ID, and the overall estimate showed no significant association (OR (95% CI); 1.20 (0.85−1.70)).

Data on anemia etiology were not available for stillbirth, perinatal mortality, or neonatal mortality.

## Other child health outcomes

There were too few studies (*n* < 3) to conduct meta‐analyses for several child health outcomes of interest: sudden infant death syndrome (*n* = 1), infant mortality (defined as death before 1 year of life) (*n* = 1), allergic sensitization (*n* = 1), and type I diabetes (*n* = 1). For several other child health outcomes of interest, there were ≥3 studies but the available studies had insufficient or poor quality data, so meta‐analyses were not conducted; these included cardiovascular outcomes (*n* = 8), child development (*n* = 6), child morbidities (*n* = 5), and schizophrenia (*n* = 3). Summaries of these studies (including setting, study design, sample size, exposure, the timing of exposure, and effect estimate) are provided in Table S3 (online only).

## Maternal health outcomes

While much of the research examining maternal Hb concentrations has focused on health implications for the infant, there is a growing interest regarding maternal health outcomes. Below, we report on the associations between maternal Hb concentrations and selected maternal health outcomes. We provide meta‐analyses results when possible (postpartum hemorrhage (*n* = 7), transfusion (*n* = 4), preeclampsia (*n* = 9), and gestational diabetes (*n* = 4)) and summarize results in Table 4. In addition, several studies included in our review used statistical methods incompatible with the inclusion in the overall meta‐analyses; these studies are summarized in Table S7 (online only). For several outcomes, there was an insufficient number of studies for several outcomes for meta‐analyses, and only a qualitative summary is provided (see Table S4, online only). A summary of the number of studies included in each of the meta‐analyses is provided in Table S9 (online only).

**Table 4:** Meta‐analyses of association between maternal Hb concentrations and maternal outcomes by Hb concentration cutoffs

	PPH OR (95% CI)	Preeclampsia OR (95% CI)	Transfusion OR (95% CI)	GDM
**Cutoff: maternal Hb at any time during pregnancy by cutoff**				
≤70 g/L	**6.15 (3.86**−**9.79)** *	**2.83 (2.08**−**3.85)**	**43.46 (22.06**−**85.62)** *	
≤80 g/L	**6.15 (3.86**−**9.79)** *	**2.83 (2.08**−**3.85)**	**24.75 (8.15**−**75.15)** *	
≤90 g/L	**4.02 (2.66**−**6.07)**	**2.30 (1.31**−**4.03)**	**13.96 (3.85**−**50.63)** *	
≤100g/L	**2.40 (1.47**−**3.93)**	**1.93 (1.33**−**2.80)**	**7.70 (2.48**−**23.86)**	
≤110 g/L	**1.84 (1.42**−**2.37)**	**1.68 (1.20**−**2.35)**	**6.57 (3.59**−**12.00)**	0.92 (0.73−1.16)*
Overall estimate** <110 g/L	**1.84 (1.42**−**2.37)**	**1.84 (1.31**−**2.59)**	**6.57 (3.59**−**12.00)**	0.92 (0.73−1.16)*
≥120 g/L	0.84 (0.67−1.05)*	**1.64 (1.24**−**2.18)**		**1.93 (1.33**−**2.81)**
≥130 g/L	0.84 (0.67−1.05)*	**1.34 (1.16**−**1.56)** *		**1.71 (1.19**−**2.46)**
≥140 g/L	0.84 (0.67−1.05)*	1.58 (0.88−2.83)*		**2.10 (1.65**−**2.68)** *
Overall estimate** >130 g/L	0.84 (0.67−1.05)*	**1.48 (1.10**−**2.01)**		**2.02 (1.63**−**2.50)**

notes: The sample size for individual studies is provided in Table S2 (online only), and the total number of studies included in each Hb cutoff effect estimate and timing are specified in Table S9 (online only). A number of studies included for overall estimates for low Hb (<110 g/L) are PPH (6), preeclampsia (8), transfusion (4), GDM (2), and for high Hb (>130 g/L) are PPH (1), preeclampsia (3), and GDM (4). Reference groups for individual studies included in the meta‐analysis were highly variable, with some providing a range (e.g., 110−119 g/L) and others a cut off (≥110 g/L), with little consensus on what represents a “healthy” reference value. Reference groups for all studies included in meta‐analyses can be found in Figures S7–S10 (online only). Reference groups for all studies included in meta‐analyses can be found in Figures S1–S6 (online only). Categories are cumulative; for example, the ≤100 g/L category includes studies using the ≤100 g/L cutoff or any subset where all Hb values were ≤100 g/L. Bold values are statically significant with *P* < 0.05.

^*^Summary estimates from meta‐analyses of <3 studies. Note, for PPH outcome, one study contributes data to the high Hb cutoffs and overall estimate and one study contributes to the lowest Hb cutoffs (≤70 and ≤80 g/L).

^**^Estimates using Hb concentrations measured at any point during pregnancy.

PPH, postpartum hemorrhage; GDM, gestational diabetes mellitus.

### Postpartum hemorrhage

A total of six studies examined the association of maternal hemorrhage with anemia and one study looked at maternal hemorrhage in relation to high Hb concentrations.

#### Hemoglobin cutoff

Summary estimates were constructed to examine the association between postpartum hemorrhage and Hb using different cutoffs (Table 4). Low Hb concentration was significantly associated with postpartum hemorrhage, with the greatest odds with the lowest cutoffs (≤70 g/L: OR (95% CI); 6.15 (3.86−9.79)) compared to higher cutoffs (≤110 g/L: 1.84 (1.42−2.37)). One study examined the association between high Hb and postpartum hemorrhage and reported no significant association (OR (95% CI); 0.84 (0.67−1.05)).

#### Timing

Only two studies specified the time of measurement of Hb when examining the association with postpartum hemorrhage. Both studies measured Hb in the third trimester and overall found higher odds of postpartum hemorrhage with low maternal Hb (OR (95% CI); 1.39 (1.12−1.72)).

#### Overall estimate

When all time points and cutoffs were combined, low Hb (*n* = 6) was associated with increased odds of postpartum hemorrhage (OR (95% CI); 1.84 (1.42−2.37)). There were insufficient data for the association between high Hb and postpartum hemorrhage.

### Transfusion

#### Hemoglobin cutoff

Four studies were included on transfusion, an indicator of severe blood loss at delivery, and low maternal Hb. Associations are reported for cutoffs ≤100 g/L (OR (95% CI); 7.70 (2.48−23.86)) and ≤110 g/L (6.57 (3.59−12.00)) (Table 4). Estimates for lower cutoffs were substantially higher (≤70 g/L: OR (95% CI); 43.46 (22.06−85.62)) but based on limited data (<3 studies).

#### Timing

Data to examine the timing of Hb measurement were not available.

#### Overall estimate

Overall, across pregnancy low Hb (*n* = 4) was associated with increased odds of receiving a blood transfusion (OR (95% CI); 6.57 (3.59−12.00)) (Table 4).

### Preeclampsia

#### Hemoglobin cutoff

Low Hb was significantly associated with higher odds of preeclampsia (≤70 g/L: OR (95% CI); 2.83 (2.08−3.85), ≤110 g/L: OR (95% CI); 1.68 (1.20−2.35)) (Table 4). High Hb concentrations were also associated with increased odds of preeclampsia (≥120 g/L: OR (95% CI); 1.64 (1.24−2.18), ≥130 g/L: 1.34 (1.16−1.56)).

#### Timing

Data to examine the timing of Hb measurement were not available.

#### Overall estimate

Overall, low Hb (*n* = 8) was significantly associated with higher odds of preeclampsia (OR (95% CI); 1.84 (1.31−2.59)). High Hb (*n* = 3) was also associated with higher odds for preeclampsia (OR, 95% CI; 1.48 (1.10−2.01)) (Table 4).

### Gestational diabetes

#### Hb cutoff

There are limited data examining the association between Hb concentrations and gestational diabetes. There is a nonsignificant trend for decreased odds of gestational diabetes associated with low Hb (≤110 g/L OR (95% CI); 0.92 (0.73−1.16)), but based on limited data (<3 studies). On the other hand, high Hb concentrations were associated with increased odds of gestational diabetes (≥120 g/L OR (95% CI); 1.93 (1.33−2.81); ≥130 g/L; 1.71 (1.19−2.46); ≥140 g/L; 2.10 (1.65−2.68)).

#### Timing

Data to examine the timing of Hb measurement were not available.

#### Overall estimate

When all time points and cutoff values were combined, low Hb (*n* = 2) was not significantly associated with increased odds of gestational diabetes, but high Hb (*n* = 3) was associated with elevated odds (OR (95% CI); 2.02 (1.63−2.50)) (Table 4).

### Anemia etiology

Data to examine the etiology of anemia were not available for postpartum hemorrhage, preeclampsia, or transfusion.

## Other maternal health outcomes

There were a number of maternal health outcomes of interest for which our review did not find enough studies to conduct a meta‐analysis. Outcomes included eclampsia (*n* = 2), infection (*n* = 5), prenatal depression (*n* = 2), postpartum depression (*n* = 5), other maternal morbidities (*n* = 3), and maternal mortality (*n* = 5); summaries of these studies (including setting, study design, sample size, exposure, timing of exposure, and effect estimate) may be found in Table S4 (online only).

### Eclampsia

Two studies reported nonsignificant associations between low Hb and eclampsia.^31^, ^83^ Insufficient data were available to conduct meta‐analyses.

### Maternal mortality

The association between maternal mortality and maternal Hb was reviewed in the 2013 *Lancet* series on maternal and child nutrition.^2^ The *Lancet* series reported that, based on a meta‐analysis of 10 studies, it is estimated that a 10 g/L greater Hb is associated with lower odds of maternal mortality (OR (95% CI); 0.71 (0.60−0.85)). In our review of the literature, we found five additional articles published since the *Lancet* series, with three of the articles presenting adjusted analyses. In a Peruvian cohort of 379,816 women, Gonzales *et al*. reported increased odds of maternal mortality for women with Hb concentrations <70 g/L: OR (95% CI); 8.54 (2.06−35.42), 70−<90 g/L: 6.06 (3.16−11.6), and >145 g/L: 2.01 (1.12−3.61), as compared with women with Hb concentrations of 110−145 g/L.^54^ Hb was measured during the second or third trimester and there was no significant association with maternal mortality for women with mild anemia (≤110 g/L). In a Scottish cohort of 80,422 singleton pregnancies with Hb concentration measured at booking (any time before birth), Rukuni *et al*. found no significant association between maternal anemia (≤100 g/L) and maternal mortality (OR (95% CI); 2.07 (0.22−9.36)).^83^ Most recently, Daru *et al*. concluded that severe anemia (<70 g/L) during pregnancy or postpartum doubled the odds of maternal death.^122^ However, the timing of measurement is unclear and this estimate may be an underestimation of the true effect.^123^ Details for these studies and the two studies presenting unadjusted analyses are available in Table S4 (online only).

### Depression

Maternal depression, both during pregnancy and postpartum, is a common condition which can also affect the health and development of infants. Seven studies in our review included maternal depression as an outcome. We were unable to perform a meta‐analysis due to the variation in the presentation of the prenatal or postpartum depression outcome. Recent research conducted in India and Ethiopia indicates an increased likelihood of prenatal depression among anemic women.^124^, ^125^ A cross‐sectional study in Turkey of pregnant women in their last trimester reported that anemic women were more likely to have a higher mean depression score than nonanemic women.^126^ Goshtasebi *et al*. found that women with anemia at delivery were more likely to have postpartum depression (OR (95% CI); 4.64 (1.33−16.08)).^127^ Raisanen *et al*. and Xu *et al*. likewise reported that women with anemia during pregnancy were more likely to be diagnosed with depression.^128^, ^129^ However, a cross‐sectional study in India of pregnant women in their first trimester reported that depressive symptoms were less likely to be present in anemic women compared with nonanemic women.^130^ Details of these studies are included in Table S4 (online only). Overall, the majority of the research tends to show that maternal anemia is associated with depression and depressive symptoms.

### Other maternal morbidities

Three studies examined other maternal morbidities, of which two studies reported adjusted results. One study examined severe maternal morbidity directly attributed to antenatal pulmonary embolism, eclampsia, acute fatty liver of pregnancy, amniotic fluid embolism, peripartum hysterectomy, stroke in pregnancy, uterine rupture, placenta accreta, HELLP (hemolysis, elevated liver enzymes, low platelet count) syndrome, or severe sepsis. A trend for higher odds of maternal morbidity was seen for women with Hb concentration <90 g/L during pregnancy compared with women with Hb concentration ≥90 g/L (OR (95% CI); 1.80 (0.99−3.26)).^131^ A second study reported on a composite adverse maternal outcome defined as preeclampsia, GDM, preterm birth, PPH, puerperal morbidity, or surgical wound complication. Women with Hb concentration ≤110 g/L during pregnancy had higher odds for this composite outcome than nonanemic women 3.03 (1.14−8.05).^132^

## High‐risk populations

Our review also examined the associations between maternal Hb concentration and maternal and child outcomes of interest in populations that are typically at higher odds of adverse outcomes for both mother and child. This included women with HIV, young adolescents (<15 years), women with preeclampsia, twin pregnancies, and other high‐risk health conditions (Table S5, online only). Eleven studies conducted in women with HIV examined the association between Hb concentration and maternal mortality (*n* = 3), child mortality (*n* = 2), preterm birth (*n* = 2), SGA (*n* = 1), preeclampsia (*n* = 1), hemorrhage and transfusion (*n* = 1), and asymptomatic bacteriuria (*n* = 1). Five studies evaluated the relationship of maternal Hb to birth weight (*n* = 5), preterm birth (*n* = 2), stillbirth (*n* = 1), and SGA (*n* = 1) among young adolescents. Two studies in women with preeclampsia examined maternal mortality and LBW. Our review found two studies that examined the odds of various maternal and child outcomes in twin pregnancies. Overall, additional research is needed to appropriately assess the role of maternal Hb concentrations during pregnancy with regard to odds of adverse maternal and child outcomes among these high‐risk populations.

## Discussion

Our results confirm that maternal Hb plays an important role with regard to maternal and child health outcomes.^6^, ^7^, ^8^, ^9^, ^10^, ^11^, ^12^ Low maternal Hb was associated with increased odds of poor birth outcomes including LBW, preterm birth, SGA, stillbirth, perinatal mortality, and neonatal mortality, and adverse maternal health outcomes, including postpartum hemorrhage, preeclampsia, and transfusion. High maternal Hb was likewise associated with increased odds of poor birth outcomes (SGA and stillbirth) and adverse maternal health outcomes (preeclampsia and gestational diabetes).

There was high heterogeneity among the studies included in the review regarding definitions of anemia, cutoffs used for low or high Hb concentrations, and reference values used for comparison. While this represents a challenge in interpreting the overall results, it offered an opportunity to conduct meta‐analyses comparing different cutoffs. For LBW, PTB, stillbirth, and perinatal mortality, there was a dose−response relationship, with associations with low Hb being nearly two times stronger when based on the lowest cutoffs (≤70 or 80 g/L) compared with the overall estimate (<110 g/L). Similarly for adverse maternal outcomes, a dose response was evident for low Hb cutoffs and odds of postpartum hemorrhage, preeclampsia, and blood transfusion. On the other hand, while based on more limited data, there did not appear to be a dose response with elevated cutoffs for high Hb and birth outcomes or adverse maternal health outcomes. The exception was a stillbirth where elevated cutoffs (≥140 g/L) were associated with increased odds of stillbirth compared with the overall estimate for high maternal Hb (>130 g/L).

The associations between maternal Hb and maternal and child outcomes varied by the timing of the assessment. Studies were categorized based on whether maternal Hb was measured during the preconception period, or in the first, second, or third trimester of pregnancy. Our search identified only four papers on maternal preconception Hb and birth outcomes.^17^, ^82^, ^98^, ^111^ Low Hb concentration during preconception was associated with increased odds of LBW and SGA. During the first trimester, low Hb was associated with increased odds of LBW, PTB, and neonatal mortality (results were nonsignificant but followed similar trends for overall estimates for other outcomes). There were limited data on preconception and first trimester high Hb and birth outcomes with many comparisons having fewer than three studies. Results that are specific to Hb preconception and in the first trimester are particularly useful because they may have important implications for the timing and delivery of maternal interventions and prenatal care. There were comparably fewer studies with Hb measured during the second trimester compared to the first or third trimester of pregnancy. Low maternal Hb concentration assessed during the second trimester was not associated with LBW, SGA, or perinatal mortality, which is in contrast to the significant associations reported for overall pregnancy estimates. Conversely, a significant association between high maternal Hb concentration and increased odds of LBW and SGA was found only during the second trimester and results for other trimesters were nonsignificant or in an opposite direction (e.g., LBW in the third trimester). However, data for trimester‐specific comparisons regarding high Hb were limited (<3 studies) and thus this issue merits further research. The second trimester is of particular importance in reviewing the cutoffs for anemia, given changes in blood volume and variation in recommendations for Hb cutoffs during this period. In the third trimester, low maternal Hb was associated with LBW and PTB but not SGA, perinatal mortality, or stillbirth. In contrast, high maternal Hb was associated with greater odds of stillbirth. Further research is needed to confirm these findings and understand the mechanisms that may underlie differential associations based on the timing of Hb assessment.

The key strength of this review and meta‐analyses is the broad scope of health outcomes included for both the mother and child. Additional strengths include separate analyses by Hb concentration cutoff and timing of measurement. There are also a number of limitations of this review. Given the U‐shaped curve of the relationship between maternal Hb and adverse birth outcomes,^5^ we were not able to conduct meta‐analyses using continuous variables for Hb. We were unable to assess the shape of relationships and determine possible points of inflection (e.g., data‐driven cutoffs) as this would require access to individual‐level data. There was also variation in statistical measures used across studies, which limited the studies available for our meta‐analyses. Another limitation in the interpretation of the overall estimates during pregnancy is that studies that provided results using multiple Hb concentration cutoffs were included more than once and thus were more heavily weighted. We chose to include all data and cutoffs available given the goal of providing insight on Hb cutoffs in relation to health outcomes. There was also variation across studies in the reference group used for comparisons. Some studies compared low Hb to a normal range of Hb values, while others compared anemic to nonanemic women. The latter is potentially problematic as the nonanemic category (e.g., Hb ≥ 110 g/L) also includes individuals with high Hb and may bias results to the null. Likewise, for some lower cutoff values for Hb, the comparison group (e.g., Hb ≥ 90 g/L) included individuals with mild/moderate anemia, “healthy values,” and high Hb; which may further attenuate results. In addition, among studies that included a reference range, there was little consensus across studies on the range to use (e.g., 110−119 or 115.3−133.3 or 110−145 g/L). This makes direct comparisons and pooling of results for meta‐analysis difficult; however, we chose to include all data and comparisons since the goal of our review was to provide a landscape of existing literature. Given this limitation, results should be interpreted with caution, as it is possible that they are underestimates of the association of anemia with poor outcomes.

Several research gaps were identified through this review (highlighted in Table 5). There were insufficient data reported on anemia etiology to examine if associations between anemia and maternal and child outcomes vary by the cause of anemia. Given the diverse etiology of anemia across the globe, this is an important research gap. In addition to IDA, anemia due to other causes including malaria, infection, hemoglobinopathies, or other nutrient deficiencies should be examined. In addition, data were insufficient to conduct meta‐analyses on certain key outcomes of interest. In particular, data were limited to maternal health outcomes (e.g., depression) and long‐term child health outcomes (e.g., cardiovascular outcomes, diabetes, and child development). In addition, further research among certain high‐risk populations (young pregnant adolescents and twin pregnancies) would be beneficial in determining if separate cutoffs may be required.

**Table 5:** Key priority areas of research

Key priority areas of research
It remains unclear how the relationship between anemia and maternal and child outcomes may vary based on the cause of anemia and potential implications for anemia cutoffs. Additional research is required to better understand the association of maternal Hb with maternal outcomes (e.g., depression) and long‐term child health outcomes (e.g., cardiovascular outcomes, diabetes, and child development). Few studies report relationships between continuous measures of maternal Hb and health outcomes. Further research is required on the role of maternal preconception Hb and birth outcomes. Few studies provide maternal Hb data during multiple time periods during pregnancy in the same cohort of women. This information is needed to guide potential trimester‐specific cutoff decisions. Limited data are available in high‐risk populations and further research is needed to understand if separate cutoffs are needed (i.e., twin pregnancies). Pooled, high‐quality, individual‐level prospective cohort data are needed to conduct cutoff analysis to inform the reevaluation of Hb cutoffs during pregnancy.

Data on the specific timing of measurement were lacking in many of the studies assessed and there was a particular gap during preconception and the second trimester of pregnancy. Ideally, prospective cohort data with maternal Hb values at multiple time periods across the continuum of preconception, pregnancy, and postpartum would allow us to better assess the role of the timing and guide potential trimester‐specific cutoff decisions. While it was beyond the scope of the current review, the association between postpartum maternal Hb and maternal and child health outcomes may also merit further examination, including a possible relationship between maternal anemia during lactation and breastmilk macronutrient composition.^133^ Furthermore, the majority of the literature that examines the role of the timing on health outcomes has focused on low Hb. Critical gaps remain regarding the importance of the timing of measurement of high Hb concentrations with respect to adverse outcomes. Interpretation of high Hb concentrations is complicated by a lack of data on plasma volume expansion. Failure of maternal plasma volume expansion has been associated with several adverse pregnancy outcomes as well as elevated Hb concentration. For certain outcomes like preeclampsia and gestational diabetes, where plasma volume changes are related to the condition, interpretation of Hb concentrations is more difficult. Future studies to address this gap should include biomarkers of iron status, inflammation, and blood volume expansion to better elucidate the etiology of these relationships.^134^, ^135^

## Conclusions

The goal of this review was to provide a comprehensive overview of the literature on the association of maternal Hb concentration with maternal and child health outcomes. Our findings support the importance of ensuring that maternal Hb remains in a healthy range during pregnancy as both low and high Hb concentrations were associated with poor birth outcomes. However, current data alone are insufficient for determining if revisions are needed to current Hb cutoffs. A number of key gaps in the literature have been identified as research priority areas (Table 5). In particular, pooled high‐quality individual‐level data analyses, as well as prospective cohort studies that measure Hb during preconception and across pregnancy, would be valuable to inform the reevaluation of Hb cutoffs.

## Author contributions

M.F.Y., B.M.O., S.T., R.M., K.G.D., and A.S.W. were involved in study design and protocol development. S.T., M.F.Y., B.M.O., and A.S.W. led data abstraction and analysis. All authors critically reviewed and contributed to the manuscript.

## Statement

This manuscript was presented at the World Health Organization (WHO) technical consultation “Use and Interpretation of Hemoglobin Concentrations for Assessing Anaemia Status in Individuals and Populations,” held in Geneva, Switzerland on November 29−30 and December 1, 2017. This paper is being published individually but will be consolidated with other manuscripts as a special issue of *Annals of the New York Academy of Sciences*, the coordinators of which were Drs. Maria Nieves Garcia‐Casal and Sant‐Rayn Pasricha. The special issue is the responsibility of the editorial staff of *Annals of the New York Academy of Sciences*, who delegated to the coordinators preliminary supervision of both technical conformity to the publishing requirements of *Annals of the New York Academy of Sciences* and general oversight of the scientific merit of each article. The workshop was supported by WHO, the Centers for Disease Control and Prevention (CDC), the United States Agency for International Development (USAID), and the Bill & Melinda Gates Foundation. The authors alone are responsible for the views expressed in this paper; they do not necessarily represent the views, decisions, or policies of the WHO. The opinions expressed in this publication are those of the authors and are not attributable to the sponsors, publisher, or editorial staff of *Annals of the New York Academy of Sciences*.

## Competing interests

The authors declare no competing interests.

## Supporting information


**Table S1**. Search strategy.
**Table S2**. Summary of all observational studies included in meta‐analysis.
**Table S3**. Summary of other child health outcomes (insufficient data for meta‐analysis).
**Table S4**. Summary of other maternal health outcomes (insufficient data for meta‐analysis).
**Table S5**. Summary of studies in high‐risk populations.
**Table S6**. Summary of child health outcomes with other statistical measures.
**Table S7**. Summary of maternal health outcomes studies with other statistical measures.
**Table S8**. Number of studies assessing maternal hemoglobin concentrations and birth outcomes available for meta‐analysis by hemoglobin concentration cutoff.
**Table S9**. Number of studies assessing maternal hemoglobin concentrations and maternal outcomes available for meta‐analysis by hemoglobin concentration cutoff.
**Figure S1**. Overall meta‐analysis for association between maternal hemoglobin concentration (low: <110 g/L; high: >130 g/L) measured at any point during pregnancy and low birth weight (<2500 g).
**Figure S2**. Overall meta‐analysis for association between maternal hemoglobin concentration (low: <110 g/L; high: >130 g/L) measured at any point during pregnancy and preterm birth (<37 completed weeks gestation).
**Figure S3**. Overall meta‐analysis for association between maternal hemoglobin concentration (low: <110 g/L; high: >130 g/L) measured at any point during pregnancy and small for gestational age (birth weight <10th centile for gestational age).
**Figure S4**. Overall meta‐analysis for association between maternal hemoglobin concentration (low: <110 g/L; high: >130 g/L) measured at any point during pregnancy and stillbirth.
**Figure S5**. Overall meta‐analysis for association between low maternal hemoglobin concentration (<110 g/L) measured at any point during pregnancy and perinatal mortality.
**Figure S6**. Overall meta‐analysis for association between low maternal hemoglobin concentration (<110 g/L) measured at any point during pregnancy and neonatal mortality.
**Figure S7**. Overall meta‐analysis for association between low maternal hemoglobin concentration (<110 g/L) measured at any point during pregnancy and post‐partum hemorrhage.
**Figure S8**. Overall meta‐analysis for association between low maternal hemoglobin concentration (<110 g/L) measured at any point during pregnancy and transfusion.
**Figure S9**. Overall meta‐analysis for association between maternal hemoglobin concentration (low: <110 g/L; high: >130 g/L) measured at any point during pregnancy and pre‐eclampsia.
**Figure S10**. Overall meta‐analysis for association between high maternal hemoglobin concentration (>130 g/L) measured at any point during pregnancy and gestational diabetes.
**Figure S11**. Meta‐analysis summary estimates for association between low maternal hemoglobin (<110 g/L) and low birth weight (<2500 g) by timing of hemoglobin measurement during pregnancy.
**Figure S12**. Meta‐analysis summary estimates for association between high maternal hemoglobin (>130 g/L) and low birth weight (<2500 g) by timing of hemoglobin measurement during pregnancy.
**Figure S13**. Meta‐analysis summary estimates for association between low maternal hemoglobin (<110 g/L) and preterm birth (<37 completed weeks gestation) by timing of hemoglobin measurement during pregnancy.
**Figure S14**. Meta‐analysis summary estimates for association between high maternal hemoglobin (>130 g/L) and preterm birth (<37 completed weeks gestation) by timing of hemoglobin measurement during pregnancy.
**Figure S15**. Meta‐analysis summary estimates for association between low maternal hemoglobin (<110 g/L) and small for gestational age (birth weight <10th centile for gestational age) by timing of hemoglobin measurement during pregnancy.
**Figure S16**. Meta‐analysis summary estimates for association between high maternal hemoglobin (>130 g/L) and small for gestational age (birth weight <10th centile for gestational age) by timing of hemoglobin measurement during pregnancy.
