# Supplementary material for: Maternal hemoglobin concentrations across pregnancy and maternal and child health: a systematic review and meta‐analysis
Source: Ann N Y Acad Sci. 2019 Apr 17;1450(1):47–68. doi: 10.1111/nyas.14093 (PMC6767572; doi:10.1111/nyas.14093)
Supplement: Supplementary file 1 — Table S1. Search strategy. Table S2. Summary of all observational studies included in meta‐analysis. Table S3. Summary of other child health outcomes (insufficient data for meta‐analysis). Table S4. Summary of other maternal health outcomes (insufficient data for meta‐analysis). Table S5. Summary of studies in high‐risk populations. Table S6. Summary of child health outcomes with other statistical measures. Table S7. Summary of maternal health outcomes studies with other statistical measures. Table S8. Number of studies assessing maternal hemoglobin concentrations and birth outcomes available for meta‐analysis by hemoglobin concentration cutoff. Table S9. Number of studies assessing maternal hemoglobin concentrations and maternal outcomes available for meta‐analysis by hemoglobin concentration cutoff. Figure S1. Overall meta‐analysis for association between maternal hemoglobin concentration (low: <110 g/L; high: >130 g/L) measured at any point during pregnancy and low birth weight (<2500 g). Figure S2. Overall meta‐analysis for association between maternal hemoglobin concentration (low: <110 g/L; high: >130 g/L) measured at any point during pregnancy and preterm birth (<37 completed weeks gestation). Figure S3. Overall meta‐analysis for association between maternal hemoglobin concentration (low: <110 g/L; high: >130 g/L) measured at any point during pregnancy and small for gestational age (birth weight <10th centile for gestational age). Figure S4. Overall meta‐analysis for association between maternal hemoglobin concentration (low: <110 g/L; high: >130 g/L) measured at any point during pregnancy and stillbirth. Figure S5. Overall meta‐analysis for association between low maternal hemoglobin concentration (<110 g/L) measured at any point during pregnancy and perinatal mortality. Figure S6. Overall meta‐analysis for association between low maternal hemoglobin concentration (<110 g/L) measured at any point during pregnancy and neonatal mortality. Figure S7. Overall me [file NYAS-1450-47-s001.docx]

OSM Table S1: Search Strategy

| **Date of search** | **October 2018** |
| --- | --- |
| **Databases included** | **PubMed and Cochrane review** |
| **Search terms** | (hemoglobin OR hemoglobin OR anemia OR anaemia) AND  (pregnancy OR pre-conception OR pre-pregnancy OR prepregnancy OR preconception OR maternal) AND  (stunt* OR wast* OR underweight OR weight OR height OR length OR arm circumference OR growth OR growth velocity OR Bayley OR PPVT OR language OR cognitive OR socio-emotional OR mental development OR motor development OR psychomotor OR sensorimotor OR intelligence OR IQ OR executive function OR memory OR attention OR Learning[MeSH] OR Mental Processes[MeSH] OR information processing OR literacy OR reading OR math OR school readiness OR emotion OR event-related potential OR Evoked Potentials[MeSH] OR electroencephalogram OR auditory brainstem response OR chronic disease OR diabetes OR hypertension OR cardio-vascular OR cardiovascular OR mortality OR hospitalization OR infection OR transfusion OR wellbeing OR depression OR Birth Weight[MeSH] OR weight OR length OR Gestational Age[MeSH] OR preterm OR weight for gestational age OR birth outcomes OR Infant, Low Birth Weight[MeSH] OR Infant, Premature[MeSH]) AND  (observational OR cross-sectional OR cross-section OR longitudinal OR trial OR intervention OR programme OR program OR cohort OR prospective *retrospective OR case-control*)  NOT (animals[mh] NOT humans[mh])  NOT (Editorial[pt] OR Letter[pt] OR Case Reports[pt] OR Comment[pt]) |
| **Restrictions** | No restrictions for language or year |

| **OSM Table S2. Summary of all Observational Studies Included in Meta-Analysis** | | | | | | | |
| --- | --- | --- | --- | --- | --- | --- | --- |
| **Study (Author, Year)** | **Setting** | **Study Design** | **Sample Size** | **Exposure** | **Timing of Exposure** | **Maternal and Child Outcomes** | **Covariates** |
| Abeysena, 2010^1^ | Sri Lanka | Prospective cohort | 817 | Anemia: Hb < 104 g/L High Hb: > 139 g/L | 1st trimester | LBW, PTB, SGA, GDM | Past history of abortion, past history of LBW, Rate of weight gain (kg/week) |
| Adam, 2013^2^ | Sudan | Case control | 3,290 | Anemia: Hb < 110 g/L | Predelivery | Preeclampsia | Age group in years, parity groups, educational level, prenatal care, placenta previa |
| Adams, 1995^3^ | United States | Retrospective cohort | 1,825 | Anemia: Hb < 110 g/L | First recorded measure | PTB | Medical center |
| Ali, 2011^4^ | Sudan | Case control | 9,578 | Mild/moderate anemia: 70- 109 g/L Severe anemia: < 70 g/L | Predelivery | PTB, LBW, stillbirth | Age, parity, education, residence, and antenatal care |
| Alwan, 2015^5^ | United Kingdom | Retrospective cohort | 362 | Anemia: ≤ 20 weeks: <110 g/L >20 weeks: < 105 g/L | 1st trimester | SGA, PTB | Maternal age, smoking, gestational diabetes, pre-eclampsia, and area deprivation score |
| Bader, 2010^6^ | Sudan | Case control | 312 | Anemia: < 110 g/L | Predelivery | stillbirth | Age, primipara, housewife, education < secondary level, history of miscarriage, history of stillbirth, lack of antenatal care, male neonates, history of malaria, BMI |
| Baig, 2013^7^ | Pakistan | Case control | 600 | Anemia: < 100 g/L High Hb: >140 g/L | During pregnancy | PTB | Maternal age, diet pattern |
| Banhidy, 2011^8^ | Hungary | Case control | 60,994 | Anemia: < 110 g/L | 1st trimester | PTB, LBW | Maternal age, birth order, and maternal socio-economic status |
| Bian, 2013^9^ | China | Retrospective cohort | 55,633 | Anemia: < 100 g/L | 1st trimester | LBW | Neonate gender, maternal age |
| Bilano, 2014^10^ | Multi-country (24) ^1^ | Cross sectional | 276,388 | Severe anemia: Hb < 70 g/L | During pregnancy | preeclampsia | Maternal age, marital status, education, BMI, parity, history of chronic hypertension, gestational diabetes, cardiac/ renal disease, pyelonephritis/ urinary tract infection, antenatal care visits, facility capacity, GNI per capita, maternal mortality ratio |
| Bodeau-Livinec, 2011^11^ | Benin | Retrospective cohort | 1,508 | Anemia: Hb < 110 g/L Severe anemia: Hb < 80 g/L | 2nd trimester 3rd trimester Predelivery | LBW | Malaria at time of Hb assessment, gravidity, BMI, having latrines, having electricity, level of education, and first anenatal care at time of inclusion for Hb during 2nd trimester or number of ANC visits for Hb during the third trimester or at delivery |
| Borah, 2016^12^ | India | Cross sectional | 450 | Anemia: < 110 g/L | 3rd trimester | LBW | Age, parity and interpregnancy intervals |
| Butwick, 2017^13^ | United States | Case control | 850 | Anemia: ≤ 99 g/L, 100- 109 g/L | Predelivery | PPH | Maternal age, insurance, race/ethnicity, chronic hypertension, gestational age (wk), number of earlier CDs, type of pregnancy, GDM, placenta previa, previous D&C or D&E, Time of CD, Mode of anesthesia, Uterine incision |
| Chen, 2017^14^ | China | Retrospective cohort | 1,174 | Anemia: Hb < 110 g/L | Early pregnancy Late pregnancy | SGA | Maternal age, educational level, household registration, occupation, number of pregnancy, gestational weeks, infant gender |
| Chen, 2018^15^ | Multi-country (16)^2^ | Retrospective cohort | 214,067 | Severe anemia: < 7 g/dL | During pregnancy | Preeclampsia | Age, gravidity, marital status, education, cardiac or renal disease, and country |
| Chumak, 2010^16^ | Russia | Retrospective cohort | 24,525 | Anemia: < 120 g/L | During pregnancy | stillbirth, PTB, BW | Maternal age, parity, marital status, alcohol consumption, tobacco smoking, and time periods |
| Chumak, 2011^17^ | Russia | Retrospective cohort | 24,526 | Anemia: <90 g/L, 90-99 g/L, 100-109 g/L, 110-119 g/L | During pregnancy | stillbirth, PTB, BW | Maternal age, parity, marital status, alcohol consumption, tobacco smoking, and time periods |
| Cung, 2014^18^ | Palestine | Retrospective cohort | 5,644 | Low Hb: <70 g/L, 70-89 g/L, 90-109 g/L High Hb: ≥145 g/L | Predelivery | stillbirth | Gestational age, birth weight |
| Delpisheh, 2008^19^ | United Kingdom | Case control | 270 | Anemia: < 110 g/L | first ANC visit | IUGR^3^ | Gestational age, underweight, and primiparity |
| Domple, 2016^20^ | India | Case control | 320 | Low Hb: < 80 g/L | During pregnancy | LBW | Weight gain, gestational age, first ANC visit, education of case, education of husband, bad obstetric history, IFA tablets consumption, planned/unplanned pregnancy or underlying disease |
| Drukker, 2015^21^ | Jerusalem | Retrospective cohort | 75,660 | Mild anemia: 10.0- 10.9 g/dL Moderate/severe anemia: <10.0 g/dL | predelivery | LBW, SGA, LGA, PTB, transfusion, PPH | Maternal age at delivery, maternal education as a proxy for SES and income, nullipara, multipara (2-5), or grandmultipara, previous spontaneous abortions, previous csection, assisted reproductive techniques, neonatal gestational age, induction of labor, epidural analgesia, and birthweight percentile |
| Ehrenthal, 2012^22^ | United States | Retrospective cohort | 59,282 | <105 g/L | Predelivery | Transfusion | Demographic, medical, and pregnancy characteristics, gestational age, and birth weight |
| Elhassan, 2010^23^ | Sudan | Case control | 194 | Anemia: < 110 g/L | predelivery | LBW | Maternal age, primiparous status, education < secondary, lack of antenatal care, interpregnancy interval, maternal weight, maternal height, maternal BMI |
| Eng, 2016^24^ | Australia | Case control | 164 | High Hb: ≥145 g/L | Hb at booking | stillbirth | Maternal age at delivery, advanced maternal age, higher BMI, australian by birth, sex of stillbirth, previous stillbirth, previous preterm delivery, previous c-section, primiparity, married, reduced foetal movement in last 2 weeks |
| Ferdous, 2012^25^ | Bangladesh | Prospective cohort | 1,037 | Moderate/severe anemia: < 90 g/L | During pregnancy | Perinatal mortality | Age, parity, SES, education |
| Gaillard, 2014^26^ | Netherlands | Prospective cohort | 7,317 | High Hb: ≥ 132 g/L; Anemia ≤ 11 | 2nd trimester | PTB, SGA, LBW, preeclampsia | Gestational age at enrollment and at blood sampling, maternal age, BMI, parity, ethnicity, education, alcohol consumption during pregnancy, smoking during pregnancy, folic acid supplement use, and multivitamin use |
| GaneshKumar, 2010^27^ | India | Case control | 450 | Anemia: < 110 g/L | During pregnancy | LBW | Age, parity, antenatal visits, spacing, weight, height, PIH, h/o abortion, stillbirth |
| Geelhoed, 2006^28^ | Ghana | Prospective cohort | 309 | Severe anemia: <80 g/L | lowest Hb during pregnancy, predelivery | LBW, perinatal mortality | Maternal age |
| Getiye, 2017^29^ | Ethiopia | Case control study | 1,113 | Anemia: < 11 g/dL | During pregnancy | Perinatal mortality | Educational status, birth interval, ANC follow up, TT vaccination, gestational age, mode of delivery, history of ENND, obstetric complications, fetal presentation, newborn weight, congenital anomaly, partograph use |
| Gonzales, 2009^30^ | Peru | Retrospective cohort | 35,449 | Anemic: < 90, 90- 109 g/L High Hb: > 129 g/L | First available measurement | PTB, SGA, stillbirth | Maternal age, maternal education, marital status, prior stillbirth or preterm birth, prenatal care, parity, maternal body mass index, placental abruption, and gestational hypertension in current pregnancy |
| Gonzales, 2012^31^ | Peru | Retrospective cohort | 379,816 | Low Hb: < 70, 70- <90, 90- <110 g/L High Hb: >145 g/L | 3rd trimester | PPH, preeclampsia | Age, maternal education level, marital status, body mass index, prenatal care, parity, gestational diabetes mellitus and cardiopathy (current pregnancy), gestational age at which Hb was first measured, and migration if delivery was in a place of different altitude to that in which most of the pregnancy occurred |
| Gonzales, 2014^32^ | Peru | Retrospective cohort | 161,909 | Mild anemia: 90- <110 g/L Moderate anemia: 70- <90 g/L Severe anemia: <70 g/L | 2nd trimester | PTB, stillbirth | Age, BMI, maternal education, antenatal care, parity, preeclampsia, altitude, and urinary infection |
| Hamaleinen, 2003^33^ | Finland | Case control | 22799 | Anemic: < 100 g/L | 1st trimester 2nd trimester 3rd trimester | PTB, LBW, SGA | Adjusted (factors not provided) |
| Hinderaker, 2003^34^ | Tanzania | Prospective cohort | 3,618 | Low Hb: <90, 90-109 g/L High Hb: ≥ 130 g/L | 2nd trimester | Perinatal mortality | Parity, loss of child |
| Hwang, 2010^35^ | South Korea | Retrospective cohort | 3,560 | Anemia: < 100 g/L | 3rd trimester | PTB, SGA, perinatal mortality | Adjusted (factors not provided) |
| Jaleel, 2010^36^ | Pakistan | Case control | 124 | Anemia: <90 g/L | During pregnancy | PPH | Age (> 35 years), previous history of PPH, home delivery |
| Kalanda, 2006^37^ | Malawi | Cross sectional | 1,571 | Low Hb: < 80, < 100, < 110 g/L | 2nd trimester | SGA | Malaria parasitaemia, gestational age, birth weight |
| Kattula, 2014^38^ | India | Prospective cohort | 420 | Anemia: Hb < 100 g/L | 2nd trimester | LBW | Offspring gender, preterm birth, < 4 antenatal care visits, beedi work at home |
| Khan, 2016^39^ | Pakistan | Case control | 160 | Moderate/ severe anemia: < 100 g/L | Predelivery | LBW | Educational level, SES, maternal nutrition, periodontitis |
| Khattar, 2013^40^ | India | Case control | 300 | Severe anemia: < 70 g/L | During pregnancy | LBW | preterm pregnancy, lower SES, previous LBW baby, utilization of ANC, ETS exposure |
| Knottnerus, 1990^41^ | Netherlands | Prospective cohort | 796 | Hb ≥ 8.0 mmol/l | 3rd trimester | LBW, PTB | Pregnancy-induced hypertension |
| Koura, 2012^42^ | Benin | Prospective cohort | 542 | Anemia: < 110 g/L | Predelivery | LBW, PTB | Maternity hospital |
| Lao, 2002^43^ | China | Prospective cohort | 730 | Hb > 130 g/L | First antenatal visit | GDM | Adjusted (factors not provided) |
| Levy, 2005^44^ | Israel | Retrospective cohort | 153,396 | Anemia: < 100 g/L | 1st trimester | PTB, LBW | Ethnicity, maternal age, placental problems, CS, and non-vertex presentation |
| Lone, 2004^45^ | Pakistan | Prospective cohort | 629 | Anemia: < 110 g/L | During pregnancy | Stillbirth, LBW, PTB | Adjusted (factors not provided) |
| Maghsoudlou, 2016^46^ | Iran | Case control | 3,383 | Low Hb: < 110 g/L High Hb: 121- 139, ≥ 140 g/L | Preconception 1st trimester 3rd trimester | stillbirth | Maternal age, pre-pregnancy BMI, maternal height, gestational age at first Hb measurement, parity, smoking status, region of residence, and husband's profession |
| Malhotra, 2002^47^ | India | Prospective cohort | 447 | Low Hb: ≤75, 76-85, 86-95 g/L High Hb: 106-115, ≥ 116 g/L | During pregnancy | LBW | Age, parity |
| Mamun, 2006^48^ | Bangladesh | Prospective cohort | 1584 | Hb: 90-99.9, 100- 109.9, 110- 119.9, ≥ 120 g/L | During pregnancy | Perinatal mortality | Hypertension, antepartum haemorrhage, infection, previous experience of abortions, stillbirth, or newborn death, demographics, socio-economic characteristics, preterm deliveries, maternal age, iron folate supplementation |
| Marchant, 2004^49^ | Tanzania | Cross sectional | 301 | Severe anemia: Hb < 80 g/L | During pregnancy | perinatal mortality | Season of recruitment of the pregnant women, gestational month at recruitment, age, parity, education, marital status, MUAC, BMI |
| Marti, 2001^50^ | Venezuela | Case control | 543 | Anemia: < 110 g/L | 3rd trimester | PTB | Placental abruption, PROM, previous premature labor, prenatal care visits, and uterine Bleeding during more than one trimester |
| Masukume, 2015^51^ | New Zealand, Australia, England, Ireland | Prospective cohort | 5,609 | Anemia: < 110 g/L | 2nd trimester | LBW, PTB, SGA | Country, maternal age, having a marital partner, ethnic origin, years of schooling, and having paid work |
| Meis, 1995^52^ | United Kingdom | Retrospective cohort | 25,844 | Low Hb: < 104, 104- 117 g/L High Hb: ≥ 130 g/L | First antepartum visit | PTB | Age, maternal height, maternal weight, parity, previous abortions, previous stillbirth, maternal smoking, social class, bacteriuria, hypertension-proteinuria, early pregnancy bleeding, late pregnancy bleeding |
| Mohamed, 2012^53^ | United States | Retrospective cohort | 17,338 | Low Hb: < 90, 90- 99, 100-109 g/L High Hb: 120-129, 130-139, ≥ 140 g/L | 3rd trimester | LBW, PTB | Maternal age, body mass index measured during pregnancy and at time of delivery, gravidity, multiple gestation, previous cesarean delivery, thyroid disorder, diabetes mellitus (chronic or gestational), hypertension, street drug use, smoking, alcohol, and infant's sex |
| Mola, 1999^54^ | Papua New Guinea | Retrospective cohort | 21,177 | Low Hb: <80 g/L | 2nd trimester | stillbirth | Syphilis, multiple pregnancy, maternal age > 35 years, highland region of origin, HTN in pregnancy |
| Msuya, 2011^55^ | Tanzania | Prospective cohort | 2,654 | Moderate anemia: 70- 89 g/L Severe anemia: < 70 g/L | 3rd trimester | LBW | HIV, malaria, maternal BMI |
| Mumbare, 2012^56^ | India | Case Control | 2,998 | Anemia: < 110 g/L | 3rd trimester | LBW | Inadequate ANC, weight <55 kg, height <145 cm, tobacco exposure, HTN, low SES, parity 1, maternal education, paternal education, nuclear family, age of mother |
| Nair, 2017^57^ | United Kingdom | Retrospective cohort | 14,001 | Mild anemia: 100-109 g/L  Moderate anemia: < 100 g/L | First visit  Third trimester | Stillbirth, perinatal mortality | Maternal age, body mass index, parity, smoking status, gestational diabetes, antepartum haemorrhage and pregnancy-induced hypertension during index pregnancy, pre-existing diabetes mellitus, haemoglobinopathies, other medical comorbidities and ethnicity |
| Nyflot, 2017^58^ | Norway | Case control | 3,123 | Anemia: ≤ 90 g/L | 1st trimester | PPH | Maternal age, BMI, birth weight |
| Obadi, 2018^59^ | Yemen | Case control | 303 | Anemia: < 110 g/L | During pregnancy | Stillbirth | Maternal age, mother's education, gestational age, prolonged labor, antenatal care visits, unbilical complications, low birth weight, congenital malformation |
| Ota, 2014^60^ | Multi-country (29) ^4^ | Cross sectional | 245,773 | Severe anemia: < 70 g/L | Predelivery | SGA | Maternal age, marital status, education, parity, medical conditions during pregnancy such as chronic hypertension, preeclampsia/eclampsia, malaria/dengue, and HIV/AIDS at the indiv level, and capacity of health facilities at the facility level by four categorized HDI groups |
| Patel, 2018^61^ | India | Prospective cohort | 72,750 | Mild anemia: 10 - 11 g/dL  Moderate: < 10 g/dL | First antenatal visit | Stillbirth, LBW, neonatal mortality | Clustering, mother’s age, education level and parity, along with the three levels of anaemia and three categories of BMI |
| Phaloprakarn, 2008^62^ | Thailand | Retrospective cohort | 874 | High Hb: ≥ 125 g/L | 1st trimester | PTB, LBW, SGA, GDM, preeclampsia | Preeclampsia, GDM |
| Poespoprodjo, 2008^63^ | Indonesia | Cross sectional | 3,046 | Severe anemia: < 70 g/L | Predelivery | LBW | Any parasitemia, Papuan ethnicity, primigravid, prematurity |
| Raisanen, 2014^64^ | Finland | Retrospective cohort | 284,415 | Anemia: < 100 g/L | During pregnancy | PTB, SGA, stillbirth, neonatal mortality, preeclampsia | Maternal age, number of prior births in multiparous women, birthweight, fetal sex, IVF, pre-eclampsia, placenta previa, marital status, smoking status and SES |
| Raisanen, 2013^65^ | Finland | Case control | 1,390,742 | Anemia: ≤ 100 g/L | During pregnancy | PTB* | Maternal age, fetal sex, prior preterm delivery in multiparous women, socio-economic status, smoking, in vitro fertilisation, placenta previa, and placental abruption |
| Ren, 2007^66^ | China | Retrospective cohort | 88,149 | Anemic:  <80, 80-99, <110 g/L High Hb: 120-139, 140-159, ≥ 160 g/L | 1st trimester | LBW, PTB, SGA | Maternal age, education, gravidity, and BMI |
| Ribot, 2014^67^ | Spain | Prospective cohort | 282 | Anemia: < 110 g/L | 1st trimester 2nd trimester 3rd trimester | PTB | Smoking behavior of mother, maternal age, parity, sex of child, BMI at first visit, SES, iron supplementation per day (mg) |
| Ronnenberg, 2004^68^ | China | Prospective cohort | 405 | Low Hb: <95, 95-120 g/L | Preconception | LBW, PTB, FGR^4^ | Maternal age, height and height squared, BMI, education, exposure to dust, noise, and passive smoking, work stress, infant gender, and gestational age, and deficiency of folate, vitamin B12 and B7 |
| Rukuni, 2016^69^ | United Kingdom | Retrospective cohort | 80,422 | Anemia: < 100 g/L | Hb at booking | PPH, transfusion, stillbirth, PTB, LBW, neonatal mortality, preeclampsia | Age, parity, smoking status, ethnicity, socio-economic status, body mass index and chronic kidney disease. |
| Saeed, 2014^70^ | Sudan | Cross sectional | 381 | Moderate/severe anemia: < 90 g/L | Predelivery | LBW | Educational level, type of pregnancy, gestational age, presence of hypertension, renal disease, and bleeding during pregnancy |
| Scanlon, 2000^71^ | United States | Retrospective cohort | 282,123 | 1st trimester: < 97.5, 97.5- 107, 107-116, 134-143, 143-152, >152 g/L  2nd trimester: <89.5, 89.5-98.5, 98.5-107.5, 116.5-134.5, 134.5-143.5, > 143.5 g/L  3rd trimester: <97.3, 97.3- 106.3, 106.3-115.3, 133.3-142.3, 142.3-151.3, >151.3 g/L | 1st trimester 2nd trimester 3rd trimester | PTB, SGA | Maternal race, age, education level, marital status, pre-pregnancy body mass index, weight gain, and cigarette use during pregnancy |
| Schmiegelow, 2012^72^ | Tanzania | Prospective cohort | 872 | Anemia: <80 g/L | Predelivery | Perinatal mortality | Age, gravidity, education <= primary level, short maternal stature, GA at inclusion >21 weeks, adherence to ANV program, full course of IPTp (>= 2 doses), ever used bed net, pregnancy induced HT, preeclampsia, preeclampsia before ANV4, preterm delivery, SGA, antepartum hemorrhage, placental weight, place of delivery |
| Scholl, 1992^73^ | United States | Prospective cohort | 779 | Anemic: 1st/3rd trimester: <110 g/L 2nd trimester: < 105 g/L | 2nd trimester | LBW, PTB, SGA | Maternal age, parity, ethnicity, prior LBW or preterm delivery, bleeding at entry, gestation at initial blood draw (entry), number of cigarettes smoked per day, and pregenancy BMI |
| Scholl, 1994^74^ | United States | Prospective cohort | 755 | Anemia: < 110 g/L | 3rd trimester | LBW | Maternal age, parity, black ethnicity, cigarettes smoked/day, prepregant body mass index, vaginal bleeding before 28 weeks, dietary energy intake, and protein intake |
| Sharma, 2015^75^ | Nepal | Case control | 465 | Anemia: < 110 g/L | During pregnancy | LBW | History of premature delivery, hard physical work done during pregnancy, current age of mother, height of mother, consumig nutritious food during pregnancy, ethnicity, and family type |
| Shehata, 2017^76^ | Canada | Retrospective cohort | 26,994 | Hb range: 0 - 69 g/L, 70 - 79 g/L, 80 - 89 g/L, 90 -99 g/L, >= 100 g/L | During pregnancy | Transfusion | Discharge year, maternal age admission, gestational age, parity, CVD, infection, obesity, placenta previa, preeclampsia, PPH, ICU visit, antepartum hemorrhage |
| Smithers, 2014^77^ | Australia | Retrospective cohort | 96,290 | Anemia: <110 g/L | 1st trimester | LBW,PTB^5^ | Singleton/twin, maternal age, smoking in pregnancy, number of antenatal visits, parity, inter-pregnancy interval, maternal occupation, paternal occupation, maternal Aboriginal or Torres Strait Islander status, the Index of Relative Socio-economic Disadvantage and for living in a remote/not remote area |
| Steer, 1995^78^ | United Kingdom | Retrospective cohort | 153,602 | Anemia: ≤85, 86-95 g/L High Hb: 106- 115, 116-125, 126- 135, 136-145, > 145 g/L | lowest Hb during pregnancy | LBW, PTB | Ethnic group, BMI, parity, maternal age, and smoking |
| Stephansson, 2000^79^ | Sweden | Case control | 1,404 | Low Hb: ≤ 115, 116-125 g/L High Hb: 136-145, ≥ 146 g/L | 1st trimester | stillbirth | Maternal age, height, occupation, smoking, BMI, and week of 1st Hb concentration measurement |
| Tandu-Umba, 2015^80^ | Congo | Cross sectional | 412 | Anemia: < 100 g/L | Predelivery | PTB, SGA, stillbirth | Age < 18 yrs, age ≥ 35 years, single status, previous miscarriage, grand multiparity, diabetes in family, previous prematurity, previous LBW, overweight/obesity, previous cesarean section and previous pre-eclampsia |
| Thakur, 2013^81^ | India | Retrospective cohort | 283 | Anemia: < 100 g/L | 1st trimester | neonatal mortality | Socioeconomic status,malnutrition,antenatal care (adequate/ inadequate), pregnancy induced hypertension,antepartum haemorrhage |
| Tsu, 1993^82^ | Zimbabwe | Case control | 450 | Hb: < 120 g/L | 3rd trimester | PPH | Age, parity, obstetric history, antenatal hospitalization, and facility booked |
| Tzur, 2012^83^ | Israel | Retrospective cohort | 33,888 | Anemia: <100 g/L | 1st trimester | PPH, transfusion, IUGR, PTB, LBW, perinatal mortality | Maternal age, ethnicity, previous PTD, cervical incompetence, hypertensive disorders, IUGR, and labor induction |
| Unger, 2015^84^ | Papua New Guinea | Prospective cohort | 671 | Anemia: < 90 g/L | During pregnancy | SGA | Gravidity, gestational age at fetal weight measurement |
| Verhoeff, 2001^85^ | Malawi | Prospective cohort | 1,423 | Anemia: < 80 g/L | During pregnancy | SGA | Primiparae, less than 4 ANC visits, height < 150 cm, MUAC < 23 cm, malaria at delivery |
| Walker, 2003^86^ | Jamaica | Retrospective cohort | 234 | Low Hb: < 95 g/L | 2nd trimester | LBW | Gestational age, first antenatal visit after 20 weeks, height of mother, weight gain of mother after 20 weeks, consumption of alcohol during pregnancy, young maternal age, hypertension, weight in late pregnancy |
| Wang, 2018^87^ | China | Retrospective cohort | 21,577 | Hb < 110 g/L, 130 g/L ≤ Hb < 150 g/L; Hb ≥ 150 g/L | 1^st^ trimester | Preeclampsia, PTB, GDM | Maternal age, pre-pregnancy BMI, gravidity (< 2, ≥ 2), parity (yes, no), education level (≤ 12, > 12), and gestational age at the time of Hb measurement |
| Xiong, 2003^88^ | China | Retrospective cohort | 14,510 | Anemia: < 100 g/L | 1st trimester 3rd trimester | perinatal mortality, PTB, IUGR, LBW | Hospital stay, maternal age,maternal education, parity, gestational age at the first prenatal visit, BMI at the first visit, hypertensive disorders in pregnancy, vaginal bleeding and prior spontaneous abortion |
| Yatich, 2010^89^ | Ghana | Cross sectional | 746 | Moderate anemia: < 110 g/L Severe anemia: < 80 g/L | Predelivery | stillbirth | Age (per 5 years), single, low serum folate, no SP doses, past induced abortion, past stillbirth, malaria, infection |
| Yi, 2013^90^ | Korea | Retrospective cohort | 70,895 | Anemia: < 120, < 100, 100-119, ≥ 150 g/L | Preconception | PTB, LBW, SGA | Maternal age at delivery, prepregnancy body mass index, parity, education and the result of a health-screening examination |
| Zhang, 1992^91^ | China | Case control | 865 | Anemia: < 105 g/L | During pregnancy | stillbirth | Sex, parity, maternal age |
| Zhang, 1993^92^ | China | Case control | 865 | Anemia: < 80, 80-100 g/L | During pregnancy | neonatal mortality | Sex, gravidity, maternal age, threatened abortion, PIH |
| Zhang, 2018^93^ | China | Prospective cohort | 2,722,274 | Hb range: < 70, 70 - 99, 100- 109, 150-159, 160-169, ≥ 170 g/L | Preconception | PTB | Characteristics of women (age, education, ethnic, occupation, region with GDP per capita), smoking, passive smoking and alcohol drinking status at baseline, history of diseases (diabetes, hypertension and thyroid dysfunction), pre-pregnancy BMI, parity, history of adverse pregnancy outcomes and sex of the child |
| Zhang, 2018^94^ | China | Retrospective cohort | 10,430 | ≤ 119 g/L, 120 - 129 g/L, ≥ 140 g/L | 1^st^ trimester  2^nd^ trimester | PTB | Education, occupation, gestational age at the first visit, BMI, maternal age, and treatment group |
| Zhou, 1998^95^ | China | Prospective cohort | 829 | Hb: < 90, 90-99, 100-109, < 110, 120-129, ≥ 130 g/L | 1st trimester | LBW, PTB, SGA | Maternal age, gravidity, parity, height, weight, BMI, blood pressure, and infant sex |

Hb, hemoglobin; LBW, low birth weight (< 2500 g); PTB, preterm birth (<37 weeks completed gestation); SGA, small for gestational age (birth weight below the 10th centile for gestational age); IUGR, intrauterine growth restriction; BW, birth weight (measured in grams); PPH, post-partum hemorrhage; GDM, gestational diabetes mellitus.
^1^ Multi-country study conducted in Algeria, Angola, Democratic Republic of Congo, Niger, Nigeria, Kenya, Uganda, Argentina, Brazil, Cuba, Ecuador, Mexico, Nicaragua, Paraguay, Peru, Cambodia, China, India, Japan, Nepal, Philippines, Sri Lanka, Thailand, and Vietnam

^2^ Multi-country study conducted in Algeria, Argentina, Brazil, Congo, Cuba, Ecuador, India, Kenya, Mexico, Niger, Nigeria, Peru, the Philippines, Sri Lanka, Thailand, and Uganda
^3^ Study assessed symmetric IUGR only
^4^ Multi-country study conducted in Japan, Qatar, Argentina, Mexico, Lebanon, Peru, Brazil, Ecuador, Sri Lanka, Jordan, China, Thailand, Mongolia, OPT, Paraguay, Philippines, Vietnam, Nicaragua, India, Cambodia, Kenya, Pakistan, Angola, Nigeria, Nepal, Uganda, Afghanistan, DRC, Niger
^4^ FGR defined as birthweight ratio <85%
^5^ Complete case analysis data used in meta-analysis

Referent group defined as greater than the listed cut-offs.

| **OSM Table S3. Summary of Other Child Health Outcomes (insufficient data for meta-analysis)** | | | | | | |
| --- | --- | --- | --- | --- | --- | --- |
| Study (Author, year) | Setting | Study Design | Sample Size | Exposure | Timing of Exposure | Statistical Measure |
| *Birth Weight (g)* |  |  |  |  |  |  |
| Abdel-Raoufabdel-AzizAfifi, 2013^96^ | Egypt | Prospective cohort | 206 | Mild anemia: 80 - 105 g/L Severe anemia: < 80 g/L | During pregnancy | Unadjusted mean, cm (SD): Hb 80- 105 g/L: 51.73 (1.561) Hb < 80 g/L: 50.22 (2.106) |
| Agarwal, 2002^97^ | India | Prospective cohort | 3,700 | Continuous Hb (g/L) | 3rd trimester | Unadjusted difference in means:  < 2,500 g: Hb: 9.8 ± 1.2 g/dL  2500-3000 g: 10.0 ± 1.1 g/dL  > 3000 g: 10.1 ± 1.1 g/dL  𝛽= 20.209 (SE = 20.093) |
| Ali, 2009^98^ | Sudan | Case control | 125 | Anemia: Hb < 110 g/L | Predelivery | Unadjusted means, g (SD):  Anemic: 3122.0 g (446.85) Non-anemic: 3034.0 g (477.92) |
| Alwan, 2015^5^ | United Kingdom | Retrospective cohort | 362 | Anemia: ≤ 20 weeks: <110 g/L >20 weeks: < 105 g/L | 2nd trimester | Adjusted difference in means, g (95% CI):  ≤ 20 weeks gestation: 1.0 (-2.0 – 4.1)  > 20 weeks gestation: -1.2 (-4.4 – 1.4) |
| Baraka, 2012^99^ | Belgium | Cross-sectional | 341 | Anemia: < 11 g/dL | 1^st^ trimester | Unadjusted means, g (SD):  Anemic: 3166 ± 119 g  Non-anemic: 3,442 ± 33 g  p-value = 0.036 |
| Becerra, 1998^100^ | Peru | Cross sectional | 1,015 | Mild anemia: 90- 110 g/L Moderate anemia: 70-90 g/L Severe anemia: < 70 g/L | 3rd trimester | 𝛽= 5.99 (95% CI: -25.71 – 37.69) |
| Caradeux, 2016^101^ | Chile | Prospective cohort | 543 | Continuous Hb (g/L) | 1^st^ trimester | Correlation coefficient: r=0.04 |
| Chumak, 2010^16^ | Russia | Retrospective cohort | 24,525 | Anemia: < 120 g/L | During pregnancy | Difference in mean birthweight (Anemic vs. non-anemic, 95% CI):  48 g (95% CI: 36 – 59) |
| Chumak, 2011^17^ | Russia | Retrospective cohort | 24,526 | Anemia: <90 g/L, 90-99 g/L, 100-109 g/L, 110-119 g/L | During pregnancy | Difference in mean birthweight (95% CI):  <90 g/L: 47 (95% CI: 7 – 91)  90-99 g/L: 73 (95% CI: 49 – 97)  100-109 g/L: 68 (95% CI: 52 – 84)  110-119 g/L: 34 (95% CI: 21 – 47)  > 120 g/L: reference |
| Demmouche, 2011^102^ | Algeria | Prospective cohort | 207 | Mild anemia: 10 ≤ Hb < 11 g/dL  Moderate anemia: 7 ≤ Hb < 10 g/dL  Severe anemia: < 7 g/dL | 3^rd^ trimester | Mean birth weight, g (SD):  Mild anemia: 3,125.71 ± 612.7  Moderate anemia: 3,209.37 ± 78.37  Severe anemia: 3,364.28 ± 141.2 |
| de Sa, 2015^103^ | Brazil | Cross sectional | 54 | Anemia: Hb < 110 g/L | Predelivery | Unadjusted means, g (SD):  Anemic: 3375.9 ± 506.9  Non-anemic: 3300.2 ± 458.4 |
| Dhar, 2003^104^ | Bangladesh | Cross sectional | 316 | Hb < 9 g/dL  9 ≤ Hb < 10 g/dL  10 ≤ Hb < 11 g/dL  11 ≤ Hb < 12 g/dL  ≥ 12 g/dL | During pregnancy | Unadjusted mean, g (SD):  Hb < 9 g/dL: 2,797 (452)  9 ≤ Hb < 10 g/dL: 2,929 (467)  10 ≤ Hb < 11 g/dL: 2,920 (511)  11 ≤ Hb < 12 g/dL: 2,890 (414)  ≥ 12 g/dL: 3,043 (438) |
| Duthie, 1991^105^ | Hong Kong | Case control | 96 | Severe anemia: < 8 g/dL | During pregnancy | Unadjusted mean, g (SD):  Severe anemia: 2,984 (502)  Controls: 3,177 (383)  P < 0.01 |
| Emamghorashi, 2004^106^ | Iran | Cross sectional | 97 | Anemia: Hb < 110 g/L | Predelivery | Unadjusted mean, g:  IDA: 3,517  NAID: 3,211  NC: 3,254 |
| Fareh, 2005^107^ | United Arab Emirates | Case control | 200 | Anemia: ≤11.0 g/dL | 3rd trimester | Unadjusted mean, g (SD):  Cases: 3,158 (53)  Controls: 3,239 (490) |
| Hasin, 1996^108^ | Bangladesh | Cross sectional | 151 | Anemia: < 11.0 g/dL | Predelivery | Adjusted mean Hb, g/dL  BW < 2.5 kg: mean Hb = 12.3 g/dL  BW ≥ 2.5 kg: mean Hb = 12.3 g/dL  p-value = 0.93 |
| Hassan, 2011^109^ | Egypt | Cross sectional | 246 | Continuous Hb | Predelivery | Correlation coefficient: r= -0.11  p-value = 0.03 |
| Jwa, 2015^110^ | Japan | Retrospective cohort | 1,986 | Continuous Hb (g/L) | 1st trimester 2nd/ 3rd trimester | Early pregnancy: 𝛽= -25.10 (95% CI: -43.50 – -6.70)  Mid-pregnancy: 𝛽= -63.40 (95% CI: -82.60 – --44.20)  Late pregnancy: 𝛽= -75.9 (95% CI: -92.7 – -59.10) |
| Lao, 2000^111^ | China | Prospective cohort | 437 | Anemia: < 10 g/dL | 3^rd^ trimester | Unadjusted means, g (SD):  Anemia: 3,082 (416)  No anemia: 3,220 (411)  p-value = 0.035 |
| Laflamme, 2010^112^ | Bolivia | Retrospective cohort | 98 | Anemic: Hb < 110 g/L | 1st trimester | Unadjusted mean, g: Anemic: 3,100  Non-anemic: 3,300  p-value = 0.213 |
| Lee, 2006^113^ | Korea | Prospective cohort | 248 | Continuous Hb (g/L) | Predelivery | 𝛽= -0.687 (SE = 0.287) |
| Lelic, 2014^114^ | Bosnia and Herzegovina | Case control | 100 | Anemia: Hb < 110 g/L | At labor and on 2 previous occasions in current pregnancy | Unadjusted mean, g (SD): Anemic: 3,048 (405.96) Non-anemic: 3,615.6 (319.71)  p-value= <0.0001 |
| Levario-Carrillo, 2003^115^ | Mexico | Cross sectional | 153 | Anemia: Hb < 110 g/L | Predelivery | Unadjusted mean, g (SD): Anemic: 3,294 (534) Non-anemic: 3,270 (402) |
| Luis, 2016^116^ | United Kingdom | Case control | 212 | Anemia: < 8 g/dL | During pregnancy | Unadjusted mean, kg (SD):  Cases: 3.29 (0.53)  Controls: 3.2 (0.56)  p-value= 0.25 |
| Kaur, 2015^117^ | India | Cross sectional | 100 | Continuous Hb (g/L) | 2nd/ 3rd trimester | Adjusted beta coefficient:  𝛽= 0.043 (95% CI: 0.00, 0.09) |
| Khoushabi, 2010^118^ | Iran | Cross sectional | 500 | Continuous Hb (g/dL) | 3^rd^ trimester | Unadjusted beta coefficient, kg (SE):  𝛽= 0.816 (0.207) |
| Koyuncu, 2017^119^ | Turkey | Cross sectional | 433 | Hb < 8.5 g/dL, 8.5 - 11 g/dL, > 11 g/dL | 3^rd^ trimester | Unadjusted difference in mean birth weight, g (SD):  < 8.5 g/dL: 3,357.5 ± 256  8.5 - 11: 3,203.7 ± 152  > 11: 3,110.4 ± 203  p-value=0.562 |
| Kuizon, 1985^120^ | Philippines | Cross sectional | 428 | Continuous Hb (g/dL) | During pregnancy | Adjusted beta coefficient, g:  𝛽= 10.57 |
| Kumar, 2013^121^ | India | Retrospective cohort | 1,000 | Anemia: < 110 g/L | 1^st^ trimester  2^nd^ trimester  3^rd^ trimester | Unadjusted means, kg:  1^st^ trimester:  Anemic: 2.91 kg; non-anemic: 2.98 kg  2^nd^ trimester:  Anemic: 2.90 kg; non-anemic: 2.99 kg  3^rd^ trimester:  Anemic: 2.88 kg; non-anemic: 2.98 kg |
| Mathews, 2004^122^ | United Kingdom | Prospective cohort | 798 | Continuous Hb (g/L) | 2nd trimester 3rd trimester | 2^nd^ trimester: 𝛽= 100.00 (95% CI: -218.0–418.0)  3^rd^ trimester: 𝛽= -615.00 (95% CI: -944.0– -285.0) |
| Mezdoud, 2017^123^ | Algeria | Cross sectional | 97 | Maternal Hb (g/dL) | Predelivery | Correlation coefficient: r = 0.22, p-value= 0.02 |
| Mitchell, 1992^124^ | United States | Retrospective cohort | 1,080 | Continuous Hb (g/L) | 3^rd^ trimester | Correlation coefficient: r=0.21 |
| Moghaddam Tabrizi, 2012^125^ | Iran | Prospective cohort | 450 | Continuous Hb (g/dL) | 1^st^ trimester  2^nd^ trimester  3^rd^ trimester | Adjusted beta-coefficient (SE):  𝛽= 1.523 (0.416) |
| Moghaddam Tabrizi, 2015^126^ | Iran | Prospective cohort | 1,405 | Mild anemia: 8.1-10 g/dL  Moderate anemia: 6.5-8 g/dL | 1^st^ trimester  2^nd^ trimester  3^rd^ trimester | Unadjusted mean, g (SD):  1^st^ trimester:  Mild: 2,701 (512)  Moderate: 2,609 (431)  None: 3,216 (724)  2^nd^ trimester:  Mild: 2,697 (610)  Moderate: 2,615 (611)  No anemia: 3,271 (504)  3^rd^ trimester:  Mild: 2,737 (701)  Moderate: 2,604 (634)  No anemia: 3,301 (623) |
| Msuya, 2011^55^ | Tanzania | Prospective cohort | 2,654 | Moderate anemia: 70- 89 g/L Severe anemia: < 70 g/L | 3rd trimester | Unadjusted mean, g (SD):  Severe: 2,923 g ± 458  Moderate: 3,121 ± 451  Non-anemic: 3,152 ± 458 |
| Nahum, 2004^127^ | United States | Retrospective cohort | 235 | Continuous Hb (g/L) | 3rd trimester | 𝛽= -0.32 (95% CI: -0.52 – -0.11) |
| NamliKalem, 2017^128^ | Turkey | Prospective cohort | 380 | Maternal Hb (g/dL) | Predelivery | Spearman's correlation coefficient:  0.062 (p = 0.245) |
| Ngare, 1998^129^ | Kenya | Prospective cohort | 148 | Continuous Hb (g/dL) | During pregnancy | Mean Hb, g/dL (SD):  LBW: 10.7 g/dL (1.40)  Normal BW: 11.9 g/dL (1.27) |
| Nordenvall, 1990^130^ | Sweden | Prospective cohort | 330 | High Hb: > 130 g/L | 2nd trimester | 𝛽= -132.00 (95% CI: -273.12 – 9.12) |
| Orlandini, 2017^131^ | Italy | Retrospective cohort | 1,131 | Anemia: < 11 g/dL | 3^rd^ trimester | Unadjusted mean, g (SD):  Anemic: 3,406 (380)  Non-anemic: 3,305 (410) |
| Rasmussen, 1993^132^ | Norway | Prospective cohort | 3,074 | Low Hb: < 11.0 g/dL  High Hb: ≥ 14.0 g/dL | 1^st^ trimester  2^nd^ trimester | Unadjusted mean, g (SD):  1^st^ trimester:  < 11.0: 3,470 g (574)  11.0 ≤ Hb < 14.0: 3,516 g (583)  ≥ 14.0: 3,383 g (597)  2^nd^ trimester  < 11.0: 3,590 g (581)  11.0 ≤ Hb < 14.0: 3,499 g (579)  ≥ 14.0: 3,372 g (673) |
| Singh, 1998^133^ | Singapore | Case control | 3,728 | < 7 g/dL  7 ≤ Hb < 9 g/dL  9 ≤ Hb < 11 g/dL  ≥ 11 g/dL | Predelivery | Unadjusted means, g:  < 7 g/dL: 3,035  7 ≤ Hb < 9 g/dL: 3,043  9 ≤ Hb < 11 g/dL: 3,132  ≥ 11 g/dL: 3,091 |
| Singla, 1997^134^ | India | Case control | 76 | ≤ 6 g/dL  6.1 ≤ Hb ≤ 8.5 g/dL  8.6 ≤ Hb ≤ 10.9 g/dL  ≥ 11 g/dL | Predelivery | Unadjusted means, g (SD):  ≤ 6 g/dL: 2,107 (92)  6.1 ≤ Hb ≤ 8.5 g/dL: 2,363 (237)  8.6 ≤ Hb ≤ 10.9 g/dL: 2,710 (262)  ≥ 11 g/dL: 2,844 (345) |
| Smithers, 2014^77^ | Australia^1^ | Retrospective cohort | 96,290 | Anemia: <110 g/L | 1st trimester | Adjusted mean difference, g (95% CI):  36 (95% CI: 23 – 49) |
| Tarim, 2004^135^ | Turkey | Prospective cohort | 253 | High Hb: ≥ 12.2 g/dL | 1^st^ trimester | Unadjusted means, g (SD):  Hb: ≥ 12.2 g/dL: 3039.00 g ± 548.89  Hb: < 12.2 g/dL: 3333.27 g ± 510.86  p-value = 0.007 |
| Telatar, 2009^136^ | Turkey | Cross sectional | 3,688 | Anemia: Hb < 110 g/L | Predelivery | Crude OR (95% CI):  1.74 (1.03, 2.94) |
| Thame, 1997^137^ | Jamaica | Retrospective cohort | 2,394 | Hb: < 95, 95- 105, 106- 115, 116 - 125, > 125 g/L | 1st trimester 2nd trimester 3rd trimester | Unadjusted mean, g: 1st: < 95 g/L: 2,958, 95 - 105 g/L: 3,227, 106 - 115 g/L: 3,190, 116 - 125 g/L: 3,198, > 125 g/L: 3,189  2nd: < 95 g/L: 3,226, 95 - 105 g/L: 3,241, 106 - 115 g/L: 3,201, 116 - 125 g/L: 3,202, > 125 g/L: 3,128  3rd: < 95 g/L: 3,167, 95 - 105 g/L: 3,218, 106 - 115 g/L: 3,290, 116 - 125 g/L: 3,199, > 125 g/L: 3,115 |
| Tunkyi, 2018^138^ | South Africa | Retrospective cohort | 1,433 | Anemia: Hb < 11 g/dL | 3^rd^ trimester | Unadjusted difference in mean birth weight, kg (SD):  Anemic: 2.2 ± 0.8  Non-anemic: 2.9 ± 0.6  p-value = 0.40 |
| Ugwuja, 2009^139^ | Nigeria | Prospective cohort | 351 | Anemia: Hb < 110 g/L | 2nd trimester | Unadjusted difference in means, g (SD):  Anaemic: 3.02 g (0.52)  non-anaemic: 3.12 g (0.46)  p-value = 0.806 |
| Van Bogaert, 2006^140^ | South Africa | Prospective cohort | 3,214 | Continuous Hb (g/dL) | 2^nd^ trimester | Slope (SE): 0.18 (1.7) |
| von Tempelhoff, 2008^141^ | Germany | Retrospective cohort | 4,985 | Continuous Hb (g/L) | 2nd trimester | Correlation coefficient (r): -0.07 (p-value < 0.001) |
| Walker, 2003^86^ | Jamaica | Retrospective cohort | 234 | Low Hb: < 95 g/L | 2nd trimester | Slope (SE), kg:  𝛽= -0.21 (0.08) |
| Whittaker, 1996^142^ | United Kingdom | Prospective cohort | 69 | Continuous Hb (g/dL) | Preconception  1^st^ trimester  3^rd^ trimester | Correlation coefficient:  Preconception: -0.12  1^st^ trimester: -0.05  3^rd^ trimester: -0.32 |
| Williams, 1997^143^ | Australia | Prospective cohort | 2,507 | Anaemia: < 110 g/L | During pregnancy | 𝛽= 96.8 (95% CI: 61.8 – 131.8) |
| Yildiz, 2014^144^ | Turkey | Retrospective cohort | 28,600 | Hb: < 100, 100- 110, > 110 g/L | 3rd trimester | Unadjusted mean, g (SD): Hb < 100 g/L: 3,071 (707) Hb 100 - 110 g/L: 3,100 (671) Hb > 110 g/L: 3,135 (628) |
| *Gestational Age* | | |  |  |  |  |
| Abdel-Raoufabdel-AzizAfifi 2013^96^ | Egypt | Prospective cohort | 206 | Mild anemia: 80 - 105 g/L Severe anemia: < 80 g/L | During pregnancy | Unadjusted means, g (SD):  Mild anemia: 3,219 (0.3342)  Severe anemia: 3.272 (0.3124)  No anemia: 3.0702 (0.43195) |
| Allen, 1998^145^ | Papua New Guinea | Prospective cohort | 987 |  | During pregnancy | Crude OR (95% CI):  0.64 (0.46, 0.89) |
| Alwan, 2015^146^ | United Kingdom | Retrospective cohort | 362 | Anemia: ≤ 20 weeks: <110 g/L >20 weeks: < 105 g/L | 2nd trimester | Adjusted difference in means:  ≤ 20 weeks gestation: -6.8 (-13.6 – 0.1)  > 20 weeks gestation: -3.7 (-0.4 – 7.8) |
| Arbuckle, 1989^147^ | Canada | Prospective cohort | 984 | Continuous Hb (g/100 ml) | During pregnancy | Crude OR:  1.06 (0.84, 1.33) |
| Baraka, 2012^99^ | Belgium | Cross-sectional | 341 | Anemia: < 11 g/dL | 1^st^ trimester | Unadjusted means, weeks (SD):  Anemic: 39.5 ± 0.4 weeks  Non-anemic: 39.7 ± 0.2 weeks  p-value = 0.804 |
| Duthie, 1991^105^ | Hong Kong | Case control | 96 | Severe anemia: < 8 g/dL | During pregnancy | Unadjusted mean, weeks (SD):  Severe anemia: 38.5 (4.7)  Controls: 39 (1.5) |
| Koyuncu, 2017^119^ | Turkey | Cross sectional | 433 | Hb < 8.5 g/dL, 8.5 - 11 g/dL, > 11 g/dL | 3^rd^ trimester | Unadjusted difference in mean gestational age, weeks (SD):  < 8.5 g/dL: 38.2 ± 6  8.5 - 11: 38.3 ± 4  > 11: 38.5 ± 2  p-value=0.562 |
| Kumar, 2010^148^ | India | Prospective cohort | 2,027 | Continuous Hb (g/dL) | 1^st^ trimester | Correlation coefficient: r=0.040 |
| Kumar, 2013^121^ | India | Retrospective cohort | 1,000 | Anemia: < 110 g/L | 1^st^ trimester  2^nd^ trimester  3^rd^ trimester | Unadjusted means, weeks:  1^st^ trimester- Anemic: 37.9 weeks; non-anemic: 38.2 weeks  2^nd^ trimester- Anemic: 37.9 weeks; non-anemic: 38.3 weeks  3^rd^ trimester: Anemic: 37.7 weeks, non-anemic: 38.4 weeks |
| Laflamme, 2010^112^ | Bolivia | Retrospective cohort | 98 | Anemic: Hb < 110 g/L | 1st trimester | Unadjusted mean, weeks: Anemic: 37.1 Non-anemic: 38.9  p-value = 0.059 |
| Lao, 2000^111^ | China | Prospective cohort | 437 | Anemia: < 10 g/dL | 3^rd^ trimester | Unadjusted means, weeks (SD):  Anemia: 38.3 (2.0)  No anemia: 39.2 (1.3)  p-value = 0.004 |
| Lee, 2006^113^ | Korea | Prospective cohort | 248 | Continuous Hb (g/L) | Predelivery | Correlation coefficient: r= 0.258 |
| Lelic, 2014^114^ | Bosnia and Herzegovina | Case control | 100 | Anemia: Hb < 110 g/L | At labor and on 2 previous occasions in current pregnancy | Unadjusted mean, weeks (SD): Anemic: 38.2 (0.95) Non-anemic: 39.2 (0.88)  p-value= <0.0001 |
| Luis, 2016^116^ | United Kingdom | Case control | 212 | Anemia: < 8 g/dL | During pregnancy | Unadjusted difference in means, weeks:  Cases: 38.9  Controls: 39.2  p-value= 0.52 |
| Menon, 2016^149^ | India | Prospective cohort | 178 | Anemia: 2nd trimester: < 105 g/L 3rd trimester: < 110 g/L | 2nd trimester 3rd trimester | Difference of means, weeks (95% CI):  Second trimester: 0.15 (95% CI: -0.45 – 0.75)  Third trimester: -0.17 (95% CI: -0.77 – 0.43) |
| Mitchell, 1992^124^ | United States | Retrospective cohort | 1,080 | Continuous Hb (g/L) | 3^rd^ trimester | Correlation coefficient: r=0.29 |
| Nansook, 2018^150^ | South Africa | Prospective cohort | 327 | Continuous Hb (g/dL) | 1^st^ trimester | 𝛽= 0.080 (SE = 0.068) |
| Rasmussen, 1993^132^ | Norway | Prospective cohort | 3,074 | Low Hb: < 11.0 g/dL  High Hb: ≥ 14.0 g/dL | 1^st^ trimester  2^nd^ trimester | Unadjusted mean (SD):  1^st^ trimester:  < 11.0: 278 days (12.9)  11.0 ≤ Hb < 14.0: 278 days (13.6)  ≥ 14.0: 275 days (15.7)  2^nd^ trimester:  < 11.0: 279 days (12.4)  11.0 ≤ Hb < 14.0: 278 days (13.9)  ≥ 14.0: 276 days (14.6) |
| Smithers, 2014^77^ | Australia^5^ | Retrospective cohort | 96,290 | Anemia: <110 g/L | 1st trimester | Adjusted mean difference, weeks (95% CI):  -0.05 (95% CI: -0.09 – -0.004) |
| Tarim, 2004^135^ | Turkey | Prospective cohort | 253 | High Hb: ≥ 12.2 g/dL | 1^st^ trimester | Unadjusted mean, weeks (SD):  Hb: ≥ 12.2 g/dL: 37.9651 weeks ± 2.10  Hb: < 12.2 g/dL: 38.5151 weeks ± 1.57 |
| Ugwuja, 2009^139^ | Nigeria | Prospective cohort | 351 | Anemia: Hb < 110 g/L | 2nd trimester | Unadjusted mean, weeks (SD):  Anaemic: 38.96 weeks (1.79) Non-anaemic: 39.42 weeks (1.59)  p-value = 0.023 |
| von Tempelhoff, 2008^141^ | Germany | Retrospective cohort | 4,985 | Continuous Hb (g/L) | 2nd trimester | Correlation coefficient (r): -0.06 (p-value=0.04) |
| *Birth Length (cm)* | |  |  |  |  |  |
| Abdel-Raoufabdel-AzizAfifi 2013^96^ | Egypt | Prospective cohort | 206 | Mild anemia: 80 - 105 g/L Severe anemia: < 80 g/L | During pregnancy | Unadjusted mean, cm (SD): Hb 80- 105 g/L: 51.73 (1.561) Hb < 80 g/L: 50.22 (2.106) |
| Ali, 2009^98^ | Sudan | Case control | 125 | Anemia: Hb < 110 g/L | Predelivery | Unadjusted mean, cm (SD): Anemic: 48.64 (2.27) Non-anemic: 49.08 (2.25) |
| Bhargava, 2000^151^ | Kenya | Retrospective cohort | 102 | Continuous Hb (g/L) | During pregnancy | 𝛽= - 0.013 (SE = 0.034) |
| de Sa, 2015^103^ | Brazil | Cross sectional | 54 | Anemia: Hb < 110 g/L | Predelivery | Unadjusted mean, cm (SD): Anemic: 51.2 (1.7) Non-anemic: 50.3 (2.0) |
| Emamghorashi, 2004^106^ | Iran | Cross sectional | 97 | Anemia: Hb < 110 g/L | Predelivery | Unadjusted mean, cm: IDA: 50.1  NAID: 50.2 NC: 50.1 |
| Kaur, 2015^117^ | India | Cross sectional | 100 | Continuous Hb (g/L) | 2nd/ 3rd trimester | Adjusted beta coefficient:  𝛽= 0.265 (95% CI: -0.17, 0.72) |
| LaFlamme, 2010^112^ | Bolivia | Retrospective cohort | 98 | Anemic: Hb < 110 g/L | 1st trimester | Unadjusted mean, cm (SD): Anemic: 47.8  Non-anemic: 47.5 |
| Lao, 2002^43^ | China | Prospective cohort | 730 | Anemia: Hb < 100 g/L | First antenatal visit | Unadjusted mean, cm (SD):  < 115 g/L: 49.3 (3.3) 116- 123 g/L: 49.6 (3.0) 124- 130 g/L: 49.2 (3.9)  > 130 g/L: 49.2 (3.9) |
| Lee, 2006^113^ | Korea | Prospective cohort | 248 | Hb: < 108, 108- 119, ≥ 120 g/L | Predelivery | Unadjusted mean, cm (SD): Hb < 108 g/L: 47.06 (5.82) Hb 108 - 119 g/L: 48.60 (4.46) Hb ≥ 120 g/L: 49.26 (1.69) |
| Levario-Carrillo, 2003^115^ | Mexico | Cross sectional | 153 | Anemia: Hb < 110 g/L | Predelivery | Unadjusted mean, cm (SD): Anemic: 53 (4) Non-anemic: 53 (3) |
| Lelic, 2014^114^ | Bosnia and Herzegovina | Case control | 100 | Anemia: Hb < 110 g/L | At labor and on 2 previous occasions in current pregnancy | Unadjusted mean, cm (SD): Anemic: 51.76 (2.82) Non-anemic: 55.54 (2.32) |
| Msuya, 2011^55^ | Tanzania | Prospective cohort | 2,654 | Moderate anemia: 70- 89 g/L Severe anemia: < 70 g/L | 3rd trimester | Unadjusted mean, cm (SD): Hb 70 - 109 g/L: 49.0 (1.9) Hb < 70 g/L: 48.5 (1.98) |
| Ronnenberg, 2004^68^ | China | Prospective cohort | 405 | Low Hb: <95, 95-120 g/L High Hb: > 120 g/L | Preconception | Difference in least squares means: Hb < 95 g/L: 49.4 Hb 95- 120 g/L: 50.0 Hb > 120 g/L: 50.3 |
| Telatar, 2009^136^ | Turkey | Cross sectional | 3,688 | Anemia: Hb < 110 g/L | Predelivery | Unadjusted mean, cm (SD): Anemic: 48.44 (2.16) Non-anemic: 48.29 (2.12) |
| Ugwuja, 2009^139^ | Nigeria | Prospective cohort | 351 | Anemia: Hb < 110 g/L | 2nd trimester | Unadjusted mean, cm (SD): Anemic: 51.23 (5.39) Non-anemic: 50.49 (3.02) |
| Yildiz, 2014^144^ | Turkey | Retrospective cohort | 28,600 | Hb: < 100, 100- 110, > 110 g/L | 3rd trimester | Unadjusted mean, cm (SD): Hb < 100 g/L: 49.02 (4.0) Hb 100 - 110 g/L: 49.35 (3.6) Hb > 110 g/L: 49.51 (3.3) |
| *Head Circumference (cm)* | |  |  |  |  |  |
| Bhargava, 2000^151^ | Kenya | Retrospective cohort | 102 | Continuous Hb (g/L) | During pregnancy | 𝛽= 0.0033 (SE=0.023 ) |
| de Sa, 2015^103^ | Brazil | Cross sectional | 54 | Anemia: Hb < 110 g/L | Predelivery | Unadjusted mean, cm (SD): Anemic: 34.5 (1.5) Non-anemic: 34.2 (2.0) |
| Emamghorashi, 2004^106^ | Iran | Cross sectional | 97 | Anemia: Hb < 110 g/L | Predelivery | Unadjusted mean, cm: IDA: 35.6 NAID: 34.6 NC: 34.5 |
| Kaur, 2015^117^ | India | Cross sectional | 100 | Continuous Hb (g/L) | 2nd/ 3rd trimester | Adjusted beta coefficient:  𝛽= 0.152 (-0.02, 0.32) |
| Khoigani, 2012^152^ | Iran | Prospective cohort | 520 | Continuous Hb (g/L) | 1st trimester 3rd trimester | p-value > 0.05 |
| LaFlamme, 2010^112^ | Bolivia | Retrospective cohort | 98 | Anemic: Hb < 110 g/L | 1st trimester | Unadjusted mean, cm (SD): Anemic: 34.2 Non-anemic: 39.5 |
| Ronnenberg, 2004^68^ | China | Prospective cohort | 405 | Low Hb: <95, 95-120 g/L High Hb: > 120 g/L | Preconception | Difference in least squares means: Hb < 95 g/L: 33.0 Hb 95- 120 g/L: 33.4 Hb > 120 g/L: 33.9 |
| Singla, 1997^134^ | India | Case control | 76 | Anemia: ≤ 60, 61- 85, 86- 109 g/L Non-anemic: ≥ 110 g/L | Predelivery | Unadjusted mean, cm (SD): Hb ≤ 60 g/L: 32.6 (0.7) Hb 61 - 85 g/L: 33.2 (0.9) Hb 86 - 109 g/L: 33.7 (0.9) Hb ≥ 110 g/L: 34.0 (1.2) |
| Telatar, 2009^136^ | Turkey | Cross sectional | 3,688 | Anemia: Hb < 110 g/L | Predelivery | Unadjusted mean, cm (SD) Anemic: 34.45 (1.32) Non-anemic: 34.34 (1.34) |
| Thame, 1997^137^ | Jamaica | Retrospective cohort | 2,394 | Hb: < 95, 95- 105, 106- 115, 116 - 125, > 125 g/L | 1st trimester 2nd trimester 3rd trimester | Unadjusted mean, cm (SD): 1st: < 95 g/L: 34.4, 95 - 105 g/L: 34.8, 106 - 115 g/L: 34.4, 116 - 125 g/L: 34.4, > 125 g/L: 34.4  2nd: < 95 g/L: 34.8, 95 - 105 g/L: 34.5, 106 - 115 g/L: 34.5, 116 - 125 g/L: 34.1, > 125 g/L: 34.2  3rd: < 95 g/L: 34.7, 95 - 105 g/L: 34.8, 106 - 115 g/L: 34.8, 116 - 125 g/L: 34.5, > 125 g/L: 34.0 |
| Ugwuja, 2009^139^ | Nigeria | Prospective cohort | 351 | Anemia: Hb < 110 g/L | 2nd trimester | Unadjusted mean, cm (SD): Anemic: 33.41 (2.68) Non-anemic: 34.09 (2.61) |
| *Cardiovascular outcomes^2^* | |  |  |  |  |  |
| Alwan, 2014^153,2a^ | United Kingdom | Prospective cohort | 2,958 | Anemia: Hb < 110 g/L | 1st/ 2nd trimester | Systolic BP: 𝛽= -1.0 (-4.6, 2.6) Diastolic BP: 𝛽= -0.3 (-3.4, 2.8) |
| Alwan, 2015^146,2b^ | United Kingdom | Retrospective cohort | 362 | Anemia: Hb < 110 g/L | ≤ 20 weeks gestation > 20 weeks gestation | Change in infant bfpWV: ≤ 20 weeks gestation: 1.00 (0.10, 1.90) > 20 weeks gestation: 0.01 (-0.500, 0.50) |
| Belfort, 2008^154,2c^ | United States | Prospective cohort | 1,167 | Continuous Hb (g/L) | 1st trimester 2nd trimester | 1st trimester: 𝛽= 0.5 (-0.2, 1.1) 2nd trimester: 𝛽= 0.2 (-0.5, 0.9) |
| Bergel, 2000^155,2d^ | Argentina | Prospective cohort | 518 | Continuous Hb (g/L) | Lowest Hb during pregnancy | Standardized regression coefficient= 1.3 (0.4, 2.3) |
| Brion, 2008^156,2e^ | United Kingdom | Prospective cohort | 1,255 | Anemia: Hb < 110 g/L | Early pregnancy 3rd trimester | Early pregnancy: Systolic BP: 𝛽= -1.36 (-3.18, 0.46) Diastolic BP: 𝛽= -1.45 (-2.79, -0.12) 3rd trimester: Systolic BP: 𝛽= -0.49 (-1.71, 0.72) Diastolic BP: 𝛽= -0.34 (-1.22, 0.53) |
| Chou, 2016^157,2f^ | Taiwan | Retrospective cohort | 1,387,650 | Anemia: Hb < 120 g/L | During pregnancy | OR= 1.31 (1.25, 1.38) |
| Godfrey, 1994^158,2g^ | Jamaica | Prospective cohort | 77 | Continuous Hb (g/L) | Lowest Hb during pregnancy | Systolic BP: 𝛽= 0.0 (-1.9, 1.8) |
| Welten, 2015^159,2h^ | Netherlands | Prospective cohort | 5,002 | Anemia: Hb < 110 g/L High Hb: ≥ 132.1 g/L | 2nd trimester | Anemic: Systolic BP: 𝛽= 0.27 (-0.41, 0.96) Diastolic BP: 𝛽= 0.70 (0.12, 1.29) High Hb: Systolic BP: 𝛽= -0.04 (-0.67, 0.59) Diastolic BP: 𝛽= -0.20 (-0.74, 0.34) |
| *Child Development^3^* | |  |  |  |  |  |
| Aranda, 2017^160^ | Spain | Prospective cohort | 210 | Anemia: < 110 g/L | 3^rd^ trimester  Delivery | 3rd trimester:  State regulation cluster: 𝛽 =-0.061 (p =0.809)  Alert quality: 𝛽 = -0.376 (p = 0.320)  Delivery:  State regulation cluster: 𝛽 = -0.079 (p = 0.804)  Robustness and endurance: 𝛽 = -0.492 (p 0.304) |
| ElAlfy, 2018^161^ | Egypt | Case control | 50 | Anemia: < 11 g/dL | Predelivery | Passed Auditory Brainstem Response (ABR) screening test: 10.16 ± 0.49 g/dL  Failed ABR screening test: 9.59 ± 1.14 g/dL  p-value = 0.028 |
| Fararouei, 2010^162,3a^ | Finland | Prospective cohort | 11,656 | Continuous Hb (g/L) | 3rd month, 7th month, 9th month | 3rd month: OR= 1.00 (0.95, 1.05) 7th month: OR= 1.01 (0.96, 1.06) 9th month: OR= 1.02 (0.97, 1.07) |
| Menon, 2016^149,3b^ | India | Prospective cohort | 178 | Anemia: 2nd trimester: Hb < 105 g/L 3rd trimester: Hb < 110 g/L | 2nd trimester 3rd trimester | Study presented adjusted difference of means data for 7 measures of behavioral assessment (abnormal reflex, habituation, orientation, range of state, regulation of state, motor maturity, and autonomic stability. All were non-significant at p< 0.05 aside from range of state (-0.83, -1.56 to -0.09). |
| Mireku, 2015^163,3c^ | Benin | Prospective cohort | 636 | Anemia: Hb < 110 g/L | 2nd trimester 3rd trimester Predelivery | Mean difference: ELC Score: 2nd trimester: -1.00 (-3.40, 1.40) 3rd trimester: 0.50 (-1.80, 2.80) Delivery: -0.70 (-3.10, 1.70) GM Score:  2nd trimester: 0.40 (-2.00, 2.70) 3rd trimester: 2.20 (-0.20, 4.50) Delivery: -1.10 (-3.50, 1.40) |
| Yang, 2010^164^ | China | Prospective cohort | 3,609 | Hb: < 103 g/L | Early gestation | High maternal Hb concentrations during early gestation may adversely affect cognitive development in pre-school aged children. Poor verbal scores: OR= 1.54 (1.13, 2.11) Poor full-scale scores: OR= 1.53 (1.10, 2.12) |
| *Schizophrenia ^4^* |  |  |  |  |  |  |
| Ellman, 2012^165,4a^ | United States | Case control | 46 | Continuous Hb (g/L) | Mean value during pregnancy | Study examined if fetal exposure to decreases in maternal Hb was associated to diminished neuromotor and neurocognitive performance among cases with schizophrenia and SSDs and matched controls. Results showed that decreases in Hb led to significant decreases in scores on the Grooved Pegboard test, finger tapping test, and WAIS information subtest. |
| Insel, 2008^166,4b^ | United States | Retrospective cohort | 6,872 | Low Hb: ≤ 100 g/L Moderate Hb: ≤ 120 g/L | 1st trimester 2nd trimester 3rd trimester | ≤ 100 g/L 1st trimester: IDR= 1.14 (0.14, 9.35) 2nd trimester: IDR= 3.13 (1.08, 9.09) 3rd trimester: IDR= 3.09 (1.24, 7.70) > 100 g/L and < 120 g/L: 1st trimester: IDR= 0.68 (0.26, 1.76) 2nd trimester: IDR= 1.77 (0.73, 4.29) 3rd trimester: IDR= 1.87 (0.90, 3.91) |
| Nielsen, 2016^167,4c^ | Denmark | Prospective cohort | 1,403,183 | Anemia: Hb < 110 g/L | During pregnancy | IDR= 1.20 (0.92, 1.53) |
| *Child Morbidities^5^* | | | | | | |
| Alemu, 2016^168,5a^ | Ethiopia | Cross sectional | 11,872 | Anemia: Hb < 110 g/L | During pregnancy | OR= 1.1 (1.02, 1.64) |
| Kattula, 2014^38,5b^ | India | Prospective cohort | 420 | Anemia: Hb < 100 g/L | 2nd trimester | Crude HR= 0.99 (0.86, 1.14) |
| Knottnerus, 1990^41,5c^ | Netherlands | Prospective cohort | 796 | Continuous Hb (mmol/l) | 3rd trimester | 𝛽=0.159 (0.298) |
| Savajols, 2014^169,5d^ | France | Prospective cohort | 409 | Anemia: Hb ≤ 107 g/L High Hb: ≥125 g/L | Predelivery | Anemia: OR= 2.97 (1.36, 6.47) High Hb: 0.78 (0.31, 1.98) |
| Triche, 2011^170,5e^ | United States | Retrospective cohort | 597 | Anemia: Hb < 110 g/L | Predelivery | Year 1 recurrent wheeze: OR= 2.11 (1.12, 3.98) Wheeze before age 3: OR= 2.36 (1.34, 4.17) Asthma diagnosis ever: OR=1.60 (0.83, 3.07) Asthma diagnosis/ wheeze before age 6: OR= 1.76 (0.90, 3.43) |
| *SIDS* |  |  |  |  |  |  |
| Klonoff-Cohen, 2002^171^ | United States | Case control | 478 | Anemia: Hb ≤ 120 g/L | 1st trimester 2nd trimester 3rd trimester | OR (95% CI) = 2.51 (1.25, 5.03) |
| *Infant mortality* | | | | | | |
| Agarwal, 1998^172^ | India | Prospective cohort | 6,790 | High Hb: > 100 g/L | 3rd trimester | Crude RR= 1.51 (0.89, 2.54) |
| *Allergic sensitization* | | | | | | |
| Shaheen, 2017^173^ | United Kingdom | Prospective cohort | 13,758 | Hb ≤ 11.8 g/dL, 11.9 - 12.3 g/dL, 12.4 - 12.7 g/dL, 12.8 - 13.2 g/dL, ≥ 13.3. g/dL | First measurement (< 18 weeks gestation)  Last measurement (> 28 weeks gestation) | Adjusted OR (95% CI):  First measurement:  Hb ≤ 11.8 g/dL: 1.06 (0.84, 1.34)  11.9 - 12.3 g/dL: 1.28 (1.02, 1.61)  12.8 - 13.2 g/dL: 0.93 (0.74, 1.18)  ≥ 13.3. g/dL: 0.94 (0.74, 1.20)  Last measurement:  Hb ≤ 11.8 g/dL: 1.00 (0.80, 1.26)  11.9 - 12.3 g/dL: 0.89 (0.71, 1.12)  12.8 - 13.2 g/dL: 0.83 (0.66, 1.05)  ≥ 13.3. g/dL: 0.76 (0.60, 0.96) |
| *Type I Diabetes* | | | | | | |
| Stordal, 2018^174^ | Norway | Prospective cohort | 94,209 | Hb < 10.5 g/dL | During pregnancy | Adjusted HR (95% CI):  1.13 (0.83, 1.54) |
| Hb, hemoglobin; OR, Odds Ratio; RR, Relative Risk; IDR, Rate Ratio; HR, Hazard Ratio, BP, Blood Pressure; SD, standard deviation ^1^ Cardiovascular outcomes- a: BP at 10 years; b: infant PW; c: BP at 3 years; d: BP at 5-9 years; e: BP at 7 years; f: congenital heart disease in offspring; g: BP at 10-12 years; h: BP at 6 years ^2^ Child development outcomes- a: Data for highest level of education at age of 31 years presented (data also available for total school scores at age 14, theory scores at age 14, and total school scores at age 16); b: Scores from Neonatal Behavioral Assessment in full term infants at ~3 weeks postpartum; c: Infant gross motor (GM) scores and Early Learning Composite scores (fine motor, language reception, language expression, and visual reception) assessed in 1 year old children ^3^ Schizophrenia outcomes- a: outcomes examined include full scale IQ estimate, WAIS information SS, picture completion SS, grooved pegboard dominant (log), grooved pegboard non-dominant, finger tapping, CVLT short delay z-scores, CVLT long delay z-scores, WAIS digit span SS, letter-number SS, trails B-Trails A times (log), MWCST errors (log); b: Schizophrenia spectrum disorders (SSDs); c: Rate of schizophrenia in offspring over 17.6 million person-years of follow up ^4^ Child Morbidity outcomes- a: small size, depends on subjective evaluation of the baby's size at birth by the mother b: child morbidity defined as GI illnesses (diarrhea or vomiting lasting for more than 24 hours), upper respiratory illnesses (runny nose or cough lasting 5+ days), undifferentiated fever (not associated with other symptoms lasting at least 48 hours); c: Composite outcome of low birth weight (< 2,500 g) and/or preterm birth (< 37 weeks); d: poor short term neonatal outcomes defined as death during hospital stay and/or grades III–IV intraventricular haemorrhage (defined as per the Papile classification), and/or cystic periventricular leukomalacia and/or necessity of ventriculoperitoneal shunt for post hemorrhagic hydrocephaly; e: Year 1 recurrent wheeze, wheeze before age 3, asthma diagnosis, asthma diagnosis and wheeze at age 6   \| **OSM Table S4. Summary of Other Maternal Health Outcomes (insufficient data for meta-analysis)** \| \| \| \| \| \| \| \| --- \| --- \| --- \| --- \| --- \| --- \| --- \| \| Study (Author, year) \| Setting \| Study Design \| Sample Size \| Exposure \| Timing of Exposure \| Statistical Measure \| \| *Eclampsia* \|  \|  \|  \|  \|  \|  \| \| Ali, 2011^4^ \| Sudan \| Case control \| 9,578 \| Mild/moderate anemia: Hb 70- 109 g/L Severe anemia: Hb < 70 g/L \| Predelivery \| OR (95% CI) = 2.30 (0.70, 7.80) OR (95% CI) = 4.00 (0.70, 20.90) \| \| Rukuni, 2016^69^ \| United Kingdom \| Retrospective cohort \| 80,422 \| Anemia: Hb < 100 g/L \| Hb at booking \| OR (95% CI) = 0.77 (0.24, 2.51) \| \| *Prenatal Depression* \|  \|  \|  \|  \|  \|  \| \| Babu, 2018^175^ \| India \| Cross sectional \| 823 \| Anemia: Hb < 11 g/dL \| During pregnancy \| OR (95% CI) = 1.92 (1.17, 3.15) \| \| Woldetensay, 2018^176^ \| Ethiopia \| Prospective cohort \| 4,680 \| Anemia: Hb < 11 g/dL \| During pregnancy \| OR (95% CI) = 1.30 (1.04, 1.61) \| \| *Postpartum Depression^2^* \| \|  \|  \|  \|  \|  \| \| Goshtasebi, 2013^177,2a^ \| Iran \| Prospective cohort \| 254 \| Anemia: Hb < 110 g/L \| Predelivery \| OR (95% CI) =4.64 (1.33, 16.08) \| \| Lukose, 2014^178,2b^ \| India \| Cross sectional \| 365 \| Anemia: Hb < 110 g/L \| 1st trimester \| PR (95% CI) = 0.67 (0.47, 0.96) \| \| Raisanen, 2014^179,2c^ \| Finland \| Cross sectional \| 511,938 \| Anemia: Hb ≤ 100 g/L \| During pregnancy \| OR (95% CI) =1.49 (1.22, 1.81) \| \| Yilmaz, 2017^180,2d^ \| Turkey \| Cross sectional \| 450 \| Anemia: Hb < 110 g/L \| 3rd trimester \| r= -0.203 \| \| Xu, 2018^181^ \| Australia \| Retrospective cohort \| 345,049 \| Anemia (ICD-10 diagnosis) \| During pregnancy \| OR (95% CI)=1.62 (1.25, 2.11) \| \| *Other maternal morbidities^3^* \| \|  \|  \|  \|  \|  \| \| Hanprasertpong, 2015^182,3a^ \| Thailand \| Retrospective cohort \| 240 \| Anemia: Hb < 110 g/L \| During pregnancy \| OR (95% CI) = 3.03 (1.14, 8.05) \| \| Nair, 2014^183,3b^ \| United Kingdom \| Case control \| 5,063 \| Anemia: Hb < 90 g/L \| During pregnancy \| OR (95% CI) = 1.80 (0.99, 3.26) \| \| Tzur, 2012^83,3c^ \| Israel \| Retrospective cohort \| 33,888 \| Anemia: <100 g/L \| 1st trimester \| Crude OR (95% CI) = 0.82 (0.70, 1.00) \| \| *Maternal Mortality* \|  \|  \|  \|  \|  \|  \| \| Azulay, 2015^184,4^ \| Israel \| Retrospective cohort \| 47,657 \| Anemia: Hb < 100 g/L \| 3rd trimester \| Crude OR (95% CI) = 1.70 (0.80, 3.50) \| \| Daru, 2018^185^ \| Multi-country \| Cross sectional \| 12,470 \| Severe anemia: < 70 g/L \| During pregnancy \| OR (95% CI) = 2.36 (1.60, 3.48) \| \| Gonzales, 2012^31^ \| Peru \| Retrospective cohort \| 379,816 \| Low Hb: < 70, 70- <90, 90- <110 g/L High Hb: >145 g/L \| 3rd trimester \| <70 g/L: OR (95% CI) = 8.54 (2.06, 35.42) 70- < 90 g/L: OR (95% CI) = 6.06 (3.16, 11.6) 90 - < 110 g/L: OR (95% CI) = 1.07 (0.65, 1.77) > 145 g/L: OR (95% CI) = 2.01 (1.12, 3.61) \| \| Buchmann, 2015^186^ \| South Africa \| Cross sectional \| 479 \| Anemia: Hb < 110 g/L \| First antenatal visit \| Crude OR (95% CI) = 4.00 (2.30, 6.90) \| \| Rukuni, 2016^69^ \| United Kingdom \| Retrospective cohort \| 80,422 \| Anemia: Hb < 100 g/L \| Hb at booking \| OR (95% CI) = 2.07 (0.22, 19.36) \| \| Hb, hemoglobin; OR, Odds Ratio; RR, Relative Risk; PR, prevalence ratio; r, correlation coefficient ^1^ Outcome was defined as presence of either gestational diabetes mellitus or pregnancy-induced hypertension ^2^ Depression outcomes: a: postpartum depression; b: antenatal depression; c: major depression during pregnancy; d: Depressive symptoms; EPDS score ^3^ Other maternal morbidities: a: composite adverse maternal outcome defines as preeclampsia, GDM, preterm birth, postpartum hemorrhage, puerperal morbidity, or surgical wound complication; b: severe maternal morbidity directly attributed to antenatal pulmonary embolism, eclampsia, AFLP, amniotic fluid embolism, peripartum hysterectomy, stroke in pregnancy, uterinerupture, placenta accreta, HELLP, and severe sepsis; b: hypertension; c: composite outcome; c: hypertension ^4^ Study assessed cardiovascular mortality \| \| \| \| \| \| \| \| \| \| \| \| \| \| \|  \| **OSM Table S5. Summary of Studies in High Risk Populations** \| \| \|  \|  \|  \|  \| \| --- \| --- \| --- \| --- \| --- \| --- \| --- \| \| Study (Author, year) \| Setting \| Study Design \| Sample Size \| Exposure \| Time of Exposure \| Outcome \| \| *Women with HIV* \|  \|  \|  \|  \|  \|  \| \| Bloch, 2015^187^ \| South Africa \| Cross sectional \| 15,725 \| Low Hb: ≤ 92 g/L \| 3rd trimester \| Hemorrhage, transfusion \| \| Chatterjee, 2007^188^ \| Tanzania \| Prospective cohort \| 939 \| Low Hb: < 85 g/L \| Predelivery \| Child mortality \| \| Ezechi, 2013^189^ \| Nigeria \| Cross sectional \| 563 \| Anemia: Hb < 110 g/L \| First clinic attendance \| Asymptomatic bacteriuria \| \| Li, 2014^190^ \| Tanzania \| Prospective cohort \| 21,645 \| Anemia: Hb < 100 g/L \| During pregnancy \| Maternal mortality \| \| Liotta, 2013^191^ \| Mozambique and Malawi \| Retrospective cohort \| 10,150 \| Low Hb: <80 g/L, 80- 100 g/L \| Baseline Hb \| Maternal mortality \| \| Machado, 2014^192^ \| Latin America, Caribbean \| Prospective cohort \| 1,513 \| Hb ≥ 110 g/L \| Predelivery \| Preeclampsia \| \| O'Brien, 2005^193^ \| Tanzania \| Cross sectional \| 1,078 \| Moderate anemia: Hb 85-109 g/L Severe anemia: Hb < 85 g/L \| During pregnancy \| Maternal mortality \| \| Obimbo, 2004^194^ \| Kenya \| Prospective cohort \| 62 \| Severe anemia: Hb < 80 g/L \| 3rd trimester \| Child mortality \| \| Traisathit, 2009^195^ \| Thailand \| Prospective cohort \| 979 \| Hb ≥ 115 g/L \| 3rd trimester \| PTB \| \| Van den Broek, 2014^196^ \| Malawi \| Retrospective cohort \| 2,149 \| Anemia: Hb < 110 g/L \| First antenatal visit \| PTB \| \| Young, 2012^197^ \| Uganda \| Prospective cohort \| 158 \| Mild to moderate anemia: Hb 85- 109 g/L Severe anemia: ≤ 85 g/L \| 2nd trimester \| SGA \| \| *Young Adolescents (< 15 years)* \| \|  \|  \|  \|  \|  \| \| Alizadeh, 2014^198^ \| Iran \| Cross sectional \| 312 \| Anemia: Hb < 110 g/L \| 3rd trimester \| LBW \| \| Al-Sibai, 1987^199^ \| Saudi Arabia \| Retrospective cohort \| 206 \| Anemia: Hb < 106 g/L \| During pregnancy \| LBW \| \| Chang, 2003^200^ \| United States \| Retrospective cohort \| 918 \| Low Hb: ≤ 105 g/L High Hb: > 120 g/L \| 2nd trimester 3rd trimester \| LBW, PTB \| \| Pinho-Pompeu, 2017^201^ \| Brazil \| Cross sectional \| 458 \| Mild anemia: Hb 100- 109 g/L Moderate anemia: Hb 70- 99 g/L Severe anemia: Hb < 70 g/L \| During pregnancy \| PTB, stillbirth, LBW, SGA \| \| Scholl, 1984^202^ \| United States \| Retrospective cohort \| 64 \| Continuous Hb (g/L) \| Predelivery \| BW \| \| *Women with preeclampsia* \| \|  \|  \|  \|  \|  \| \| Amburgey, 2009^203^ \| United States \| Retrospective cohort \| 142 \| Continuous Hb (g/L) \| Highest Hb \| LBW \| \| Sak, 2012^204^ \| Turkey \| Retrospective cohort \| 167 \| Continuous Hb (g/L) \| During pregnancy \| Maternal mortality \| \| *Twin pregnancies* \|  \|  \|  \|  \|  \|  \| \| Kosto, 2016^205^ \| Israel \| Retrospective cohort \| 247 \| Anemia: Hb < 100 g/L \| 2nd trimester \| LBW, PTB, SGA, fetal malformation, PPH, GDM, transfusion, preeclampsia, perinatal mortality \| \| Shumpert, 2004^206^ \| United States \| Retrospective cohort \| 80,495 \| Anemia: Hb < 100 g/L \| During pregnancy \| PTB, SGA, stillbirth, neonatal mortality, perinatal mortality \| \| *Other health conditions* \| \|  \|  \|  \|  \|  \| \| Berhan, 2014^207,1b^ \| Ethiopia \| Retrospective cohort \| 432 \| Anemia: Hb < 120 g/L \| At admission \| Perinatal mortality \| \| Bo, 2016^208,1d^ \| China \| Retrospective cohort \| 60 \| Continuous Hb (g/L) \| During pregnancy \| Maternal and fetal complications \| \| Cyganek, 2014^209,1c^ \| Poland \| Retrospective cohort \| 100 \| Anemia: Hb < 110 g/L \| During pregnancy \| IUGR \| \| Sehgal, 2016^210,1e^ \| India \| Prospective cohort \| 377 \| Mild anemia: Hb 100- 109 g/L Moderate anemia: Hb 70-100 g/L Severe anemia: Hb < 70 g/L \| 3rd trimester \| Postpartum events, transfusion, IUGR \| \| Yang, 2015^211,1f^ \| China \| Retrospective cohort \| 25 \| Severe anemia: Hb < 70 g/L \| During pregnancy \| Poor maternal/ fetal outcome \| \| Zaren, 1997^212,1a^ \| Sweden and Norway \| Prospective cohort \| 1,037 \| Continuous Hb (g/L) \| During pregnancy \| LBW, BL \| \| Hb, hemoglobin; SGA, small for gestational age; PTB, preterm birth; LBW, low birth weight; BW, birth weight; PPH, postpartum hemorrhage; GDM, gestational diabetes mellitus; BL, birth length; IUGR, intrauterine growth restriction ^1^ Other health conditions- a: study conducted in women with placenta previa/ placental abruption; b: study conducted in women with aplastic anemia; c: study conducted in renal/ liver transplant recipients; d: study conducted in primigravida mothers; e: study conducted in women with myelodysplastic syndrome; f: study conducted in smoking mothers \| \| \| \| \| \| \| \| \| \| | | | | | | |
|  |  |  |  |  |  |  |
|  |  |  |  |  |  |  |
|  |  |  |  |  |  |  |
|  |  |  |  |  |  |  |
|  |  |  |  |  |  |  |
|  |  |  |  |  |  |  |
|  |  |  |  |  |  |  |
|  |  |  |  |  |  |  |
|  |  |  |  |  |  |  |
|  |  |  |  |  |  |  |
|  |  |  |  |  |  |  |
|  |  |  |  |  |  |  |
|  |  |  |  |  |  |  |
|  |  |  |  |  |  |  |
|  |  |  |  |  |  |  |
|  |  |  |  |  |  |  |

| **OSM Table S6. Summary of Child Health Outcomes with Other Statistical Measures.** | | |
| --- | --- | --- |
| Statistical measure | No. | Included studies |
| ***Low Birth Weight*** | | |
| Crude Relative Risk | 4 | Bakhtiar 2007^213^, Bhalerao 2011^214^, Hirve 1994^215^, Shobeiri 2006^216^ |
| Chi-square test/ Fisher's Exact test/ ANOVA | 7 | Murphy 1986^217^, Ahmad 1997^218^, Bakacak 2015^219^, Misra 2015^220^, Yu 2014^221^, Onadeko 1996^222^, Lin 2018^223^ |
| Crude OR | 13 | Hosain 2006^224^, Kumar 2010^148^, Misra 2015^220^, Levy 2005^44^, Sekhavat 2011^225^, Kidanto 2009^226^, Oladeinde 2015^227^, Yildiz 2014^144^, Singh 2009^228^, Castaldi 2006^229^, Brabin 1990^230^, Ahankari 2017^231^, Gardner 2018^232^ |
| z-score | 1 | Cordina 2015^233^ |
| N (%), p-value, or graphs only presented | 7 | Lao 2002^43^, RaghuRaman 2001^234^, Yazdani 2004^235^, Burrows 1988^236^, Ma 2009^237^, Mitchell 1987^238^, Suryanarayana 2017^239^ |
| Continuous Hb (g/L) | 1 | Rizvi 2007^240^, Stanisic 2015^241^ |
| ***Preterm Birth*** | | |
| Crude Relative Risk | 2 | Bakhtiar 2007^213^, Bhalerao 2011^214^ |
| Chi-square/ Fisher's exact test/ ANOVA | 4 | Murphy 1986^217^, Yuan 2010^242^, Weidinger 1974^243^, Lin 2018^223^ |
| Crude OR | 9 | Sullivan 1999^244^, Levy 2005^44^, Beta 2013^245^, Tabussum 1994^246^, Marti 2001^50^, Kidanto 2009^226^, Abrams 2004^247^, Fahim 1992^248^, Wagura 2018^249^, Zhang 2009^250^, Menon 2016^149^ |
| N (%), p-value, or graphs only presented | 4 | Lao 2002^43^, Yazdani 2004^235^, Tunkyi 2018^138^, DincgezCakmak 2018^251^ |
| Continuous Hb (g/L) | 1 | Von Tempelhoff 2008^141^ |
| ***Stillbirth*** | | |
| Crude Relative Risk | 3 | Bakhtiar 2007^213^, Bhalerao 2011^214^, Jehan 2007^252^ |
| Hazard Ratio | 1 | Tomashek 2006^253^, Zhang 2009^254^ |
| Chi-square test | 4 | Little 2005^255^, Malhotra 2002^47^, Luis 2016^116^, Onadeko 1996^222^ |
| Crude OR | 1 | Kidanto 2009^226^ |
| N (%) or p-value only | 3 | Suryanarayana 2017^239^, Tunkyi 2018^138^, DincgezCakmak 2018^251^ |
| Crude beta coefficient | 1 | Nair 2018^256^ |
| ***SGA/ IUGR*** | | |
| Crude Relative Risk | 1 | Lone 2004^45^ |
| Chi-square test/ ANOVA | 3 | Malhotra 2002^47^, Mau 1977^257^, Rasmussen 1993^132^ |
| Crude OR | 4 | Sullivan 1999^244^, Levy 2005^44^, Delpisheh 2008 (asymmetric/ symmetric)^19^, Arbuckle 1989^147^ |
| z-score | 2 | Sawant 2013^258^, Cordina 2015^233^ |
| N (%), p-value, or graphs only presented | 2 | Narang 1997^259^, Chhabra 1996^260^ |
| Continuous Hb (g/L) | 1 | Von Tempelhoff 2008^141^ |
| ***Neonatal Mortality*** | | |
| Hazard Ratio | 1 | Zhang 2009^254^ |
| Chi-square test | 2 | Little 2005^255^, Malhotra 2002^47^ |
| Crude OR | 1 | Kidanto 2009^226^ |
| p-value | 1 | Tunkyi 2018^138^ |
| ***Perinatal Mortality*** | | |
| Relative Risk | 4 | Bakhtiar 2007^213^, Bhalerao 2011^214^, Lone 2004^45^, Agarwal 1998^172^ |
| Chi-square test | 1 | Murphy 1986^217^ |
| Crude OR | 1 | Levy 2005^44^ |
| N (%), p-value, or graphs only presented | 2 | Serour 1981^261^, Msuya 2011^55^ |
| No., Number; OR, Odds ratio; SGA, small for gestational age; IUGR, intrauterine growth restriction | | |

| **OSM Table S7. Summary of Maternal Health Outcomes Studies with Other Statistical Measures.** | | |
| --- | --- | --- |
| Statistical measure | No. | Included studies |
| ***PPH*** | | |
| Chi-square value | 1 | Malhotra 2002^47^ |
| Crude OR | 2 | Tzur 2012^83^, Rubio-Alvarez^262^ |
| Unadjusted means | 3 | Kavle 2008^263^, Orlandini 2017^131^, Luis 2016^116^ |
| N (%) | 2 | Suryanarayana 2017^239^, DincgezCakmak 2018^251^ |
| Continuous Hb (g/L) | 1 | Biguzzi 2012^264^ |
| ***Transfusion*** | | |
| Chi-square value | 1 | Malhotra 2002^47^ |
| Crude OR | 3 | Levy 2005^44^, Tzur 2012^83^, Petty 2018^265^ |
| ***Preeclampsia*** | | |
| Chi-square value | 2 | Malhotra 2002^47^, Morgan-Ortiz 2010^266^ |
| Crude Relative Risk | 1 | Mehrabian 2013^267^ |
| z-score | 1 | Cordina 2015^268^ |
| Continuous Hb- OR | 1 | Von Tempelhoff 2008^141^ |
| N (%) | 1 | Suryanarayana 2017^239^ |
| ***GDM*** | | |
| Crude OR | 4 | Nombo, 2018^269^, Tandu-Umba 2015^80^, Tzur 2012^83^, Banhidy 2011^8^ |
| Continous Hb- OR | 1 | Alshareef 2018^270^ |
| Chi-square test | 2 | Raisanen 2014^64^, Macaulay 2018^271^ |
| Crude RR | 2 | Mehrabian 2013^267^, Nastaran 2012^272^ |
| No., Number; OR, Odds Ratio; PPH, Postpartum hemorrhage; GDM, gestational diabetes mellitus. | | |

| OSM Table S8. Number of studies assessing maternal hemoglobin concentrations and birth outcomes available for meta-analysis by hemoglobin concentration cut-off. | | | | | | |
| --- | --- | --- | --- | --- | --- | --- |
|  | LBW | PTB | SGA | Stillbirth | Perinatal Mortality | Neonatal Mortality |
| Hemoglobin Cut-off | **Number of Studies** | | | | | |
| ≤ 70 g/L | 4 studies | 2 studies | 1 study | 3 studies | 1 study |  |
| ≤ 80 g/L | 8 studies | 3 studies | 4 studies | 5 studies | 3 studies | 1 study |
| ≤ 90 g/L | 13 studies | 9 studies | 8 studies | 7 studies | 5 studies | 1 study |
| ≤ 100 g/L | 24 studies | 20 studies | 15 studies | 12 studies | 9 studies | 5 studies |
| ≤ 110 g/L | 36 studies | 33 studies | 21 studies | 16 studies | 11 studies | 5 studies |
| ≥ 120 g/L | 7 studies | 13 studies | 7 studies | 5 studies | 2 studies |  |
| ≥ 130 g/L | 6 studies | 12 studies | 6 studies | 5 studies | 1 study |  |
| ≥ 140 g/L | 3 studies | 8 studies | 3 studies | 4 studies |  |  |
| ≥ 150 g/L | 1 study | 3 studies | 2 studies |  |  |  |
| ≥ 160 g/L | 1 study | 1 study | 1 study |  |  |  |
| Preconception (< 110 g/L) | 2 studies | 3 studies | 2 studies | 1 study |  |  |
| First trimester (< 110 g/L) | 9 studies | 11 studies | 8 studies | 2 studies | 3 studies | 1 study |
| Second trimester (< 110 g/L) | 6 studies | 6 studies | 5 studies | 3 studies | 2 studies |  |
| Third trimester (< 110 g/L) | 12 studies | 12 studies | 7 studies | 6 studies | 3 studies |  |
| Overall Estimate < 110 g/L | 36 studies | 33 studies | 21 studies | 21 studies | 11 studies | 5 studies |
| Preconception (> 130 g/L) | 1 study | 2 studies | 1 study | 1 study |  |  |
| First trimester (> 130 g/L) | 2 studies | 5 studies | 3 studies | 2 study |  |  |
| Second trimester (> 130 g/L) | 1 study | 3 studies | 2 studies | - |  |  |
| Third trimester (> 130 g/L) | 1 study | 2 studies | 1 study | 1 study |  |  |
| Overall Estimate >130 g/L | 5 studies | 10 studies | 4 studies | 4 studies | - |  |
| Total number of studies | **41 studies** | **40 studies** | **25 studies** | **19 studies** | **11 studies** | **5 studies** |

| OSM Table S9. Number of studies assessing maternal hemoglobin concentrations and maternal  outcomes available for meta-analysis by hemoglobin concentration cut-off. | | | | |
| --- | --- | --- | --- | --- |
|  | PPH | Pre-eclampsia | Transfusion | GDM |
| ≤ 80 g/L | 1 study | 3 studies | 1 study |  |
| ≤ 90 g/L | 3 studies | 3 studies | 1 study |  |
| ≤ 100 g/L | 6 studies | 5 studies | 3 studies |  |
| ≤ 110 g/L | 6 studies | 8 studies | 4 studies | 2 studies |
| ≥ 120 g/L | 1 study | 7 studies |  | 4 studies |
| ≥ 130 g/L | 1 study | 3 studies |  | 3 studies |
| ≥ 140 g/L | 1 study | 2 studies |  | 2 studies |
| Overall Estimate < 110 g/L | 6 studies | 8 studies | 4 studies | 2 studies |
| Overall Estimate >130 g/L | 1 study | 3 studies | -- | 3 studies |
| Total number of studies | **7 studies** | **9 studies** | **4 studies** | **4 studies** |

PPH, post-partum hemorrhage; GDM, gestational diabetes mellitus.

**OSM Figure S1.** Overall meta-analysis for association between maternal hemoglobin concentration (low: < 110 g/L; high: > 130 g/L) measured at any point during pregnancy and low birth weight (< 2,500 g). Reference groups for each study included in the meta-analysis are listed under ‘Hb ref’.

The following studies provided cut-off estimates for multiple timepoints and/or disease phenotypes: Bodeau-Livinec, 2011 (2^nd^ trimester, 3^rd^ trimester); Hamaleinen, 2003 (1^st^, 2^nd^, 3^rd^ trimester); Xiong, 2003 (1^st^ visit, 3^rd^ trimester).

**OSM Figure S2.** Overall meta-analysis for association between maternal hemoglobin concentration (low: < 110 g/L; high: > 130 g/L) measured at any point during pregnancy and preterm birth (< 37 completed weeks gestation). Reference groups for each study included in the meta-analysis are listed under ‘Hb ref’.

The following studies provided cut-off estimates for multiple timepoints and/or phenotypes: Hamaleinen, 2003 (1^st^, 2^nd^, 3^rd^ trimester); Raisanen, 2014 (multiparous, nulliparous); Xiong, 2003 (1^st^ visit, 3^rd^ trimester); Raisanen, 2013 (extremely preterm, very preterm, moderately preterm); Ribot, 2014 (smokers, non-smokers); Zhang, 2018 (1^st^ trimester, 2^nd^ trimester)

**OSM Figure S3.** Overall meta-analysis for association between maternal hemoglobin concentration (low: < 110 g/L; high: > 130 g/L) measured at any point during pregnancy and small for gestational age (birth weight < 10^th^ centile for gestational age). Reference groups for each study included in the meta-analysis are listed under ‘Hb ref’. The following studies provided cut-off estimates for multiple timepoints and/or disease phenotypes: Ota, 2014 (preterm, term); Hamaleinen, 2003 (1^st^, 2^nd^, 3^rd^ trimester); Raisanen, 2014 (nulliparous, multiparous); Xiong, 2003 (1^st^ visit, 3^rd^ trimester); Chen, 2017 (early pregnancy, late pregnancy).

**OSM Figure S4.** Overall meta-analysis for association between maternal hemoglobin concentration (low: < 110 g/L; high: > 130 g/L) measured at any point during pregnancy and stillbirth. Reference groups for each study included in the meta-analysis are listed under ‘Hb ref’. The following studies provided cut-off estimates for multiple timepoints and/or disease phenotypes: Nair, 2017 (1^st^ visit, 3^rd^ trimester); Raisanen, 2014 (nulliparous, multiparous); Maghsoudlou, 2016 (before pregnancy, 2^nd^ trimester).

**OSM Figure S5.** Overall meta-analysis for association between low maternal hemoglobin concentration (< 110 g/L) measured at any point during pregnancy and perinatal mortality. Reference groups for each study included in the meta-analysis are listed under ‘Hb ref’. The following studies provided cut-off estimates for multiple timepoints and/or disease phenotypes: Nair, 2017 (1^st^ visit, 3^rd^ trimester); Xiong, 2003 (1^st^ visit, 3^rd^ trimester).

**OSM Figure S6.** Overall meta-analysis for association between low maternal hemoglobin concentration (< 110 g/L) measured at any point during pregnancy and neonatal mortality. Reference groups for each study included in the meta-analysis are listed under ‘Hb ref’. The following studies provided cut-off estimates for multiple timepoints and/or disease phenotypes: Raisanen, 2014 (nulliparous vs. multiparous).

**OSM Figure S7.** Overall meta-analysis for association between low maternal hemoglobin concentration (< 110 g/L) measured at any point during pregnancy and post-partum hemorrhage. Reference groups for each study included in the meta-analysis are listed under ‘Hb ref’. The following studies provided cut-off estimates for multiple timepoints and/or disease phenotypes: Butwick, 2017 (prelabor, intrapartum cesarean delivery); Rukuni, 2016 (post-partum hemorrhage, major obstetric hemorrhage).

**OSM Figure S8.** Overall meta-analysis for association between low maternal hemoglobin concentration (< 110 g/L) measured at any point during pregnancy and transfusion. Reference groups for each study included in the meta-analysis are listed under ‘Hb ref’. The following studies provided cut-off estimates for multiple timepoints and/or disease phenotypes: Ehrenthal, 2012 (cesarean delivery, normal delivery).

**OSM Figure S9.** Overall meta-analysis for association between maternal hemoglobin concentration (low: < 110 g/L; high: > 130 g/L) measured at any point during pregnancy and pre-eclampsia. Reference groups for each study included in the meta-analysis are listed under ‘Hb ref’. The following studies provided cut-off estimates for multiple timepoints and/or disease phenotypes: Chen, 2018 (nulliparous, multiparous); Raisanen, 2014

(nulliparous, multiparous).

**OSM Figure S10.** Overall meta-analysis for association between high maternal hemoglobin concentration (> 130 g/L) measured at any point during pregnancy and gestational diabetes. Reference groups for each study included in the meta-analysis are listed under ‘Hb ref’.

**OSM Figure S11.** Meta-analysis summary estimates for association between low maternal hemoglobin (< 110 g/L) and low birth weight (< 2,500 g) by timing of hemoglobin measurement during pregnancy**.** Overall pregnancy estimates are meta-analysis summary estimates from studies that measured hemoglobin concentrations at any time point during pregnancy. A complete list of studies included in the overall summary estimate can be found in OSM Figure S6.

**OSM Figure S12.** Meta-analysis summary estimates for association between high maternal hemoglobin (> 130 g/L) and low birth weight (< 2,500 g) by timing of hemoglobin measurement during pregnancy**.** Overall pregnancy estimates are meta-analysis summary estimates from studies that measured hemoglobin concentrations at any time point during pregnancy. A complete list of studies included in the overall summary estimate can be found in OSM Figure S6.

**OSM Figure S13.** Meta-analysis summary estimates for association between low maternal hemoglobin (< 110 g/L) and preterm birth (< 37 completed weeks gestation) by timing of hemoglobin measurement during pregnancy**.** Overall pregnancy estimates are meta-analysis summary estimates from studies that measured hemoglobin concentrations at any time point during pregnancy. A complete list of studies included in the overall summary estimate can be found in OSM Figure S7.

**OSM Figure S14.** Meta-analysis summary estimates for association between high maternal hemoglobin (> 130 g/L) and preterm birth (< 37 completed weeks gestation) by timing of hemoglobin measurement during pregnancy**.** Overall pregnancy estimates are meta-analysis summary estimates from studies that measured hemoglobin concentrations at any time point during pregnancy. A complete list of studies included in the overall summary estimate can be found in OSM Figure S7.

**OSM Figure S15.** Meta-analysis summary estimates for association between low maternal hemoglobin (< 110 g/L) and small for gestational age (birth weight < 10th centile for gestational age) by timing of hemoglobin measurement during pregnancy**.** Overall pregnancy estimates are meta-analysis summary estimates from studies that measured hemoglobin concentrations at any time point during pregnancy. A complete list of studies included in the overall summary estimate can be found in OSM Figure S8.

**OSM Figure S16.** Meta-analysis summary estimates for association between high maternal hemoglobin (> 130 g/L) and small for gestational age (birth weight < 10th centile for gestational age) by timing of hemoglobin measurement during pregnancy**.** Overall pregnancy estimates are meta-analysis summary estimates from studies that measured hemoglobin concentrations at any time point during pregnancy. A complete list of studies included in the overall summary estimate can be found in OSM Figure S8.

References

1. Abeysena C, Jayawardana P, de ASR. Maternal haemoglobin level at booking visit and its effect on adverse pregnancy outcome. *Aust N Z J Obstet Gynaecol.* 2010;50(5):423-427.

2. Adam I, Haggaz AD, Mirghani OA, Elhassan EM. Placenta previa and pre-eclampsia: analyses of 1645 cases at medani maternity hospital, Sudan. *Front Physiol.* 2013;4:32.

3. Adams MM, Sarno AP, Harlass FE, Rawlings JS, Read JA. Risk factors for preterm delivery in a healthy cohort. *Epidemiology.* 1995;6(5):525-532.

4. Ali AA, Rayis DA, Abdallah TM, Elbashir MI, Adam I. Severe anaemia is associated with a higher risk for preeclampsia and poor perinatal outcomes in Kassala hospital, eastern Sudan. *BMC Res Notes.* 2011;4:311.

5. Alwan NA, Cade JE, McArdle HJ, Greenwood DC, Hayes HE, Simpson NA. Maternal iron status in early pregnancy and birth outcomes: insights from the Baby's Vascular health and Iron in Pregnancy study. *Br J Nutr.* 2015;113(12):1985-1992.

6. Bader E, Alhaj AM, Hussan AA, Adam I. Malaria and stillbirth in Omdurman Maternity Hospital, Sudan. *Int J Gynaecol Obstet.* 2010;109(2):144-146.

7. Baig SA, Khan N, Baqai T, Fatima A, Karim SA, Aziz S. Preterm birth and its associated risk factors. A study at tertiary care hospitals of Karachi, Pakistan. *J Pak Med Assoc.* 2013;63(3):414-418.

8. Banhidy F, Acs N, Puho EH, Czeizel AE. Iron deficiency anemia: pregnancy outcomes with or without iron supplementation. *Nutrition.* 2011;27(1):65-72.

9. Bian Y, Zhang Z, Liu Q, Wu D, Wang S. Maternal risk factors for low birth weight for term births in a developed region in China: a hospital-based study of 55,633 pregnancies. *J Biomed Res.* 2013;27(1):14-22.

10. Bilano VL, Ota E, Ganchimeg T, Mori R, Souza JP. Risk factors of pre-eclampsia/eclampsia and its adverse outcomes in low- and middle-income countries: a WHO secondary analysis. *PLoS One.* 2014;9(3):e91198.

11. Bodeau-Livinec F, Briand V, Berger J, et al. Maternal anemia in Benin: prevalence, risk factors, and association with low birth weight. *Am J Trop Med Hyg.* 2011;85(3):414-420.

12. Borah M, Agarwalla R. Maternal and socio-demographic determinants of low birth weight (LBW): A community-based study in a rural block of Assam. *J Postgrad Med.* 2016;62(3):178-181.

13. Butwick AJ, Ramachandran B, Hegde P, Riley ET, El-Sayed YY, Nelson LM. Risk Factors for Severe Postpartum Hemorrhage After Cesarean Delivery: Case-Control Studies. *Anesth Analg.* 2017.

14. Chen JH, Guo XF, Liu S, et al. [Impact and changes of maternal hemoglobin on birth weight in pregnant women of Zhuang Nationality, in Guangxi]. *Zhonghua Liu Xing Bing Xue Za Zhi.* 2017;38(2):154-157.

15. Chen C, Grewal J, Betran AP, Vogel JP, Souza JP, Zhang J. Severe anemia, sickle cell disease, and thalassemia as risk factors for hypertensive disorders in pregnancy in developing countries. *Pregnancy Hypertens.* 2018;13:141-147.

16. Chumak EL, Grijbovski AM. Anemia in pregnancy and its association with pregnancy outcomes in the Arctic Russian town of Monchegorsk, 1973–2002. *International Journal of Circumpolar Health.* 2016;69(3):265-277.

17. Chumak EL, Grijbovski AM. Association between different levels of hemoglobin in pregnancy and pregnancy outcomes: a registry-based study in Northwest Russia. *International Journal of Circumpolar Health.* 2016;70(5):457-459.

18. Cung TG, Paus AS, Aghbar A, Kiserud T, Hinderaker SG. Stillbirths at a hospital in Nablus, 2010: a cohort study. *Glob Health Action.* 2014;7:25222.

19. Delpisheh A, Brabin L, Drummond S, Brabin BJ. Prenatal smoking exposure and asymmetric fetal growth restriction. *Ann Hum Biol.* 2008;35(6):573-583.

20. Domple VK, Doibale MK, Nair A, Rajput PS. Assessment of maternal risk factors associated with low birth weight neonates at a tertiary hospital, Nanded, Maharashtra. *Niger Med J.* 2016;57(1):37-43.

21. Drukker L, Hants Y, Farkash R, Ruchlemer R, Samueloff A, Grisaru-Granovsky S. Iron deficiency anemia at admission for labor and delivery is associated with an increased risk for Cesarean section and adverse maternal and neonatal outcomes. *Transfusion.* 2015;55(12):2799-2806.

22. Ehrenthal DB, Chichester ML, Cole OS, Jiang X. Maternal risk factors for peripartum transfusion. *J Womens Health (Larchmt).* 2012;21(7):792-797.

23. Elhassan EM, Abbaker AO, Haggaz AD, Abubaker MS, Adam I. Anaemia and low birth weight in Medani, Hospital Sudan. *BMC Res Notes.* 2010;3:181.

24. Eng C, Karki S, Trivedi AN. Risk factors of stillbirths in Victoria (Australia): A case-control study. *J Obstet Gynaecol.* 2016;36(6):754-757.

25. Ferdous J, Ahmed A, Dasgupta SK, et al. Occurrence and determinants of postpartum maternal morbidities and disabilities among women in Matlab, Bangladesh. *J Health Popul Nutr.* 2012;30(2):143-158.

26. Gaillard R, Eilers PH, Yassine S, Hofman A, Steegers EA, Jaddoe VW. Risk factors and consequences of maternal anaemia and elevated haemoglobin levels during pregnancy: a population-based prospective cohort study. *Paediatr Perinat Epidemiol.* 2014;28(3):213-226.

27. Ganesh Kumar S, Harsha Kumar HN, Jayaram S, Kotian MS. Determinants of low birth weight: a case control study in a district hospital in Karnataka. *Indian J Pediatr.* 2010;77(1):87-89.

28. Geelhoed D, Agadzi F, Visser L, et al. Maternal and fetal outcome after severe anemia in pregnancy in rural Ghana. *Acta Obstet Gynecol Scand.* 2006;85(1):49-55.

29. Getiye Y, Fantahun M. Factors associated with perinatal mortality among public health deliveries in Addis Ababa, Ethiopia, an unmatched case control study. *BMC Pregnancy Childbirth.* 2017;17(1):245.

30. Gonzales GF, Steenland K, Tapia V. Maternal hemoglobin level and fetal outcome at low and high altitudes. *Am J Physiol Regul Integr Comp Physiol.* 2009;297(5):R1477-1485.

31. Gonzales GF, Tapia V, Gasco M, Carrillo CE, Fort AL. Association of hemoglobin values at booking with adverse maternal outcomes among Peruvian populations living at different altitudes. *Int J Gynaecol Obstet.* 2012;117(2):134-139.

32. Gonzales GF, Tapia V, Gasco M. Correcting haemoglobin cut-offs to define anaemia in high-altitude pregnant women in Peru reduces adverse perinatal outcomes. *Arch Gynecol Obstet.* 2014;290(1):65-74.

33. Hamalainen H, Hakkarainen K, Heinonen S. Anaemia in the first but not in the second or third trimester is a risk factor for low birth weight. *Clin Nutr.* 2003;22(3):271-275.

34. Hinderaker SG, Olsen BE, Bergsjo PB, Gasheka P, Lie RT, Kvale G. Perinatal mortality in northern rural Tanzania. *J Health Popul Nutr.* 2003;21(1):8-17.

35. Hwang HS, Kim YH, Kwon JY, Park YW. Uterine and umbilical artery Doppler velocimetry as a predictor for adverse pregnancy outcomes in pregnant women with anemia. *J Perinat Med.* 2010;38(5):467-471.

36. Jaleel R, Khan A. Post-partum haemorrhage--a risk factor analysis. *Mymensingh Med J.* 2010;19(2):282-289.

37. Kalanda BF, Verhoeff FH, Chimsuku L, Harper G, Brabin BJ. Adverse birth outcomes in a malarious area. *Epidemiol Infect.* 2006;134(3):659-666.

38. Kattula D, Sarkar R, Sivarathinaswamy P, et al. The first 1000 days of life: prenatal and postnatal risk factors for morbidity and growth in a birth cohort in southern India. *BMJ Open.* 2014;4(7):e005404.

39. Khan NS, Ashraf RN, Noor S, et al. ASSOCIATION OF MATERNAL PERIODONTITIS WITH LOW BIRTH WEIGHT IN NEWBORNS IN A TERTIARY CARE HOSPITAL. *J Ayub Med Coll Abbottabad.* 2016;28(1):120-125.

40. Khattar D, Awasthi S, Das V. Residential environmental tobacco smoke exposure during pregnancy and low birth weight of neonates: case control study in a public hospital in Lucknow, India. *Indian Pediatr.* 2013;50(1):134-138.

41. Knottnerus JA, Delgado LR, Knipschild PG, Essed GG, Smits F. Haematologic parameters and pregnancy outcome. A prospective cohort study in the third trimester. *J Clin Epidemiol.* 1990;43(5):461-466.

42. Koura GK, Ouedraogo S, Le Port A, et al. Anaemia during pregnancy: impact on birth outcome and infant haemoglobin level during the first 18 months of life. *Trop Med Int Health.* 2012;17(3):283-291.

43. Lao TT, Chan LY, Tam KF, Ho LF. Maternal hemoglobin and risk of gestational diabetes mellitus in Chinese women. *Obstet Gynecol.* 2002;99(5 Pt 1):807-812.

44. Levy A, Fraser D, Katz M, Mazor M, Sheiner E. Maternal anemia during pregnancy is an independent risk factor for low birthweight and preterm delivery. *Eur J Obstet Gynecol Reprod Biol.* 2005;122(2):182-186.

45. Lone FW, Qureshi RN, Emanuel F. Maternal anaemia and its impact on perinatal outcome. *Trop Med Int Health.* 2004;9(4):486-490.

46. Maghsoudlou S, Cnattingius S, Stephansson O, et al. Maternal haemoglobin concentrations before and during pregnancy and stillbirth risk: a population-based case-control study. *BMC Pregnancy Childbirth.* 2016;16(1):135.

47. Malhotra M, Sharma JB, Batra S, Sharma S, Murthy NS, Arora R. Maternal and perinatal outcome in varying degrees of anemia. *Int J Gynaecol Obstet.* 2002;79(2):93-100.

48. Mamun AA, Padmadas SS, Khatun M. Maternal health during pregnancy and perinatal mortality in Bangladesh: evidence from a large-scale community-based clinical trial. *Paediatr Perinat Epidemiol.* 2006;20(6):482-490.

49. Marchant T, Schellenberg JA, Nathan R, et al. Anaemia in pregnancy and infant mortality in Tanzania. *Trop Med Int Health.* 2004;9(2):262-266.

50. Marti A, Pena-Marti G, Munoz S, Lanas F, Comunian G. Association between prematurity and maternal anemia in Venezuelan pregnant women during third trimester at labor. *Arch Latinoam Nutr.* 2001;51(1):44-48.

51. Masukume G, Khashan AS, Kenny LC, Baker PN, Nelson G. Risk factors and birth outcomes of anaemia in early pregnancy in a nulliparous cohort. *PLoS One.* 2015;10(4):e0122729.

52. Meis PJ, Michielutte R, Peters TJ, et al. Factors associated with preterm birth in Cardiff, Wales. I. Univariable and multivariable analysis. *Am J Obstet Gynecol.* 1995;173(2):590-596.

53. Mohamed MA, Ahmad T, Macri C, Aly H. Racial disparities in maternal hemoglobin concentrations and pregnancy outcomes. *J Perinat Med.* 2012;40(2):141-149.

54. Mola G, Permezel M, Amoa AB, Klufio CA. Anaemia and perinatal outcome in Port Moresby. *Aust N Z J Obstet Gynaecol.* 1999;39(1):31-34.

55. Msuya SE, Hussein TH, Uriyo J, Sam NE, Stray-Pedersen B. Anaemia among pregnant women in northern Tanzania: prevalence, risk factors and effect on perinatal outcomes. *Tanzan J Health Res.* 2011;13(1):33-39.

56. Mumbare SS, Maindarkar G, Darade R, Yenge S, Tolani MK, Patole K. Maternal risk factors associated with term low birth weight neonates: a matched-pair case control study. *Indian Pediatr.* 2012;49(1):25-28.

57. Nair M, Churchill D, Robinson S, Nelson-Piercy C, Stanworth SJ, Knight M. Association between maternal haemoglobin and stillbirth: a cohort study among a multi-ethnic population in England. *British journal of haematology.* 2017;179(5):829-837.

58. Nyflot LT, Sandven I, Stray-Pedersen B, et al. Risk factors for severe postpartum hemorrhage: a case-control study. *BMC Pregnancy Childbirth.* 2017;17(1):17.

59. Obadi MA, Taher R, Qayad M, Khader YS. Risk factors of stillbirth in Yemen. *Journal of neonatal-perinatal medicine.* 2018;11(2):131-136.

60. Ota E, Ganchimeg T, Morisaki N, et al. Risk factors and adverse perinatal outcomes among term and preterm infants born small-for-gestational-age: secondary analyses of the WHO Multi-Country Survey on Maternal and Newborn Health. *PLoS One.* 2014;9(8):e105155.

61. Patel A, Prakash AA, Das PK, Gupta S, Pusdekar YV, Hibberd PL. Maternal anemia and underweight as determinants of pregnancy outcomes: cohort study in eastern rural Maharashtra, India. *BMJ Open.* 2018;8(8):e021623.

62. Phaloprakarn C, Tangjitgamol S. Impact of high maternal hemoglobin at first antenatal visit on pregnancy outcomes: a cohort study. *J Perinat Med.* 2008;36(2):115-119.

63. Poespoprodjo JR, Fobia W, Kenangalem E, et al. Adverse pregnancy outcomes in an area where multidrug-resistant plasmodium vivax and Plasmodium falciparum infections are endemic. *Clin Infect Dis.* 2008;46(9):1374-1381.

64. Raisanen S, Kancherla V, Gissler M, Kramer MR, Heinonen S. Adverse perinatal outcomes associated with moderate or severe maternal anaemia based on parity in Finland during 2006-10. *Paediatr Perinat Epidemiol.* 2014;28(5):372-380.

65. Raisanen S, Gissler M, Saari J, Kramer M, Heinonen S. Contribution of risk factors to extremely, very and moderately preterm births - register-based analysis of 1,390,742 singleton births. *PLoS One.* 2013;8(4):e60660.

66. Ren A, Wang J, Ye RW, Li S, Liu JM, Li Z. Low first-trimester hemoglobin and low birth weight, preterm birth and small for gestational age newborns. *Int J Gynaecol Obstet.* 2007;98(2):124-128.

67. Ribot B, Isern R, Hernandez-Martinez C, Canals J, Aranda N, Arija V. [Effects of tobacco habit, second-hand smoking and smoking cessation during pregnancy on newborn's health]. *Med Clin (Barc).* 2014;143(2):57-63.

68. Ronnenberg AG, Wood RJ, Wang X, et al. Preconception hemoglobin and ferritin concentrations are associated with pregnancy outcome in a prospective cohort of Chinese women. *J Nutr.* 2004;134(10):2586-2591.

69. Rukuni R, Bhattacharya S, Murphy MF, Roberts D, Stanworth SJ, Knight M. Maternal and neonatal outcomes of antenatal anemia in a Scottish population: a retrospective cohort study. *Acta Obstet Gynecol Scand.* 2016;95(5):555-564.

70. Saeed OA, Ahmed HA, Ibrahim AM, Mahmood EA, Abdu-Allah TO. Risk factors of low birth weight at three hospitals in Khartoum State, Sudan. *Sudan J Paediatr.* 2014;14(2):22-28.

71. Scanlon KS, Yip R, Schieve LA, Cogswell ME. High and low hemoglobin levels during pregnancy: differential risks for preterm birth and small for gestational age. *Obstet Gynecol.* 2000;96(5 Pt 1):741-748.

72. Schmiegelow C, Minja D, Oesterholt M, et al. Factors associated with and causes of perinatal mortality in northeastern Tanzania. *Acta Obstet Gynecol Scand.* 2012;91(9):1061-1068.

73. Scholl TO, Hediger ML, Fischer RL, Shearer JW. Anemia vs iron deficiency: increased risk of preterm delivery in a prospective study. *Am J Clin Nutr.* 1992;55(5):985-988.

74. Scholl TO, Hediger ML. Anemia and iron-deficiency anemia: compilation of data on pregnancy outcome. *Am J Clin Nutr.* 1994;59(2 Suppl):492S-500S discussion 500S.

75. Sharma SR, Giri S, Timalsina U, et al. Low birth weight at term and its determinants in a tertiary hospital of Nepal: a case-control study. *PLoS One.* 2015;10(4):e0123962.

76. Shehata N, Chasse M, Colas JA, et al. Risks and trends of red blood cell transfusion in obstetric patients: a retrospective study of 45,213 deliveries using administrative data. *Transfusion.* 2017;57(9):2197-2205.

77. Smithers LG, Gialamas A, Scheil W, Brinkman S, Lynch JW. Anaemia of pregnancy, perinatal outcomes and children's developmental vulnerability: a whole-of-population study. *Paediatr Perinat Epidemiol.* 2014;28(5):381-390.

78. Steer P, Alam MA, Wadsworth J, Welch A. Relation between maternal haemoglobin concentration and birth weight in different ethnic groups. *The BMJ.* 1995;310(6978):489-491.

79. Stephansson O, Dickman PW, Johansson A, Cnattingius S. Maternal hemoglobin concentration during pregnancy and risk of stillbirth. *Jama.* 2000;284(20):2611-2617.

80. Tandu-Umba B, Mbangama AM. Association of maternal anemia with other risk factors in occurrence of Great obstetrical syndromes at university clinics, Kinshasa, DR Congo. *BMC Pregnancy Childbirth.* 2015;15:183.

81. Thakur N, Saili A, Kumar A, Kumar V. Predictors of mortality of extremely low birthweight babies in a tertiary care centre of a developing country. *Postgrad Med J.* 2013;89(1058):679-684.

82. Tsu VD. Postpartum haemorrhage in Zimbabwe: a risk factor analysis. *Br J Obstet Gynaecol.* 1993;100(4):327-333.

83. Tzur T, Weintraub AY, Sergienko R, Sheiner E. Can anemia in the first trimester predict obstetrical complications later in pregnancy? *J Matern Fetal Neonatal Med.* 2012;25(11):2454-2457.

84. Unger HW, Ome-Kaius M, Karl S, et al. Factors associated with ultrasound-aided detection of suboptimal fetal growth in a malaria-endemic area in Papua New Guinea. *BMC Pregnancy Childbirth.* 2015;15:83.

85. Verhoeff FH, Brabin BJ, van Buuren S, et al. An analysis of intra-uterine growth retardation in rural Malawi. *Eur J Clin Nutr.* 2001;55(8):682-689.

86. Walker SP, Ewan-Whyte C, Chang SM, et al. Factors associated with size and proportionality at birth in term Jamaican infants. *J Health Popul Nutr.* 2003;21(2):117-126.

87. Wang C, Lin L, Su R, et al. Hemoglobin levels during the first trimester of pregnancy are associated with the risk of gestational diabetes mellitus, pre-eclampsia and preterm birth in Chinese women: a retrospective study. *BMC Pregnancy Childbirth.* 2018;18(1):263.

88. Xiong X, Buekens P, Fraser WD, Guo Z. Anemia during pregnancy in a Chinese population. *Int J Gynaecol Obstet.* 2003;83(2):159-164.

89. Yatich NJ, Funkhouser E, Ehiri JE, et al. Malaria, intestinal helminths and other risk factors for stillbirth in Ghana. *Infect Dis Obstet Gynecol.* 2010;2010:350763.

90. Yi SW, Han YJ, Ohrr H. Anemia before pregnancy and risk of preterm birth, low birth weight and small-for-gestational-age birth in Korean women. *Eur J Clin Nutr.* 2013;67(4):337-342.

91. Zhang J, Cai WW. Risk factors associated with antepartum fetal death. *Early Hum Dev.* 1992;28(3):193-200.

92. Zhang J, Cai WW, Lee DJ. Pregnancy-induced hypertension and early neonatal death: a case-control study. *Am J Perinatol.* 1993;10(5):401-403.

93. Zhang X, Xu Q, Yang Y, et al. Preconception Hb concentration and risk of preterm birth in over 2.7 million Chinese women aged 20-49 years: a population-based cohort study. *Br J Nutr.* 2018;120(5):508-516.

94. Zhang Y, Li Z, Li H, et al. Maternal haemoglobin concentration and risk of preterm birth in a Chinese population. *J Obstet Gynaecol.* 2018;38(1):32-37.

95. Zhou LM, Yang WW, Hua JZ, Deng CQ, Tao X, Stoltzfus RJ. Relation of hemoglobin measured at different times in pregnancy to preterm birth and low birth weight in Shanghai, China. *Am J Epidemiol.* 1998;148(10):998-1006.

96. Abdel-Raoufabdel-Aziz Afifi R, Ali DK, Talkhan HM. Pregnancy outcome and the effect of maternal nutritional status. *J Egypt Soc Parasitol.* 2013;43(1):125-132.

97. Agarwal S, Agarwal A, Bansal AK, Agarwal DK, Agarwal KN. Birth weight patterns in rural undernourished pregnant women. *Indian Pediatr.* 2002;39(3):244-253.

98. Ali EY, Adam GK, Ahmed S, Ali NI, Adam I. Maternal and neonatal hormonal profiles in anaemic pregnant women of eastern Sudan. *J Obstet Gynaecol.* 2009;29(4):311-314.

99. Baraka MA, Steurbaut S, Laubach M, Coomans D, Dupont AG. Iron status, iron supplementation and anemia in pregnancy: ethnic differences. *J Matern Fetal Neonatal Med.* 2012;25(8):1305-1310.

100. Becerra C, Gonzales GF, Villena A, de la Cruz D, Florian A. [Prevalence of anemia in pregnancy, Pucallpa Regional Hospital, Peru]. *Rev Panam Salud Publica.* 1998;3(5):285-292.

101. Caradeux J, Serra R, Palmeiro Y, et al. Correlation between Maternal Characteristics during Early Pregnancy, Fetal Growth Rate and Newborn Weight in Healthy Pregnancies. *Gynecol Obstet Invest.* 2016;81(3):202-206.

102. Demmouche A, Lazrag A, Moulessehoul S. Prevalence of anaemia in pregnant women during the last trimester: consequense for birth weight. *Eur Rev Med Pharmacol Sci.* 2011;15(4):436-445.

103. de Sa SA, Willner E, Duraes Pereira TA, de Souza VR, Teles Boaventura G, Blondet de Azeredo V. ANEMIA IN PREGNANCY: IMPACT ON WEIGHT AND IN THE DEVELOPMENT OF ANEMIA IN NEWBORN. *Nutr Hosp.* 2015;32(5):2071-2079.

104. Dhar B, Mowlah G, Kabir DM. Newborn anthropometry and its relationship with maternal factors. *Bangladesh Med Res Counc Bull.* 2003;29(2):48-58.

105. Duthie SJ, King PA, To WK, Lopes A, Ma HK. A case controlled study of pregnancy complicated by severe maternal anaemia. *Aust N Z J Obstet Gynaecol.* 1991;31(2):125-127.

106. Emamghorashi F, Heidari T. Iron status of babies born to iron-deficient anaemic mothers in an Iranian hospital. *East Mediterr Health J.* 2004;10(6):808-814.

107. Fareh OI, Rizk DE, Thomas L, Berg B. Obstetric impact of anaemia in pregnant women in United Arab Emirates. *J Obstet Gynaecol.* 2005;25(5):440-444.

108. Hasin A, Begum R, Khan MR, Ahmed F. Relationship between birth weight and biochemical measures of maternal nutritional status at delivery in Bangladeshi urban poors. *Int J Food Sci Nutr.* 1996;47(3):273-279.

109. Hassan NE, Shalaan AH, El-Masry SA. Relationship between maternal characteristics and neonatal birth size in Egypt. *East Mediterr Health J.* 2011;17(4):281-289.

110. Jwa SC, Fujiwara T, Yamanobe Y, Kozuka K, Sago H. Changes in maternal hemoglobin during pregnancy and birth outcomes. *BMC Pregnancy Childbirth.* 2015;15:80.

111. Lao TT, Tam KF. Placental ratio and anemia in third-trimester pregnancy. *J Reprod Med.* 2000;45(11):923-928.

112. Laflamme EM. Maternal Hemoglobin Concentration and Pregnancy Outcome: A study of the Effects of Elevation in El Alto, Bolivia. *MJM.* 2010;13(1):47-55.

113. Lee HS, Kim MS, Kim MH, Kim YJ, Kim WY. Iron status and its association with pregnancy outcome in Korean pregnant women. *Eur J Clin Nutr.* 2006;60(9):1130-1135.

114. Lelic M, Bogdanovic G, Ramic S, Brkicevic E. Influence of maternal anemia during pregnancy on placenta and newborns. *Med Arh.* 2014;68(3):184-187.

115. Levario-Carrillo M, Hernandez M, Vasquez ME, Chavez D, Sanchez C, Corral M. [Effects of iron-deficiency anemia on placenta and birth weight]. *Ginecol Obstet Mex.* 2003;71:75-81.

116. Luis J, Fadel MG, Lau GY, Houssein S, Ravikumar N, Yoong W. The effects of severe iron-deficiency anaemia on maternal and neonatal outcomes: A case-control study in an inner-city London hospital. *J Obstet Gynaecol.* 2016;36(4):473-475.

117. Kaur M, Chauhan A, Manzar MD, Rajput MM. Maternal Anaemia and Neonatal Outcome: A Prospective Study on Urban Pregnant Women. *J Clin Diagn Res.* 2015;9(12):Qc04-08.

118. Khoushabi F, Saraswathi G. Impact of nutritional status on birth weight of neonates in Zahedan City, Iran. *Nutr Res Pract.* 2010;4(4):339-344.

119. Koyuncu K, Turgay B, Sukur YE, Yildirim B, Ates C, Soylemez F. Third trimester anemia extends the length of hospital stay after delivery. *Turkish journal of obstetrics and gynecology.* 2017;14(3):166-169.

120. Kuizon MD, Cheong RL, Ancheta LP, Desnacido JA, Macapinlac MP, Baens JS. Effect of anaemia and other maternal characteristics on birthweight. *Hum Nutr Clin Nutr.* 1985;39(6):419-426.

121. Kumar KJ, Asha N, Murthy DS, Sujatha M, Manjunath V. Maternal anemia in various trimesters and its effect on newborn weight and maturity: an observational study. *Int J Prev Med.* 2013;4(2):193-199.

122. Mathews F, Youngman L, Neil A. Maternal circulating nutrient concentrations in pregnancy: implications for birth and placental weights of term infants. *Am J Clin Nutr.* 2004;79(1):103-110.

123. Mezdoud A, Agli AN, Oulamara H. [Relationships between umbilical vein and mother iron status]. *Nutr Hosp.* 2017;34(3):562-567.

124. Mitchell MC, Lerner E. Maternal hematologic measures and pregnancy outcome. *J Am Diet Assoc.* 1992;92(4):484-486.

125. Moghaddam Tabrizi F, Saraswathi G. Maternal anthropometric measurements and other factors: relation with birth weight of neonates. *Nutr Res Pract.* 2012;6(2):132-137.

126. Moghaddam Tabrizi F, Barjasteh S. Maternal Hemoglobin Levels during Pregnancy and their Association with Birth Weight of Neonates. *Iran J Ped Hematol Oncol.* 2015;5(4):211-217.

127. Nahum GG, Stanislaw H. Hemoglobin, altitude and birth weight: does maternal anemia during pregnancy influence fetal growth? *J Reprod Med.* 2004;49(4):297-305.

128. Namli Kalem M, Kalem Z, Akgun N, Yuce E, Aktas H. Investigation of possible maternal and fetal factors which affect umbilical coiling index. *J Matern Fetal Neonatal Med.* 2017:1-7.

129. Ngare DK, Neumann C. Predictors of low birthweight at the community level. *East Afr Med J.* 1998;75(5):296-299.

130. Nordenvall M, Sandstedt B. Placental Less and Maternal Hemoglobin Levels: A Comparative Investigation. *Acta Obstetricia et Gynecologica Scandinavica.* 1990;69(2):127-133.

131. Orlandini C, Torricelli M, Spirito N, et al. Maternal anemia effects during pregnancy on male and female fetuses: are there any differences? *J Matern Fetal Neonatal Med.* 2017;30(14):1704-1708.

132. Rasmussen S, Oian P. First- and second-trimester hemoglobin levels. Relation to birth weight and gestational age. *Acta Obstet Gynecol Scand.* 1993;72(4):246-251.

133. Singh K, Fong YF, Arulkumaran S. Anaemia in pregnancy--a cross-sectional study in Singapore. *Eur J Clin Nutr.* 1998;52(1):65-70.

134. Singla PN, Tyagi M, Kumar A, Dash D, Shankar R. Fetal growth in maternal anaemia. *J Trop Pediatr.* 1997;43(2):89-92.

135. Tarim E, Kilicdag E, Bagis T, Ergin T. High maternal hemoglobin and ferritin values as risk factors for gestational diabetes. *Int J Gynaecol Obstet.* 2004;84(3):259-261.

136. Telatar B, Comert S, Vitrinel A, Erginoz E, Akin Y. The effect of maternal anemia on anthropometric measurements of newborns. *Saudi Med J.* 2009;30(3):409-412.

137. Thame M, Wilks RJ, McFarlane-Anderson N, Bennett FI, Forrester TE. Relationship between maternal nutritional status and infant's weight and body proportions at birth. *Eur J Clin Nutr.* 1997;51(3):134-138.

138. Tunkyi K, Moodley J. Anemia and pregnancy outcomes: a longitudinal study. *J Matern Fetal Neonatal Med.* 2018;31(19):2594-2598.

139. Ugwuja E, Akubugwo E, Ibiam U, Onyechi O. Impact of Maternal Iron Deficiency and Anaemia on Pregnancy and its outcomes in a Nigerian Population. *The Internet Journal of Nutrition and Wellness.* 2009;10(1).

140. Van Bogaert LJ. Anaemia and pregnancy outcomes in a South African rural population. *J Obstet Gynaecol.* 2006;26(7):617-619.

141. von Tempelhoff GF, Heilmann L, Rudig L, Pollow K, Hommel G, Koscielny J. Mean maternal second-trimester hemoglobin concentration and outcome of pregnancy: a population-based study. *Clin Appl Thromb Hemost.* 2008;14(1):19-28.

142. Whittaker PG, Macphail S, Lind T. Serial hematologic changes and pregnancy outcome. *Obstet Gynecol.* 1996;88(1):33-39.

143. Williams LA, Evans SF, Newnham JP. Prospective cohort study of factors influencing the relative weights of the placenta and the newborn infant. *The BMJ.* 1997;314(7098):1864-1868.

144. Yildiz Y, Ozgu E, Unlu SB, Salman B, Eyi EG. The relationship between third trimester maternal hemoglobin and birth weight/length; results from the tertiary center in Turkey. *J Matern Fetal Neonatal Med.* 2014;27(7):729-732.

145. Allen SJ, Raiko A, O'Donnell A, Alexander ND, Clegg JB. Causes of preterm delivery and intrauterine growth retardation in a malaria endemic region of Papua New Guinea. *Arch Dis Child Fetal Neonatal Ed.* 1998;79(2):F135-140.

146. Alwan NA, Cade JE, McArdle HJ, et al. Infant Arterial Stiffness and Maternal Iron Status in Pregnancy: A UK Birth Cohort (Baby VIP Study). *Neonatology.* 2015;107(4):297-303.

147. Arbuckle TE, Sherman GJ. Comparison of the risk factors for pre-term delivery and intrauterine growth retardation. *Paediatr Perinat Epidemiol.* 1989;3(2):115-129.

148. Kumar A, Chaudhary K, Prasad S. Maternal indicators and obstetric outcome in the north Indian population: a hospital-based study. *J Postgrad Med.* 2010;56(3):192-195.

149. Menon KC, Ferguson EL, Thomson CD, et al. Effects of anemia at different stages of gestation on infant outcomes. *Nutrition.* 2016;32(1):61-65.

150. Nansook P, Naidoo RN, Muttoo S, et al. IL-17A[G197G]-Association between NOx and gestational age in a South African birth cohort. *International journal of immunogenetics.* 2018;45(2):54-62.

151. Bhargava A. Modeling the effects of maternal nutritional status and socioeconomic variables on the anthropometric and psychological indicators of Kenyan infants from age 0-6 months. *Am J Phys Anthropol.* 2000;111(1):89-104.

152. Khoigani MG, Goli S, Hasanzadeh A. The relationship of hemoglobin and hematocrit in the first and second half of pregnancy with pregnancy outcome. *Iran J Nurs Midwifery Res.* 2012;17(2 Suppl 1):S165-170.

153. Alwan NA, Cade JE, Greenwood DC, Deanfield J, Lawlor DA. Associations of maternal iron intake and hemoglobin in pregnancy with offspring vascular phenotypes and adiposity at age 10: findings from the Avon Longitudinal Study of Parents and Children. *PLoS One.* 2014;9(1):e84684.

154. Belfort MB, Rifas-Shiman SL, Rich-Edwards JW, Kleinman KP, Oken E, Gillman MW. Maternal iron intake and iron status during pregnancy and child blood pressure at age 3 years. *Int J Epidemiol.* 2008;37(2):301-308.

155. Bergel E, Haelterman E, Belizan J, Villar J, Carroli G. Perinatal factors associated with blood pressure during childhood. *Am J Epidemiol.* 2000;151(6):594-601.

156. Brion MJ, Leary SD, Smith GD, McArdle HJ, Ness AR. Maternal anemia, iron intake in pregnancy, and offspring blood pressure in the Avon Longitudinal Study of Parents and Children. *Am J Clin Nutr.* 2008;88(4):1126-1133.

157. Chou HH, Chiou MJ, Liang FW, Chen LH, Lu TH, Li CY. Association of maternal chronic disease with risk of congenital heart disease in offspring. *CMAJ : Canadian Medical Association Journal.* 2016;188(17-18):E438-e446.

158. Godfrey KM, Forrester T, Barker DJ, et al. Maternal nutritional status in pregnancy and blood pressure in childhood. *Br J Obstet Gynaecol.* 1994;101(5):398-403.

159. Welten M, Gaillard R, Hofman A, de Jonge LL, Jaddoe VW. Maternal haemoglobin levels and cardio-metabolic risk factors in childhood: the Generation R study. *Bjog.* 2015;122(6):805-815.

160. Aranda N, Hernandez-Martinez C, Arija V, Ribot B, Canals J. Haemoconcentration risk at the end of pregnancy: effects on neonatal behaviour. *Public health nutrition.* 2017;20(8):1405-1413.

161. ElAlfy MS, Ali El-Farrash R, Mohammed Taha H, Abdel Rahman Ismail E, Ahmed Mokhtar N. Auditory brainstem response in full-term neonates born to mothers with iron deficiency anemia: relation to disease severity. *J Matern Fetal Neonatal Med.* 2018:1-8.

162. Fararouei M, Robertson C, Whittaker J, et al. Maternal Hb during pregnancy and offspring's educational achievement: a prospective cohort study over 30 years. *Br J Nutr.* 2010;104(9):1363-1368.

163. Mireku MO, Davidson LL, Koura GK, et al. Prenatal Hemoglobin Levels and Early Cognitive and Motor Functions of One-Year-Old Children. *Pediatrics.* 2015;136(1):e76-83.

164. Yang L, Ren AG, Liu JM, Ye RW, Hong SX, Zheng JC. [Influence of hemoglobin level during early gestation on the development of cognition of pre-school children]. *Zhonghua Liu Xing Bing Xue Za Zhi.* 2010;31(12):1353-1358.

165. Ellman LM, Vinogradov S, Kremen WS, et al. Low maternal hemoglobin during pregnancy and diminished neuromotor and neurocognitive performance in offspring with schizophrenia. *Schizophr Res.* 2012;138(1):81-87.

166. Insel BJ, Schaefer CA, McKeague IW, Susser ES, Brown AS. Maternal iron deficiency and the risk of schizophrenia in offspring. *Arch Gen Psychiatry.* 2008;65(10):1136-1144.

167. Nielsen PR, Meyer U, Mortensen PB. Individual and combined effects of maternal anemia and prenatal infection on risk for schizophrenia in offspring. *Schizophr Res.* 2016;172(1-3):35-40.

168. Alemu T, Umeta M. Prevalence and Predictors of "Small Size" Babies in Ethiopia: In-depth Analysis of the Ethiopian Demographic and Health Survey, 2011. *Ethiopian journal of health sciences.* 2016;26(3):243-250.

169. Savajols E, Burguet A, Grimaldi M, Godoy F, Sagot P, Semama DS. Maternal haemoglobin and short-term neonatal outcome in preterm neonates. *PLoS One.* 2014;9(2):e89530.

170. Triche EW, Lundsberg LS, Wickner PG, Belanger K, Leaderer BP, Bracken MB. Association of maternal anemia with increased wheeze and asthma in children. *Ann Allergy Asthma Immunol.* 2011;106(2):131-139.e131.

171. Klonoff-Cohen HS, Srinivasan IP, Edelstein SL. Prenatal and intrapartum events and sudden infant death syndrome. *Paediatr Perinat Epidemiol.* 2002;16(1):82-89.

172. Agarwal DK, Agarwal KN, Satya K, Agarwal S. Weight gain during pregnancy--a key factor in perinatal and infant mortality. *Indian Pediatr.* 1998;35(8):733-743.

173. Shaheen SO, Macdonald-Wallis C, Lawlor DA, Henderson AJ. Haemoglobin concentrations in pregnancy and respiratory and allergic outcomes in childhood: Birth cohort study. *Clinical and experimental allergy : journal of the British Society for Allergy and Clinical Immunology.* 2017;47(12):1615-1624.

174. Stordal K, McArdle HJ, Hayes H, et al. Prenatal iron exposure and childhood type 1 diabetes. *Sci Rep.* 2018;8(1):9067.

175. Babu GR, Murthy GVS, Singh N, et al. Sociodemographic and Medical Risk Factors Associated With Antepartum Depression. *Frontiers in public health.* 2018;6:127.

176. Woldetensay YK, Belachew T, Biesalski HK, et al. The role of nutrition, intimate partner violence and social support in prenatal depressive symptoms in rural Ethiopia: community based birth cohort study. *BMC Pregnancy Childbirth.* 2018;18(1):374.

177. Goshtasebi A, Alizadeh M, Gandevani SB. Association between maternal anaemia and postpartum depression in an urban sample of pregnant women in Iran. *J Health Popul Nutr.* 2013;31(3):398-402.

178. Lukose A, Ramthal A, Thomas T, et al. Nutritional factors associated with antenatal depressive symptoms in the early stage of pregnancy among urban South Indian women. *Matern Child Health J.* 2014;18(1):161-170.

179. Raisanen S, Lehto SM, Nielsen HS, Gissler M, Kramer MR, Heinonen S. Risk factors for and perinatal outcomes of major depression during pregnancy: a population-based analysis during 2002-2010 in Finland. *BMJ Open.* 2014;4(11):e004883.

180. Yilmaz E, Yilmaz Z, Cakmak B, et al. Relationship between anemia and depressive mood in the last trimester of pregnancy. *J Matern Fetal Neonatal Med.* 2017;30(8):977-982.

181. Xu F, Roberts L, Binns C, Sullivan E, Homer CSE. Anaemia and depression before and after birth: a cohort study based on linked population data. *BMC psychiatry.* 2018;18(1):224.

182. Hanprasertpong T, Hanprasertpong J. Pregnancy outcomes in Southeast Asian migrant workers at Southern Thailand. *J Obstet Gynaecol.* 2015;35(6):565-569.

183. Nair M, Kurinczuk JJ, Knight M. Ethnic variations in severe maternal morbidity in the UK- a case control study. *PLoS One.* 2014;9(4):e95086.

184. Azulay CE, Pariente G, Shoham-Vardi I, Kessous R, Sergienko R, Sheiner E. Maternal anemia during pregnancy and subsequent risk for cardiovascular disease. *J Matern Fetal Neonatal Med.* 2015;28(15):1762-1765.

185. Daru J, Zamora J, Fernandez-Felix BM, et al. Risk of maternal mortality in women with severe anaemia during pregnancy and post partum: a multilevel analysis. *Lancet Glob Health.* 2018;6(5):e548-e554.

186. Buchmann EJ, Mnyani CN, Frank KA, Chersich MF, McIntyre JA. Declining maternal mortality in the face of persistently high HIV prevalence in a middle-income country. *Bjog.* 2015;122(2):220-227.

187. Bloch EM, Crookes RL, Hull J, et al. The impact of human immunodeficiency virus infection on obstetric hemorrhage and blood transfusion in South Africa. *Transfusion.* 2015;55(7):1675-1684.

188. Chatterjee A, Bosch RJ, Hunter DJ, Fataki MR, Msamanga GI, Fawzi WW. Maternal disease stage and child undernutrition in relation to mortality among children born to HIV-infected women in Tanzania. *J Acquir Immune Defic Syndr.* 2007;46(5):599-606.

189. Ezechi OC, Gab-Okafor CV, Oladele DA, et al. Prevalence and risk factors of asymptomatic bacteriuria among pregnant Nigerians infected with HIV. *J Matern Fetal Neonatal Med.* 2013;26(4):402-406.

190. Li N, Matchi E, Spiegelman D, et al. Maternal mortality among HIV-infected pregnant women in Tanzania. *Acta Obstet Gynecol Scand.* 2014;93(5):463-468.

191. Liotta G, Mancinelli S, Nielsen-Saines K, et al. Reduction of maternal mortality with highly active antiretroviral therapy in a large cohort of HIV-infected pregnant women in Malawi and Mozambique. *PLoS One.* 2013;8(8):e71653.

192. Machado ES, Krauss MR, Megazzini K, et al. Hypertension, preeclampsia and eclampsia among HIV-infected pregnant women from Latin America and Caribbean countries. *J Infect.* 2014;68(6):572-580.

193. O'Brien ME, Kupka R, Msamanga GI, Saathoff E, Hunter DJ, Fawzi WW. Anemia is an independent predictor of mortality and immunologic progression of disease among women with HIV in Tanzania. *J Acquir Immune Defic Syndr.* 2005;40(2):219-225.

194. Obimbo EM, Mbori-Ngacha DA, Ochieng JO, et al. Predictors of early mortality in a cohort of human immunodeficiency virus type 1-infected african children. *Pediatr Infect Dis J.* 2004;23(6):536-543.

195. Traisathit P, Mary JY, Le Coeur S, et al. Risk factors of preterm delivery in HIV-infected pregnant women receiving zidovudine for the prevention of perinatal HIV. *J Obstet Gynaecol Res.* 2009;35(2):225-233.

196. van den Broek NR, Jean-Baptiste R, Neilson JP. Factors associated with preterm, early preterm and late preterm birth in Malawi. *PLoS One.* 2014;9(3):e90128.

197. Young S, Murray K, Mwesigwa J, et al. Maternal nutritional status predicts adverse birth outcomes among HIV-infected rural Ugandan women receiving combination antiretroviral therapy. *PLoS One.* 2012;7(8):e41934.

198. Alizadeh L, Raoofi A, Salehi L, Ramzi M. Impact of maternal hemoglobin concentration on fetal outcomes in adolescent pregnant women. *Iran Red Crescent Med J.* 2014;16(8):e19670.

199. al-Sibai MH, Khwaja SS, al-Suleiman SA, Magbool G. The low birth-weight infants of Saudi adolescents: maternal implications. *Aust N Z J Obstet Gynaecol.* 1987;27(4):320-322.

200. Chang SC, O'Brien KO, Nathanson MS, Mancini J, Witter FR. Hemoglobin concentrations influence birth outcomes in pregnant African-American adolescents. *J Nutr.* 2003;133(7):2348-2355.

201. Pinho-Pompeu M, Surita FG, Pastore DA, Paulino DSM, Pinto ESJL. Anemia in pregnant adolescents: impact of treatment on perinatal outcomes. *J Matern Fetal Neonatal Med.* 2017;30(10):1158-1162.

202. Scholl TO, Decker E, Karp RJ, Greene G, De Sales M. Early adolescent pregnancy: a comparative study of pregnancy outcome in young adolescents and mature women. *J Adolesc Health Care.* 1984;5(3):167-171.

203. Amburgey OA, Ing E, Badger GJ, Bernstein IM. Maternal hemoglobin concentration and its association with birth weight in newborns of mothers with preeclampsia. *J Matern Fetal Neonatal Med.* 2009;22(9):740-744.

204. Sak ME, Evsen MS, Soydinc HE, et al. Risk factors for maternal mortality in eclampsia: analysis of 167 eclamptic cases. *Eur Rev Med Pharmacol Sci.* 2012;16(10):1399-1403.

205. Kosto A, Okby R, Levy M, Sergienko R, Sheiner E. The effect of maternal anemia on maternal and neonatal outcomes in twin pregnancies. *J Matern Fetal Neonatal Med.* 2016;29(14):2297-2300.

206. Shumpert MN, Salihu HM, Kirby RS. Impact of maternal anaemia on birth outcomes of teen twin pregnancies: a comparative analysis with mature young mothers. *J Obstet Gynaecol.* 2004;24(1):16-21.

207. Berhan Y. Predictors of perinatal mortality associated with placenta previa and placental abruption: an experience from a low income country. *J Pregnancy.* 2014;2014:307043.

208. Bo L, Mei-Ying L, Yang Z, Shan-Mi W, Xiao-Hong Z. Aplastic anemia associated with pregnancy: maternal and fetal complications. *J Matern Fetal Neonatal Med.* 2016;29(7):1120-1124.

209. Cyganek A, Pietrzak B, Kociszewska-Najman B, et al. Intrauterine growth restriction in pregnant renal and liver transplant recipients: risk factors assessment. *Transplant Proc.* 2014;46(8):2794-2797.

210. Sehgal R, Kriplani A, Vanamail P, Maiti L, Kandpal S, Kumar N. Assessment and comparison of pregnancy outcome among anaemic and non anaemic primigravida mothers. *Indian J Public Health.* 2016;60(3):188-194.

211. Yang Z, Mei-Ying L, Shan-Mi W, Xiao-Hui Z. Pregnancy and myelodysplastic syndrome: an analysis of the clinical characteristics, maternal and fetal outcomes. *J Matern Fetal Neonatal Med.* 2015;28(18):2155-2159.

212. Zaren B, Lindmark G, Bergsjo P. Hemoconcentration in smoking mothers is associated with impaired fetal growth. *Acta Obstet Gynecol Scand.* 1997;76(10):933-941.

213. Bakhtiar UJ, Khan Y, Nasar R. Relationship between maternal hemoglobin and perinatal outcome. *RMJ.* 2007;32(2):102-104.

214. Bhalerao A, Kawthalkar A, Ghike S, Joshi S. Anemia during Pregnancy: Most Preventable yet most Prevalent. *Journal of South Asian Federation of Obstetrics and Gynaecology.* 2011;3(2):75-77.

215. Hirve SS, Ganatra BR. Determinants of low birth weight: a community based prospective cohort study. *Indian Pediatr.* 1994;31(10):1221-1225.

216. Shobeiri F, Begum K, Nazari M. A prospective study of maternal hemoglobin status of Indian women during pregnancy and pregnancy outcome. *Nutrition Research.* 2006;26(5):209-213.

217. Murphy JF, O'Riordan J, Newcombe RG, Coles EC, Pearson JF. Relation of haemoglobin levels in first and second trimesters to outcome of pregnancy. *Lancet.* 1986;1(8488):992-995.

218. Ahmad Z, Jr., Jaafar R, Mohd Hassan M, Othman M, Hashim A. Anaemia during pregnancy in rural Kelantan. *Malays J Nutr.* 1997;3(1):83-90.

219. Bakacak M, Avci F, Ercan O, et al. The effect of maternal hemoglobin concentration on fetal birth weight according to trimesters. *J Matern Fetal Neonatal Med.* 2015;28(17):2106-2110.

220. Misra A, Ray S, Patrikar S. A longitudinal study to determine association of various maternal factors with neonatal birth weight at a tertiary care hospital. *Med J Armed Forces India.* 2015;71(3):270-273.

221. Yu HF, Wang YX, Li L, et al. Survey and analysis on birth quality influence factors of 300 cases of newborns. *Clin Exp Obstet Gynecol.* 2014;41(2):121-123.

222. Onadeko MO, Avokey F, Lawoyin TO. Observations of stillbirths, birthweight and maternal haemoglobin in teenage pregnancy in Ibadan, Nigeria. *Afr J Med Med Sci.* 1996;25(1):81-86.

223. Lin L, Wei Y, Zhu W, et al. Prevalence, risk factors and associated adverse pregnancy outcomes of anaemia in Chinese pregnant women: a multicentre retrospective study. *BMC Pregnancy Childbirth.* 2018;18(1):111.

224. Hosain GM, Chatterjee N, Begum A, Saha SC. Factors associated with low birthweight in rural Bangladesh. *J Trop Pediatr.* 2006;52(2):87-91.

225. Sekhavat L, Davar R, Hosseinidezoki S. Relationship between maternal hemoglobin concentration and neonatal birth weight. *Hematology.* 2011;16(6):373-376.

226. Kidanto HL, Mogren I, Lindmark G, Massawe S, Nystrom L. Risks for preterm delivery and low birth weight are independently increased by severity of maternal anaemia. *S Afr Med J.* 2009;99(2):98-102.

227. Oladeinde HB, Oladeinde OB, Omoregie R, Onifade AA. Prevalence and determinants of low birth weight: the situation in a traditional birth home in Benin City, Nigeria. *Afr Health Sci.* 2015;15(4):1123-1129.

228. Singh G, Chouhan R, Sidhu K. Maternal Factors for Low Birth Weight Babies. *Med J Armed Forces India.* 2009;65(1):10-12.

229. Castaldi JL, Bertin MS, Gimenez F, Lede R. [Periodontal disease: Is it a risk factor for premature labor, low birth weight or preeclampsia?]. *Rev Panam Salud Publica.* 2006;19(4):253-258.

230. Brabin BJ, Ginny M, Sapau J, Galme K, Paino J. Consequences of maternal anaemia on outcome of pregnancy in a malaria endemic area in Papua New Guinea. *Annals of tropical medicine and parasitology.* 1990;84(1):11-24.

231. Ahankari AS, Myles PR, Dixit JV, Tata LJ, Fogarty AW. Risk factors for maternal anaemia and low birth weight in pregnant women living in rural India: a prospective cohort study. *Public health.* 2017;151:63-73.

232. Gardner H, Green K, Gardner AS, Geddes D. Observations on the health of infants at a time of rapid societal change: a longitudinal study from birth to fifteen months in Abu Dhabi. *BMC pediatrics.* 2018;18(1):32.

233. Cordina M, Bhatti S, Fernandez M, Syngelaki A, Nicolaides KH, Kametas NA. Association between maternal haemoglobin at 27-29weeks gestation and intrauterine growth restriction. *Pregnancy Hypertens.* 2015;5(4):339-345.

234. Raghu Raman TS, Parimala V, Bhalla M, Venkateshwar V, Iyengar A. A CORRELATIVE STUDY OF MATERNAL HAEMOGLOBIN AND BIRTH WEIGHT. *Med J Armed Forces India.* 2001;57(2):110-113.

235. Yazdani M, Tadbiri M, Shakeri S. Maternal hemoglobin level, prematurity, and low birth weight. *Int J Gynaecol Obstet.* 2004;85(2):163-164.

236. Burrows RF. Maternal characteristics and their influences on birth weight in a Melanesian population. *Asia Oceania J Obstet Gynaecol.* 1988;14(1):1-5.

237. Ma AG, Schouten E, Wang Y, et al. Anemia prevalence among pregnant women and birth weight in five areas in China. *Med Princ Pract.* 2009;18(5):368-372.

238. Mitchell MC, Lerner E. Factors that influence the outcome of pregnancy in middle-class women. *J Am Diet Assoc.* 1987;87(6):731-735.

239. Suryanarayana R, Chandrappa M, Santhuram AN, Prathima S, Sheela SR. Prospective study on prevalence of anemia of pregnant women and its outcome: A community based study. *Journal of family medicine and primary care.* 2017;6(4):739-743.

240. Rizvi SA, Hatcher J, Jehan I, Qureshi R. Maternal risk factors associated with low birth weight in Karachi: a case-control study. *East Mediterr Health J.* 2007;13(6):1343-1352.

241. Stanisic DI, Moore KA, Baiwog F, et al. Risk factors for malaria and adverse birth outcomes in a prospective cohort of pregnant women resident in a high malaria transmission area of Papua New Guinea. *Trans R Soc Trop Med Hyg.* 2015;109(5):313-324.

242. Yuan W, Duffner AM, Chen L, Hunt LP, Sellers SM, Bernal AL. Analysis of preterm deliveries below 35 weeks' gestation in a tertiary referral hospital in the UK. A case-control survey. *BMC Res Notes.* 2010;3:119.

243. Weidinger H, Wiest W. A comparative study of the epidemiological data of pregnancies with and without tendencies to premature delivery. *J Perinat Med.* 1974;2(4):276-287.

244. Sullivan AD, Nyirenda T, Cullinan T, et al. Malaria infection during pregnancy: intrauterine growth retardation and preterm delivery in Malawi. *J Infect Dis.* 1999;179(6):1580-1583.

245. Beta J, Issat T, Nowicka MA, Andziak M, Jakimiuk AJ. Early spontaneous preterm deliveries before 34 weeks' gestation in a tertiary care centre: analysis of maternal factors and obstetric history. *J Matern Fetal Neonatal Med.* 2013;26(7):720-723.

246. Tabussum G, Karim SA, Khan S, Naru TY. Preterm birth--its etiology and outcome. *J Pak Med Assoc.* 1994;44(3):68-70.

247. Abrams ET, Milner DA, Jr., Kwiek J, et al. Risk factors and mechanisms of preterm delivery in Malawi. *Am J Reprod Immunol.* 2004;52(2):174-183.

248. Fahim HI, Abdel Maeboud KH, Ashour HA, el-Makhzangy MN. A study of the epidemiology of preterm labor. *J Egypt Public Health Assoc.* 1992;67(3-4):341-355.

249. Wagura P, Wasunna A, Laving A, Wamalwa D, Ng'ang'a P. Prevalence and factors associated with preterm birth at kenyatta national hospital. *BMC Pregnancy Childbirth.* 2018;18(1):107.

250. Zhang Q, Ananth CV, Li Z, Smulian JC. Maternal anaemia and preterm birth: a prospective cohort study. *Int J Epidemiol.* 2009;38(5):1380-1389.

251. Dincgez Cakmak B, Turker UA, Oztas S, Arik M, Ustunyurt E. The effect of first trimester hemoglobin levels on pregnancy outcomes. *Turkish journal of obstetrics and gynecology.* 2018;15(3):165-170.

252. Jehan I, McClure EM, Salat S, et al. Stillbirths in an urban community in Pakistan. *Am J Obstet Gynecol.* 2007;197(3):257.e251-258.

253. Tomashek KM, Ananth CV, Cogswell ME. Risk of stillbirth in relation to maternal haemoglobin concentration during pregnancy. *Matern Child Nutr.* 2006;2(1):19-28.

254. Zhang Q, Ananth CV, Rhoads GG, Li Z. The impact of maternal anemia on perinatal mortality: a population-based, prospective cohort study in China. *Ann Epidemiol.* 2009;19(11):793-799.

255. Little MP, Brocard P, Elliott P, Steer PJ. Hemoglobin concentration in pregnancy and perinatal mortality: a London-based cohort study. *Am J Obstet Gynecol.* 2005;193(1):220-226.

256. Nair M, Knight M, Robinson S, Nelson-Piercy C, Stanworth SJ, Churchill D. Pathways of association between maternal haemoglobin and stillbirth: path-analysis of maternity data from two hospitals in England. *BMJ Open.* 2018;8(4):e020149.

257. Mau G. Hemoglobin changes during pregnancy and growth disturbances in the neonate. *J Perinat Med.* 1977;5(4):172-177.

258. Sawant LD, Venkat S. Comparative Analysis of Normal versus Fetal Growth Restriction in Pregnancy: The Significance of Maternal Body Mass Index, Nutritional Status, Anemia, and Ultrasonography Screening. *International journal of reproductive medicine.* 2013;2013:671954.

259. Narang A, Chaudhuri MK, Kumar P. Small for gestational age babies: Indian scene. *Indian J Pediatr.* 1997;64(2):221-224.

260. Chhabra S, Bhandari V. Some medico-socio-demographic factors and intra-uterine growth retardation. *J Indian Med Assoc.* 1996;94(4):127-130.

261. Serour GI, Younis NM, Hefnawi F, El-Bahy M, Dagistany HF, Nawar M. Perinatal mortality in an Egyptian maternity hospital. *Int J Gynaecol Obstet.* 1981;19(6):447-451.

262. Rubio-Alvarez A, Molina-Alarcon M, Arias-Arias A, Hernandez-Martinez A. Development and validation of a predictive model for excessive postpartum blood loss: A retrospective, cohort study. *International journal of nursing studies.* 2018;79:114-121.

263. Kavle JA, Stoltzfus RJ, Witter F, Tielsch JM, Khalfan SS, Caulfield LE. Association between anaemia during pregnancy and blood loss at and after delivery among women with vaginal births in Pemba Island, Zanzibar, Tanzania. *J Health Popul Nutr.* 2008;26(2):232-240.

264. Biguzzi E, Franchi F, Ambrogi F, et al. Risk factors for postpartum hemorrhage in a cohort of 6011 Italian women. *Thromb Res.* 2012;129(4):e1-7.

265. Petty K, Waters JH, Sakamoto SB, Yazer MH. Antenatal anemia increases the risk of receiving postpartum red blood cell transfusions although the overall risk of transfusion is low. *Transfusion.* 2018;58(2):360-365.

266. Morgan-Ortiz F, Calderon-Lara SA, Martinez-Felix JI, Gonzalez-Beltran A, Quevedo-Castro E. [Risk factors associated with preeclampsia: case-control study]. *Ginecol Obstet Mex.* 2010;78(3):153-159.

267. Mehrabian F, Hosseini SM. Comparison of gestational diabetes mellitus and pre-eclampsia in women with high hemoglobin in the first trimester of pregnancy: A longitudinal study. *Pak J Med Sci.* 2013;29(4):986-990.

268. Cordina M, Bhatti S, Fernandez M, Syngelaki A, Nicolaides KH, Kametas NA. Maternal hemoglobin at 27-29 weeks' gestation and severity of pre-eclampsia. *J Matern Fetal Neonatal Med.* 2015;28(13):1575-1580.

269. Nombo AP, Mwanri AW, Brouwer-Brolsma EM, Ramaiya KL, Feskens EJM. Gestational diabetes mellitus risk score: A practical tool to predict gestational diabetes mellitus risk in Tanzania. *Diabetes research and clinical practice.* 2018.

270. Alshareef SA, Rayis DA, Adam I, Gasim GI. Helicobacter pylori infection, gestational diabetes mellitus and insulin resistance among pregnant Sudanese women. *BMC Res Notes.* 2018;11(1):517.

271. Macaulay S, Ngobeni M, Dunger DB, Norris SA. The prevalence of gestational diabetes mellitus amongst black South African women is a public health concern. *Diabetes research and clinical practice.* 2018;139:278-287.

272. Nastaran SA, Nourossadat K, Abbas HF, Hamid AM. Hemoglobin level during the first trimester of pregnancy in gestational diabetes. *Ginekol Pol.* 2012;83(12):929-933.
